# MetaSee: An Interactive and Extendable Visualization Toolbox for Metagenomic Sample Analysis and Comparison

**DOI:** 10.1371/journal.pone.0048998

**Published:** 2012-11-08

**Authors:** Baoxing Song, Xiaoquan Su, Jian Xu, Kang Ning

**Affiliations:** 1 BioEnergy Genome Center, Qingdao Institute of Bioenergy and Bioprocess Technology, Chinese Academy of Sciences, Qingdao, Shandong Province, People’s Republic of China; 2 Shandong Key Laboratory of Energy Genetics, Qingdao Institute of Bioenergy and Bioprocess Technology, Chinese Academy of Sciences, Qingdao, Shandong Province, People’s Republic of China; 3 CAS Key Laboratory of Biofuels, Qingdao Institute of Bioenergy and Bioprocess Technology, Chinese Academy of Sciences, Qingdao, Shandong Province, People’s Republic of China; J. Craig Venter Institute, United States of America

## Abstract

The NGS (next generation sequencing)-based metagenomic data analysis is becoming the mainstream for the study of microbial communities. Faced with a large amount of data in metagenomic research, effective data visualization is important for scientists to effectively explore, interpret and manipulate such rich information. The visualization of the metagenomic data, especially multi-sample data, is one of the most critical challenges. The different data sample sources, sequencing approaches and heterogeneous data formats make robust and seamless data visualization difficult. Moreover, researchers have different focuses on metagenomic studies: taxonomical or functional, sample-centric or genome-centric, single sample or multiple samples, etc. However, current efforts in metagenomic data visualization cannot fulfill all of these needs, and it is extremely hard to organize all of these visualization effects in a systematic manner. An extendable, interactive visualization tool would be the method of choice to fulfill all of these visualization needs. In this paper, we have present MetaSee, an extendable toolbox that facilitates the interactive visualization of metagenomic samples of interests. The main components of MetaSee include: (I) a core visualization engine that is composed of different views for comparison of multiple samples: Global view, Phylogenetic view, Sample view and Taxa view, as well as link-out for more in-depth analysis; (II) front-end user interface with real metagenomic models that connect to the above core visualization engine and (III) open-source portal for the development of plug-ins for MetaSee. This integrative visualization tool not only provides the visualization effects, but also enables researchers to perform in-depth analysis of the metagenomic samples of interests. Moreover, its open-source portal allows for the design of plug-ins for MetaSee, which would facilitate the development of any additional visualization effects.

## Introduction

Microbes are everywhere around us on the planet, and the total number of microbial cells on earth is huge [1]. Microbes usually live in communities, and each of these communities has different community structures and functions. As such, microbial communities would serve as the largest reservoir of genes and genetic functions for a large number of applications in bio-related disciplines, including biomedicine in healthcare, bioenergy, bioremediation and biodefense [2]. Since more than 90% of strains found in the microbial communities cannot be isolated and cultivated [3], metagenomic methods have been used to analyze the microbial community as a whole.

### 1 The Importance and Challenges in Metagenomic Data Analysis

Understanding the taxonomical structure of a microbial community (alpha diversity) and the differences in taxon among microbial communities (beta diversity) have been two of the most important problems in metagenomic research [4], [5]. For alpha diversity, questions regarding the relative abundance of different taxa across multiple levels are the focuses of research. Beta diversity focuses on the comparison of community structures of different microbial communities, which is especially important to find the complex relationships among a large number of samples. Understanding the beta diversity is critical for studying microbial ecology. For example, Human Microbiome Projects [6] and related efforts to study microbial communities occupying various human body habitats have shown a surprising amount of diversities among individuals in skin [7], [8], gut [9], and mouth ecosystems [10], [11].

Advances in sequencing technologies have equipped researchers with the ability to sequence collective genomes of entire microbial communities, commonly referred to as metagenome, in an inexpensive and high-throughput manner [12]. Thus, a rapidly increasing number of metagenomic profiles of microbial communities have been archived in public repositories and research labs around the world. Mining these data would be important for the revealing of the intricate relationship among samples. Therefore, it is becoming more and more important to compare microbial communities in large scale.

### 2 The Needs of a Platform for Interactive Visualization of Metagenomic Samples

Extensive collaborations between microbiologists and bioinformaticians are needed for large-scale metagenomic data analysis & interpretation. To facilitate their collaboration, an easy to use and cross-platform system for interactive visualization of metagenome is urgently needed.

Firstly, although many metagenomic data analysis tasks can be accomplished with automated processes, some steps continue to require human judgments and are frequently rate limiting, for example the comparison between two or more samples. Visualization can augment our ability to reason about complex data, and increase the efficiency of manual analyses. Given the importance of human interpretation, visualization tools also provide a valuable complement to automated computational techniques, particularly in the early hypothesis generation stages of biological research, enabling us to derive scientific insight from large-scale data sets [13]. An effective method of visualization should display the data in such a way that the answers to common questions become obvious [14].

Secondly, researchers need high-quality figures to facilitate their in-depth analysis and interpretation of their work. And it is important for software designers continue providing scientists with tools that are useful, effective and illustrative [15].

Finally, current metagenomic research is becoming a multi-region, multi-discipline and multi-expertise collaboration effort, with many researchers working on microbiology-related energy, medicine, environment, etc. The common theme for these researches is that the data are produced around the world but analyzed in a data analysis center. As such, the need for a cross-platform visualization toolbox to serve such collaborations is becoming more and more urgent.

### 3 Representing Metagenomic Data from Different Angles are Beyond the Ability of Current Stand-alone Visualization Tools

Metagenomic samples are usually presented in a kind of diverse hierarchy, for which there are only a few of visualization tools designed. In addition, they need to be examined from different angles and levels: phylogeny information, taxonomical structure and functional structure. However, current visualization tools are limited by their abilities to show only one or two angles for the metagenomic taxonomical samples of interests.

Current metagenomic visualization tools could be categorized as independent or dependent (as a component in comprehensive software) by their dependencies on other software, or as open-source or closed-source by their software distribution strategy.

**Figure 1:**
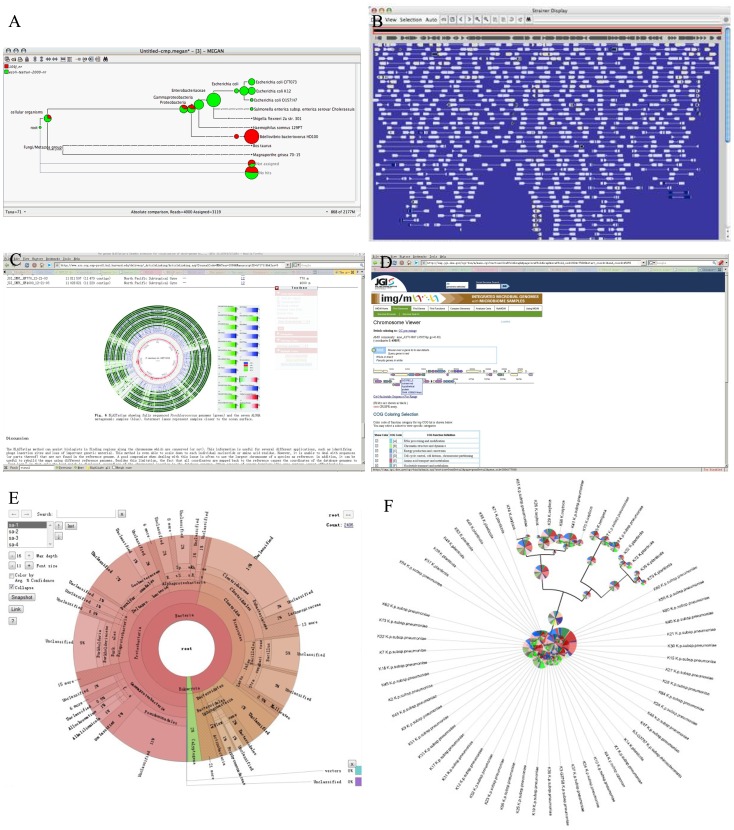
Screenshot of current metagenomic visualization effects. (A) The visualization result of MEGAN with taxonomy components of two metagenomic samples. (B) The visualization user interface of Strainer (The visualization of components of metagenomics in micro-perspective). The comparison between a component of a metagenome sample and a genome. (C) The visualization user interface of BLASTatla (The visualization of components of metagenomics in macro-perspective: The comparison among a component of metagenome and multiple genomes). (D) Contig and gene annotation visualization of IMG/M. (E) The visualization user interface of Krona. This is the user interface of comparison of four saliva microbiomes. (F) The metagenome visualization result of iTOL with default parameters.

Based on the NCBI Taxonomy database, MEGAN [16] could display the components of taxonomy of one or more metagenomic samples **(** **Figure 1(A)** **)**, whereas it is not an open source or independent application so its visualization part could not be easily imported into other applications.

Strainer [17] **(** **Figure 1(B)** **)** and BLASTatlas [18] **(** **Figure 1(C)** **)** could display and compare genome structures and annotations in the form of micro- and macro- genomic views separately. But these perspectives are limited in comparison between two genomes or between genomes and metagenomes, which can’t provide the comparison of multiple metagenomic samples.

IMG/M [19] has a visualization tool developed by DOE Joint Genome Institute aimed to compare metagenomes. While IMG/M performs very well at multiple levels, including functional annotation, classification, etc., it can only provide visualization effect for contigs and its annotations are from a single metagenomic sample **(** **Figure 1(D)** **)**. Other information, such as the comparison of community structures among multiple metagenomes, is provided as simple tables. Therefore, in IMG/M, much of the important information (e.g. comparison among multiple metagenomic samples) is missing in visualized results.

Krona [14] **(** **Figure 1(E)** **)** is another metagenomic visualization tool. It allows hierarchical data to be explored with zoom-able pie charts and it is good at displaying the structure of single samples. Although Krona can display multiple samples, it could only display one sample in a window at a time. So it is non-intuitive to find the difference between/among multiple samples at specific taxa.

iTOL [20] can provide a global view for multiple metagenome samples **(** **Figure 1(F)** **)**, but it needs a dataset file, which is not generated by iTOL but has to be written manually. Furthermore, iTOL is not open source and there was also no binary files provided for offline use.

There are several other visualization tools for metagenomic sample visualization [21], [22]. But generally, various levels of granularities inherent in these classifications and multiple samples pose challenges for visualization. Tree-structure diagrams can be used to convey hierarchy [23], and bar or pie charts can display the relative abundances at specific levels, but neither of these methods alone creates a complete figure for metagenomic analysis. Additionally, taxonomic and functional hierarchies are often too complex for all nodes to be shown. Furthermore, there are substantial biological variations among different metagenomes and specific shifts in time series, which are beyond the ability of currently available open source visualization tools.

The foundation of most metagenomic studies is the assignment of observed nucleic acids to taxonomic or functional hierarchies [24], [25]. Metagenomic classification algorithms are constantly improving, but their results still come with a significant degree of uncertainty. Only a small fraction of the tree of life is represented in reference databases, and this causes widespread bias in classification [26]. But, apart from Krona [14], other visualization tools would only provide non-comprehensive information about which part was classified, or at which level reads were classified. Therefore, currently, there is not an open-source or independent tool that could provide visualization solution that includes: (1) The phylogenic information of the metagenomic samples, (2) The illustrative comparison of multiple metagenomic samples by their microbial community structures and functions, (3) Details about how precisely each branch was classified and at which level each node (taxa) is. Though MEGAN [16] could provide most of the above functions; it is mostly closed-source, making it almost impossible to include the visualization part of MEGAN [16] into other applications or online services.

It should be noticed that some of the visualization effects required by users could not be easily realized on a single interface clearly. For example, it would not be clear to have multiple metagenome comparison results placed in the same page. For such visualization effects, a substantial integration and interactive visualization tools might be the method of choice.

### 4 Our Approach – MetaSee, an Interactive Metagenomic Viewer

In this work, we have developed an integrative visualization system, MetaSee, based on most advanced computer visualization techniques. The MetaSee system is composed of (1) the core visualization engine, (2) the front-end interactive analysis interface, and (3) the API portal for plugin development.

(1) The core visualization engine includes: visualization of the taxonomical structure of the metagenomic samples globally and at different levels, comparison of different metagenomic samples and link-out to different annotations for the taxa and/or functions, etc. (2) The front-end user interface is specifically designed for real metagenomic models (such as the oral microbial community models) that connect to the above core visualization engine. And (3) the open-source API portal for the development of plug-in is designed for easy-extension of the MetaSee system.

## Methods and Implementations

MetaSee is implemented based on all of the taxonomical and functional information that could be retrieved from metagenomic samples, and takes advantage of modern computer visualization technology, including HTML5 canvas, JavaScript, SVG and modern web browsers. The only requirement for viewing the result of MetaSee is an updated web browser, and the results can be viewed (online or off-line) on almost all operating systems (OS) with Graphical User Interface (GUI).

### 1 High-performance Computational Backbone

Visualization tools are particularly powerful when used in combination with high-throughput automated analysis software (e.g., Parallel-META [27], [28]). Features, such as easy-to-use, cross OS platform and open source of MetaSee, make it easy to build this visualization tool in high-throughput automated analysis pipelines. In this work, we have used Parallel-META [27], [28] to analyze the metagenomic data, and the interactive visualization effects were built based on these results.

### 2 The Core Visualization Engine

**Figure 2:**
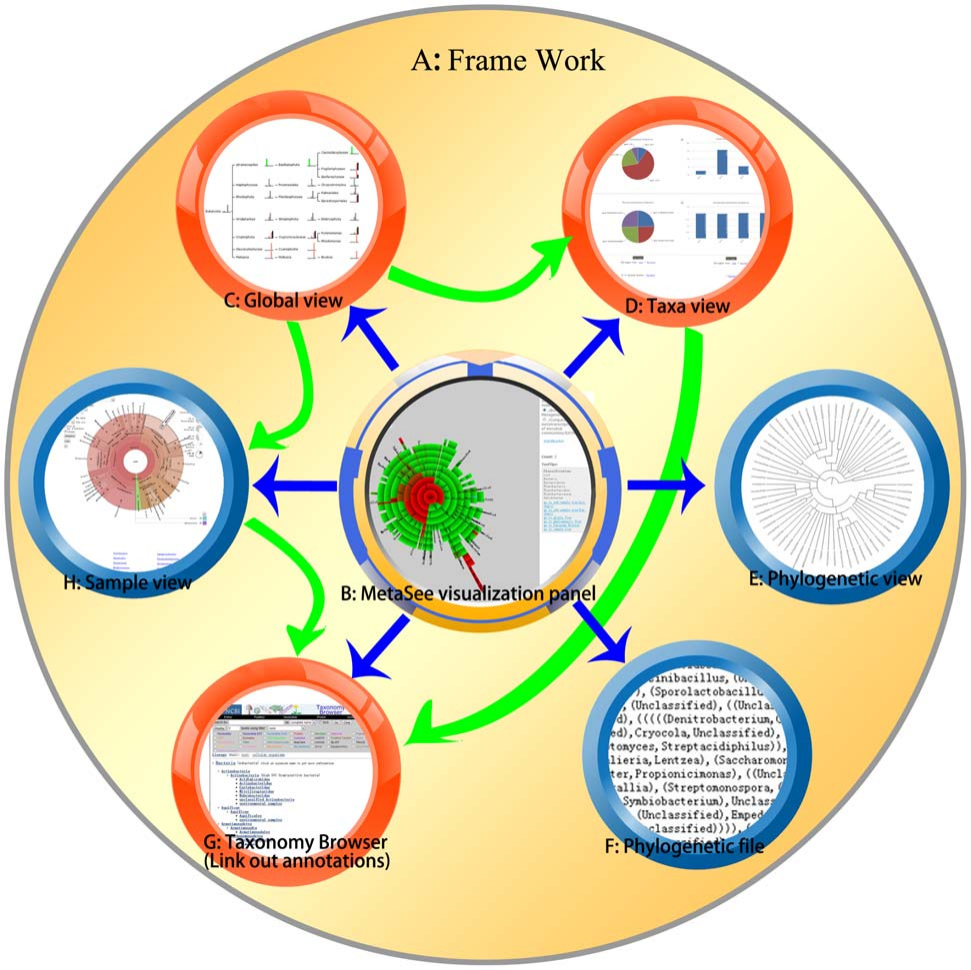
Overview of the components of MetaSee. Each pie stands for an element of MetaSee, and directed arrow stand for a front-end link from one component to another component.

The core visualization engine is composed of multiple viewing components: the viewing components include (not exclusive of each other): overall framework (**Figure 2(A)**), MetaSee visualization panel (**Figure 2(B)**), Global view (**Figure 2(C)**), Taxa view (**Figure 2(D)**), Phylogenetic view (**Figure 2(E)**), Phylogenetic file (**Figure 2(F)**), Link out annotations (**Figure 2(G)**) and Sample view (**Figure 2(H)**). These components are capable of providing GUI for visualization of uploaded files, and aim to answer questions regarding the relative abundance of taxa across multiple levels of the hierarchy for multiple samples simultaneously.

#### (1) Framework (Figure 3)

**Figure 3:**
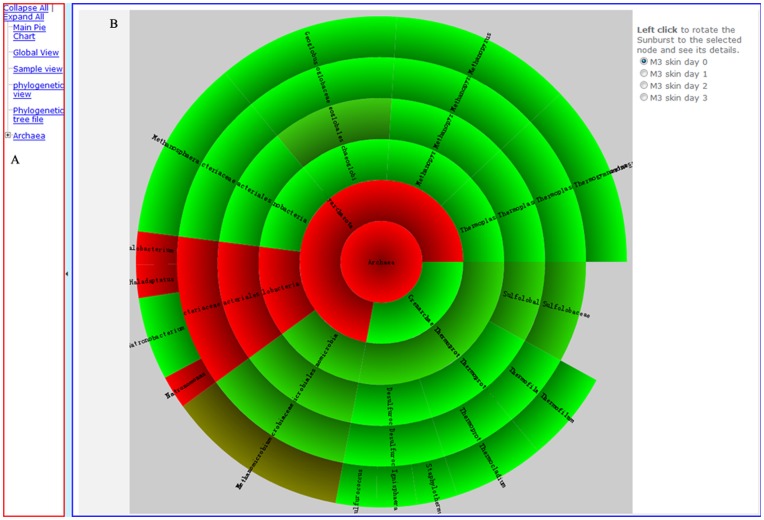
Overview of the visualization result of MetaSee and the Framework. (A) Left side bar for navigation, (B) Main window is the working area for visualization.

The framework includes the left sidebar **(** **Figure 3(A)** **)** and the main window **(** **Figure 3(B)** **)**. The left sidebar is the navigation bar for visualization results, which can be flipped on and off. The main window is the working area and all the views will be displayed in this area.

#### (2) MetaSee visualization panel (Figure 2(B))

The MetaSee visualization panel is the main interactive operation panel of MetaSee, which is designed for interactive analysis of the structure of metagenome. This panel is a pie chart, and when a sector (representing a taxa) of it is selected, the area will be highlighted and turn to the right. The right side bar of MetaSee visualization panel will display the detailed information of this node and links to other views. The lengths of layers of these charts indicate which part of this dataset was classified more precisely. And the color of each sector indicates the abundance of this sector (taxa) (red color indicates more abundant taxa).

#### (3) Global view (Figure 2(C))

For each sample, a Global view is a hierarchical tree that contains every taxa and their proportion in the sample. Two or more samples can be shown in a single Global view, with each node composed of a bar-plot showing the relative abundance of different samples at those taxa. Thus, Global view shows the whole picture of all samples being compared. In Global view, all the taxonomy units at the same level are in the same rank, so it is easy to find which part of the input dataset was enriched (classified with more details). The heights of each pillar stand for the relative abundance of each sample at this taxonomy unit. The detail information of a certain taxonomy unit is linked from small bar chart to their Taxa view (a pair of pie-charts and a pair of bar-charts) with relative abundance, absolutely abundance and legend. In Global view each color indicates a sample (as indicated in figure legend), and it is convenient to find the difference among multiple samples at the global level of a certain taxonomy unit.

#### (4) Taxa view (Figure 2(D))

For one or a set of samples, the Taxa view focuses on the detail information of one node (taxa) in Global view, a taxonomical hierarchy tree structure (by clicking the bar-plot for that node). This detailed information includes the abundance information at the specific taxa, which is useful for comparing different samples for specific taxa. It can be shown in either pie-chart or bar-chart format.

#### (5) Phylogenetic view (Figure 2(E))

For each sample, a Phylogenetic view is an unweighted phylogenetic tree. It elucidates the evolutionary relationship of all microbes in a microbiome community.

#### (6) Phylogenetic tree file (Figure 2(F))

Unweighted phylogenetic tree file is presented in Newick format. It can also be imported into other phylogenetic tree visualization tool (e.g. Phylogenetic tree Maker (http://www.metasee.org/visualizationlab/phylogenetictrees.jsp) ).

#### (7) Sample view (Figure 2(H))

For each sample, the taxonomical community structure is represented in a dynamic multi-layer pie-chart, so that each taxon’s (at each level) proportion can be vividly seen by interactively zoomed-in or zoomed-out. Moreover, pie-charts for multi-samples can be smoothly shifted from one to another for comparison of structure and proportion. The sample view is implemented by the Krona software [14]. The Sample view can also be viewed directly or linked from the Global view (by clicking the legend box at the up-right corner).

#### (8) Link-out annotation (Figure 2(G))

Each of the taxa or function could be linked-out to their annotation from external sources from MetaSee visualization panel, Global view (by clicking the name of that node), Sample view or Taxa view. Here we use the taxonomy browser database of NCBI [29] as external link-out annotation source, which would facilitate digging the detailed information of a certain taxa and speeding up the manual analysis process.

### 3 Multi-sample Comparisons

**Figure 4:**
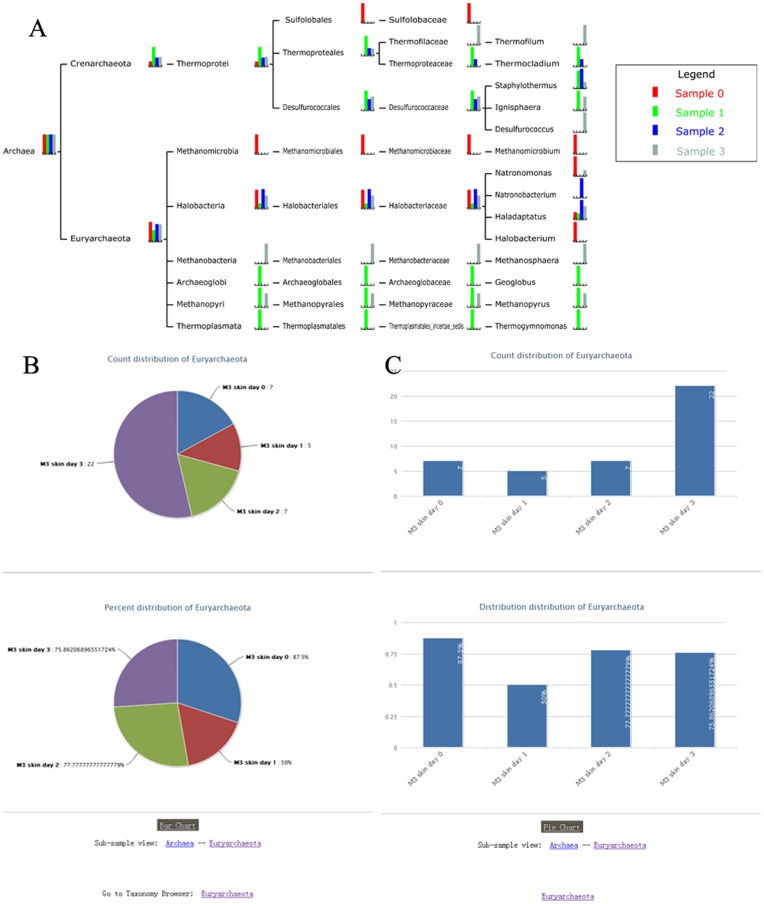
Comparison across multiple samples. (A) Global view, (B) Taxa view with pie-chart format, (C) Taxa view with bar-chart format.

Metagenomic data are often generated at discrete points across multiple locations or times. MetaSee is able to store the data from multiple samples in a single framework. Individual samples may then be stepped through. Thus, it makes the comparison among samples coming from different time points or conditions easy (**Figure 4**). In Global view (**Figure 4(A)**), the bars with the same color come from the same sample, and the height of each pillar represents the relative abundance of corresponding samples at corresponding nodes (taxa). Taxa view (**Figure 4(B)** **and** **Figure 4(C)**) includes a pie-chart and a bar-chart for each node, and both pie chart and bar chart have two graphs to represent the relative abundance and absolute number, respectively. In addition to these visualization functions, to provide high quality graph for publication purpose, all the graphs produced by MetaSee are vector graphs.

### 4 The Front-end Interactive Analysis Interface

The front-end interface mainly serves for a set of real metagenome projects based on MetaSee visualization system. Two areas may need this metagenomic visualization system: dentistry and field experimental studies. For dentists, this system would help them for quick diagnosis by using the novel samples that have been collected as queries to search in the database of known samples of microbial communities. This has been proven to be workable for dentists so far [11]. For field study experts such as those on ocean expeditions or doing soil sample testing, this tool would help them to analyze their data quickly and get illustrative results easily. For these two areas, we have designed two interfaces, “Digital mouth” and “Metagenome global survey”, as examples of front-end.

### 5 Open-source Portals for Plug-in Development

Open-source portals were designed to extend the usability of the MetaSee visualization system. Firstly, community structure files in many formats can be imported into MetaSee. As XML is easy to expand, it was selected as the default format. Yet, during run time, community structure files in many formats could be stored in random access memory (RAM) as double linked trees, by an independent component for tree building. Based on this design model, it is very easy to develop other APIs for new input file formats. As examples, we developed APIs for importing output files from parallel-META [27], [28], MEGAN [16] and MG-RAST [22]. And other APIs for input data manipulations are under development.

Secondly, the work flow of MetaSee could build a tree structure and then output this tree to a variety of views. Therefore, adding new APIs for other views (such as back-to-back sample views) or modifying existing views would be facilitated.

Thirdly, the search function of the MetaSee toolbox (http://www.metasee.org/visualizationlab/search/) provided a portal for searching any metagenomic samples against a metagenome database. Right now only samples from “Metagenome global survey” could be searched against a pre-built database of annotated metagenomic samples (just to show-case its functions). Yet this open-source portal could facilitate the re-development of search functions to search any user-specified metagenomic sample against any metagenome database.

Finally, we have established a repository (http://www.metasee.org/tools.jsp and http://www.metasee.org/laboratory.jsp) to provide more viewing options and more viewing services. Other APIs, such as those for online tools and database connections, are under development.

### 6 Online Web Services and Resources

The online version (http://www.metasee.org) of MetaSee accepts files of many formats and when a file is uploaded, a GUI will be produced. Users can analyze the resulting dynamic graph online and also download it. Additionally, high quality vector graphs could be used for publication purpose.

**Figure 5:**
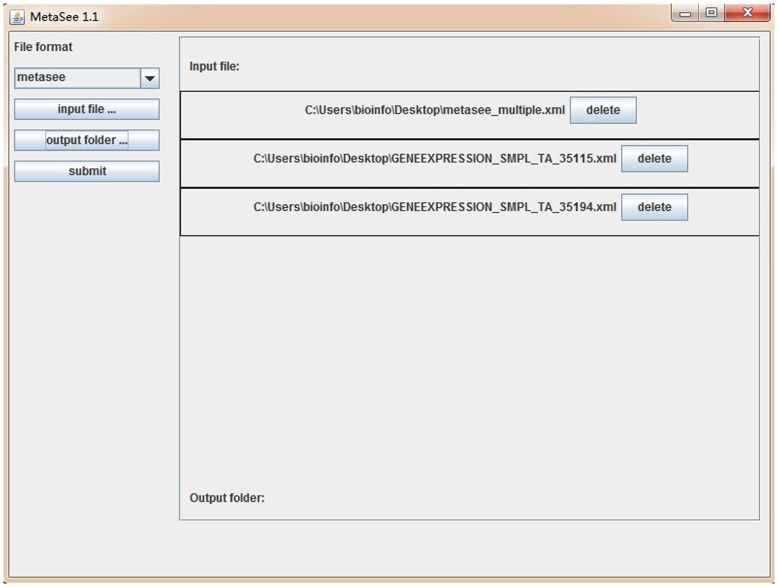
The GUI of standalone version of MetaSee. Firstly, select the format of input data with the drop-down list. Secondly, click the “input file” to select input file, multiple files can be accepted, but these files should be in uniform format. Thirdly, press the “output folder” button to assign the output path. Finally, press “submit” button to run MetaSee.

Additionally, stand-alone MetaSee application could be downloaded as a virtual machine, which was developed in Java, and can run almost on all OS using both GUI (**Figure 5**) and command line. As MetaSee is multi-threaded, it can accept a very large dataset. The output result is a set of HTML pages with high-resolution figures.

We released our source codes and development documents. With these documents, example source codes, sample data and our discussion group (http://groups.google.com/group/metasee), developer can develop new APIs of MetaSee for their purposes, and we would like to accept the codes contributed by other developers.

## Results and Discussion

To evaluate the ability of metagenome visualization, we compared MetaSee with other metagenomic visualization tools based on 450 metagenomic samples [30], [31] (most of the analysis results of these data can be found at http://www.metasee.org/visualizationlab/map/).

### 1 Comparison with Existing Metagenome Visualization Tools

MetaSee is designed to augment our ability to reason about complex data. In Framework and Global view, MetaSee ranked nodes neatly, making the hierarchy of taxa within metagenomic data self-evident. MetaSee also provided a solution for the visualization of multiple sample metagenomic dataset and makes the comparison among metagenome in a visualized manner possible.

Here we take the visualization of four saliva metagenomic samples [11] as the example data, and compare MetaSee against other metagenomic visualization tools. As for the biological background of these four samples [11], the study’s focuses include: 1)the difference between caries-active and healthy human populations, to find some taxa that may distinguish caries-active from healthy human populations, 2) whether there is a organismal core and the phylogenetic diversity between two caries-active or two healthy human, 3) the difference of community structures between two kind of samples. Details about these 4 samples can be found in Table S1.

The Strainer [32] and BlastTaslas [18] cannot provide the comparison of multiple metagenomic samples and IMG/M [19] provides the comparison of evolutional relationship and structure among multiple metagenomic samples in simple tabular format. Krona [14] is not good at visualization of multiple samples. Producing a multi-sample metagenomic image with iTOL [20] is difficult because there is no supporting API. MEGAN [16], [33] has most of the visualization functions as described above, but it is not an open-source visualization tools. Details about these comparisons can be found in Text S1.

As show-cases of the front-ends of MetaSee, we developed two applications: Digital Mouth and Metagenome global survey.

### 2 Application on Digital Mouth Metagenomic Samples

There have been several studies on different oral microbial communities, and a series of reports have been published showing their relationship with oral disease, like gingivitis, periodontitis, caries, *et al*. More and more dentists or those who work related to oral healthy are focusing on microbiome etiological factors involved in disease [34]. Our previous studies suggested that caries-associated microbiomes were significantly more variable in community structure whereas the healthy ones were relatively conserved. And abundance changes of certain taxa may distinguish caries microbiota from healthy ones [31]. However, as microbes in oral system function as members of complex multi-taxa communities, we can’t understand the characteristics of specific microbiota from a long list of members. What we need is a macroscopic and stereotype visualization on the complex microbiota.

In this work, we focus on the construction of “Digital Mouth” from two perspectives: firstly, the 3D structure of the mouth itself; and secondly, the microbial community’s structure in oral samples (http://www.metasee.org/visualizationlab/mouth/). In association with the 3D structure of the mouth, the visualization of microbial community data would provide visualization of a dental environment for in-depth mouth analysis by dentists, especially those for analyzing the microbes in the mouth.

### 3 Application on Metagenome Global Survey Samples

Following the idea that nature could be understood by reducing its complexity to the molecular level and analyzing the interactions between a small number of molecules to explain simple causal relationships; systems biology has been the modern approach to understand what life is. With the development of sampling technology, some expeditions that carried out a comprehensive worldwide sample collection campaign with a coherent strategy to record all the information necessary to the study of the emergent properties of plankton ecosystems emerged and a new concept “Oceans Systems Biology” [35] was created. With it, a need for analyzing this type of metagenome data also emerged.

In this work, we tried to visualize these data with MetaSee. We collected 380 metagenomic samples with Geographic Information System(GIS) information, and analyzed them with Parallel-META [28] and marked them on a Google map. User can click the markers on the map, to check them and then compare them by MetaSee (http://www.metasee.org/visualizationlab/map/).

### Conclusion

The visualization of the metagenomic samples has been proven to be very important to augment our ability to increase the efficiency of manual analyses. However, metagenomic data is very complex, and generally the focus of researchers are the similarities as well as differences among multiple samples, so the visualization of metagenomic data is a difficult work. MetaSee partially solves this problem based on an interactive and dynamic visualization toolbox.

The MetaSee toolbox that is proposed in this paper is an easy to use, interactive, cross platform data visualization toolbox. It addresses the problem of comparing multiple metagenomic samples. Moreover, the open-source portal for plug-in development has enabled the modification and embedding it in other applications possible.

When the WGS (whole genome sequencing)-based sequencing coverage is deep enough, *de novo* or reference-guided genome assembly could be performed. Then we can add more annotation information on every node (taxa/function). With the flexible interface and a variety of input files, MetaSee will be the method of choice for completing this task.

Additionally, MetaSee is not only for metagenomics. It is a flexible framework that can also take other dataset of tree structure as input and give beautiful visualization results. Examples of these data would include global health statistics data from WHO (http://www.who.int/gho/database/en/) and Global bioenergy survey data (http://iea.org/stats/balancetable.asp?COUNTRY_CODE=CN).

### Availability

**Licenses:**

MetaSee was released under The MIT License

The standalone version and online service of MetaSee could be found at: http://www.metasee.org/

URL of Digital Mouth: http://www.metasee.org/visualizationlab/mouth/

URL of Metagenome global survey: http://www.metasee.org/visualizationlab/map/.

## Supporting Information

Table S1 **Information of four saliva metagenomic samples**
(DOCX)

Text S1 **Comparison with existing metagenome visualization tools.** Krona is not good at visualization of multiple samples. Producing a multi-sample metagenomic image with iTOL is difficult because there is no supporting API. MEGAN has powerful visualization module, but it is not open-source.
(DOCX)
