# Supplementary material for: MetaSee: An Interactive and Extendable Visualization Toolbox for Metagenomic Sample Analysis and Comparison
Source: PLoS One. 2012 Nov 8;7(11):e48998. doi: 10.1371/journal.pone.0048998 (PMC3493548; doi:10.1371/journal.pone.0048998)
Supplement: Table S1 — Information of four saliva metagenomic samples (DOCX) [file pone.0048998.s001.docx]

**Table S1 Information of four saliva metagenomic samples**

| ID | Group | DMFT Index | Gender | Age | Number of reads for 16sRNA |
| --- | --- | --- | --- | --- | --- |
|  |  |  |  |  |  |
| Healthy Saliva Sample 1 | Healthy | 0 | Female | 18 | 4351 |
| Healthy Saliva Sample 2 | Healthy | 0 | Male | 20 | 2776 |
| Decayed Saliva Sample 1 | Caries-active | 7 | Male | 18 | 43294 |
| Decayed Saliva Sample 2 | Caries-active | 9 | Male | 18 | 1514 |
