# Supplementary material for: MetaSee: An Interactive and Extendable Visualization Toolbox for Metagenomic Sample Analysis and Comparison
Source: PLoS One. 2012 Nov 8;7(11):e48998. doi: 10.1371/journal.pone.0048998 (PMC3493548; doi:10.1371/journal.pone.0048998)
Supplement: Text S1 — Comparison with existing metagenome visualization tools. Krona is not good at visualization of multiple samples. Producing a multi-sample metagenomic image with iTOL is difficult because there is no supporting API. MEGAN has powerful visualization module, but it is not open-source. (DOCX) [file pone.0048998.s002.docx]

# Supporting Online Materials for “MetaSee: An interactive and extendable visualization toolbox for metagenomic sample analysis and comparison”

[Supporting Online Materials for “MetaSee: An interactive and extendable visualization toolbox for metagenomic sample analysis and comparison” 1](#_Toc337630754)

[Introduction 1](#_Toc337630755)

[The visualization result 2](#_Toc337630756)

[Result of Krona 2](#_Toc337630757)

[Result of iTOL 4](#_Toc337630758)

[Result of MEGAN 6](#_Toc337630759)

[Result of MetaSee 7](#_Toc337630760)

[Appendix 11](#_Toc337630761)

[File used for iTOL 11](#_Toc337630762)

[Tree file 11](#_Toc337630763)

[Dataset file 12](#_Toc337630764)

[File used for MEGAN 26](#_Toc337630765)

[Reference 66](#_Toc337630766)

# Introduction

To evaluate the ability of MetaSee to visualize metagenomics, here we take the visualization results of four saliva metagenomic samples [[1](#_ENREF_1)] as the example data, and compare MetaSee against other metagenomic visualization tool. As for the biological background [[1](#_ENREF_1)], the study’s focuses include: 1) the difference between caries-active and healthy human populations, and find some taxa that may distinguish caries-active from healthy human populations, 2) whether there is a organismal core and the phylogenetic diversity between two caries-active or two healthy human, 3) the difference of community structure between two kind of samples. There are 4 samples used in [[1](#_ENREF_1)], with details as in Table S1.

The Strainer[[2](#_ENREF_2)] and BlastTaslas[[3](#_ENREF_3)] can’t provide the comparison of multiple metagenomics and IMG/M[[4](#_ENREF_4)] provide the comparison of evolutional relationship and structure among multiple metagenomics by table. So here we visualization the analysis result of Parallel-META[[5](#_ENREF_5)] with MetaSee, Krona[[6](#_ENREF_6)], iTOL[[7](#_ENREF_7)] and MEGAN[[8](#_ENREF_8)].

Firstly, we get the gDNA sequence of these four saliva dataset and analyzed them with Parallel-META, when do sequence alignment, we set 1e-10 as the E-value and selected greengenes[[9](#_ENREF_9)] as reference database, the command just like: parallel-meta -m c2.fa -o ggc2 -d $ParallelMETA/databases/gg_coreset -e 1e-10.

Secondly we input the result file (classification.txt) to every visualization tool separately.

Finally we compared the visualization result of these tools, and try to get some useful information of these four dataset.

# The visualization result

## Result of Krona

The Krona is a great tool to show the structure of single sample, it can show the detail information of a specific node; the max depth also can be set.


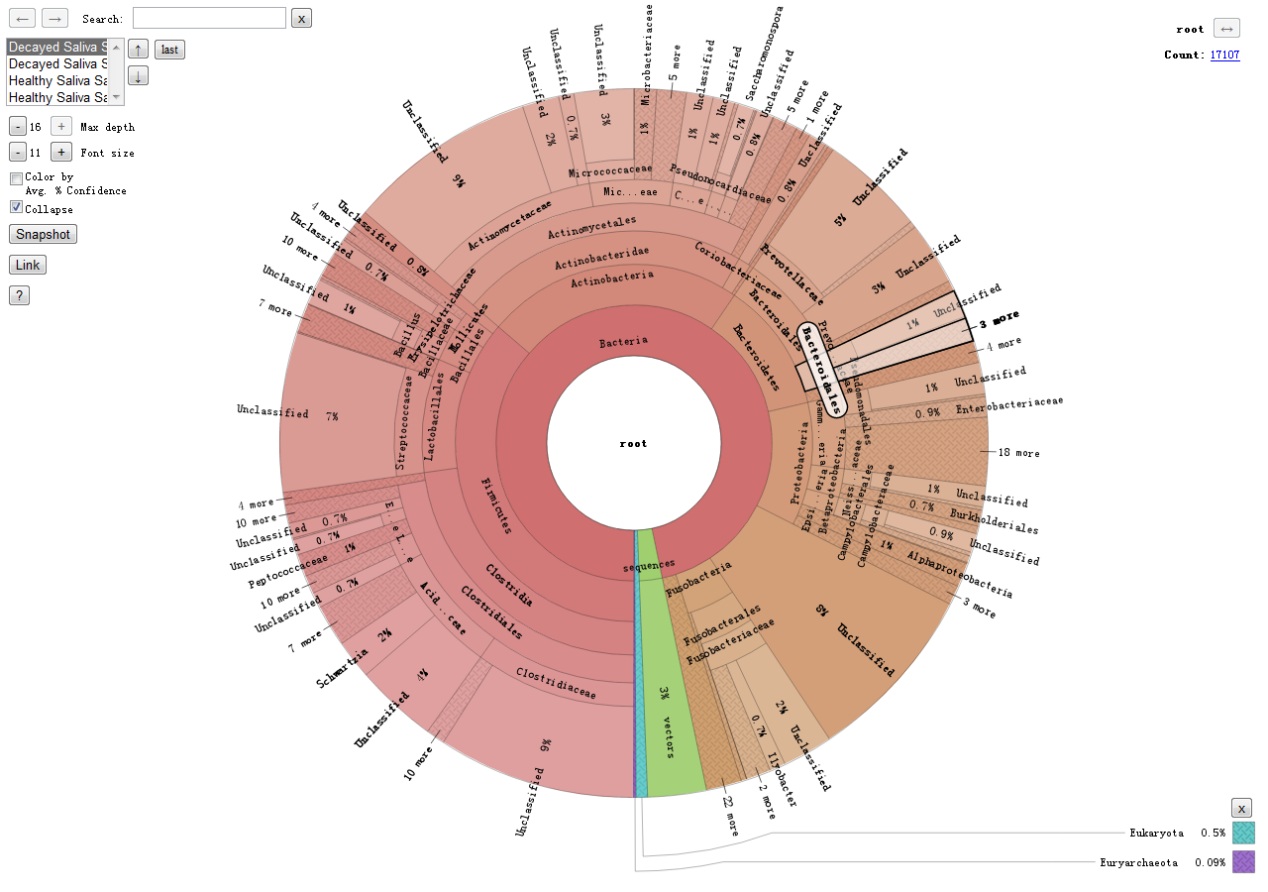


Figure S1 (A) The visualization result of the Krona of Decayed Saliva Sample 1.


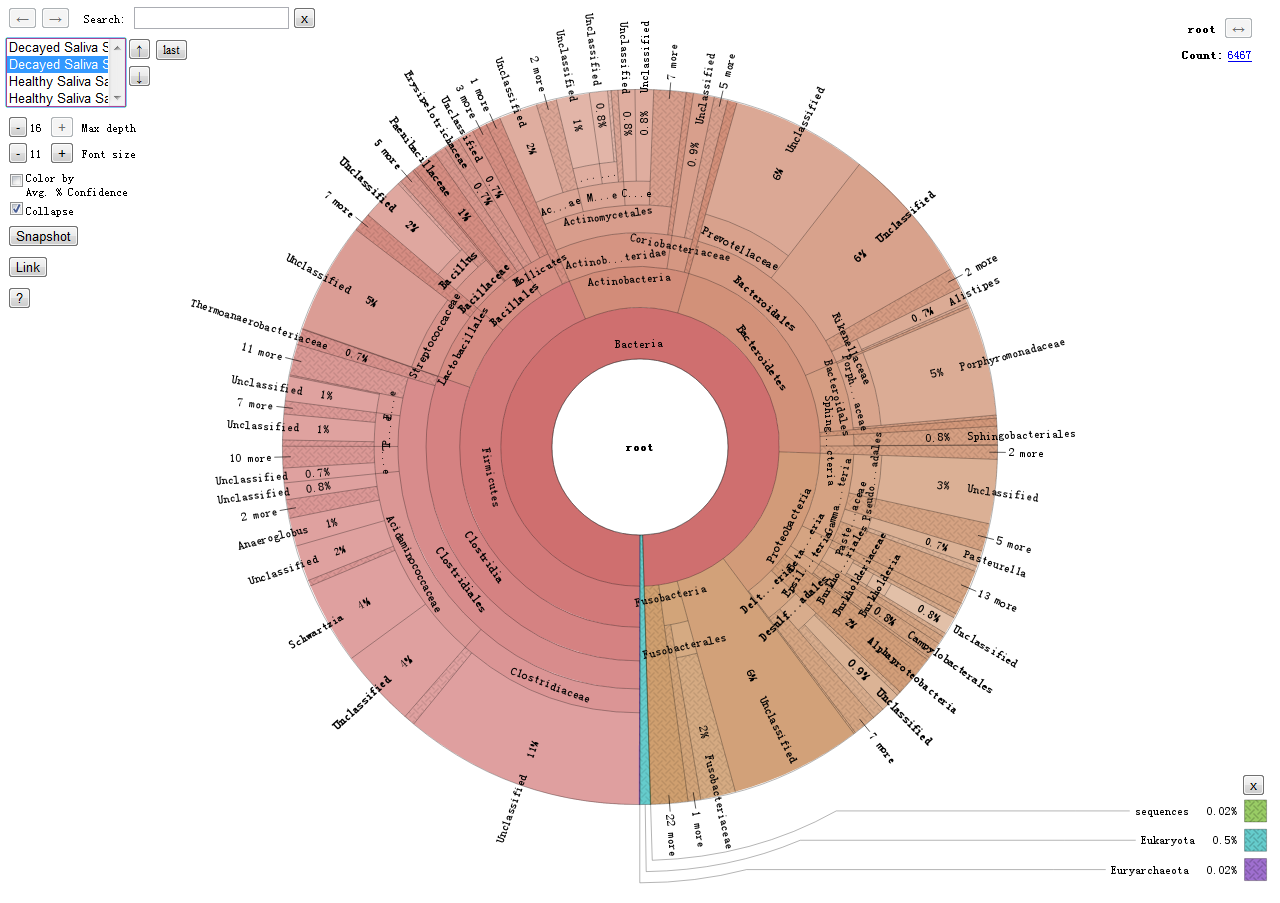


Figure S1 (B) The visualization result of the Krona of Decayed Saliva Sample 2.


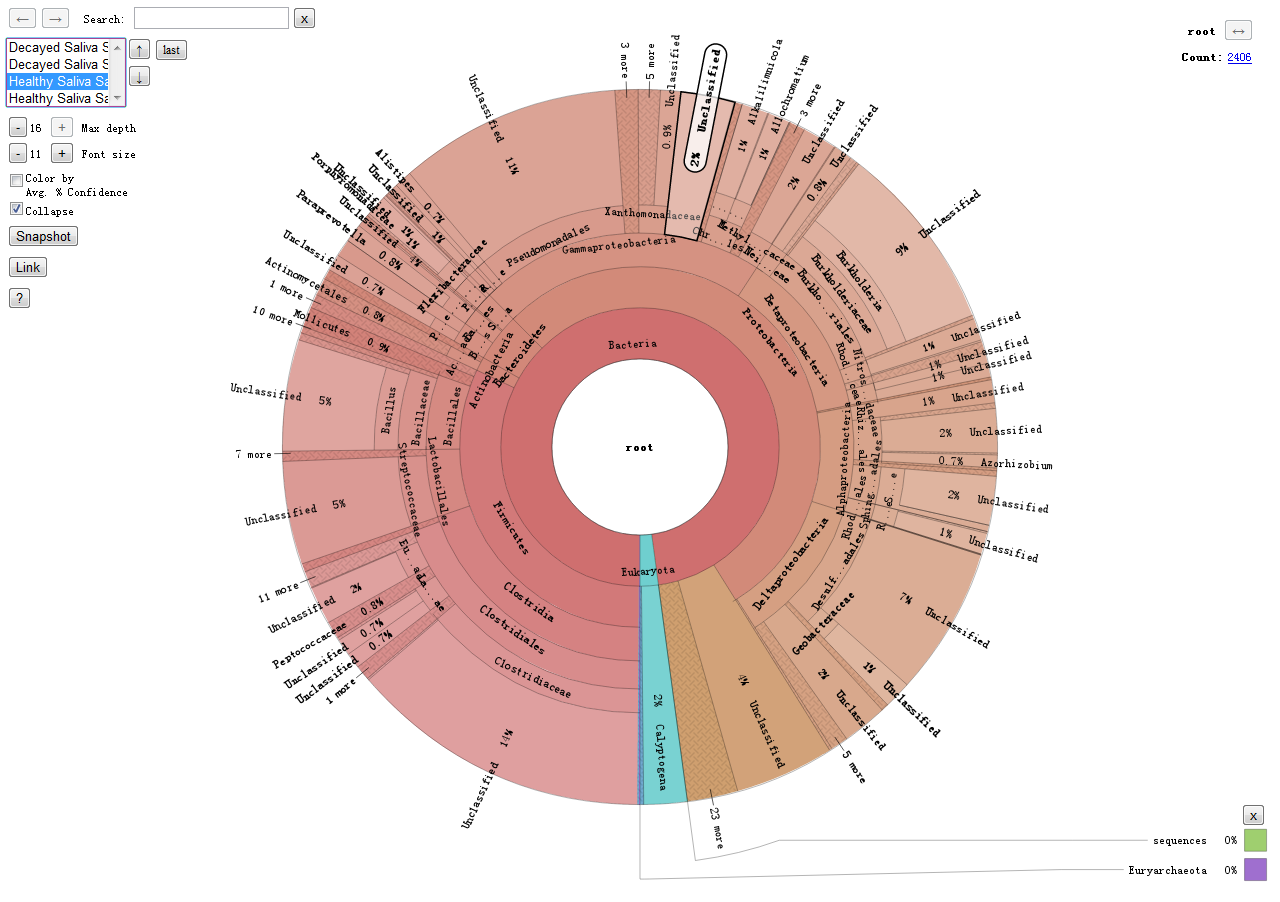


Figure S1 (C) The visualization result of the Krona of Healthy Saliva Sample 1


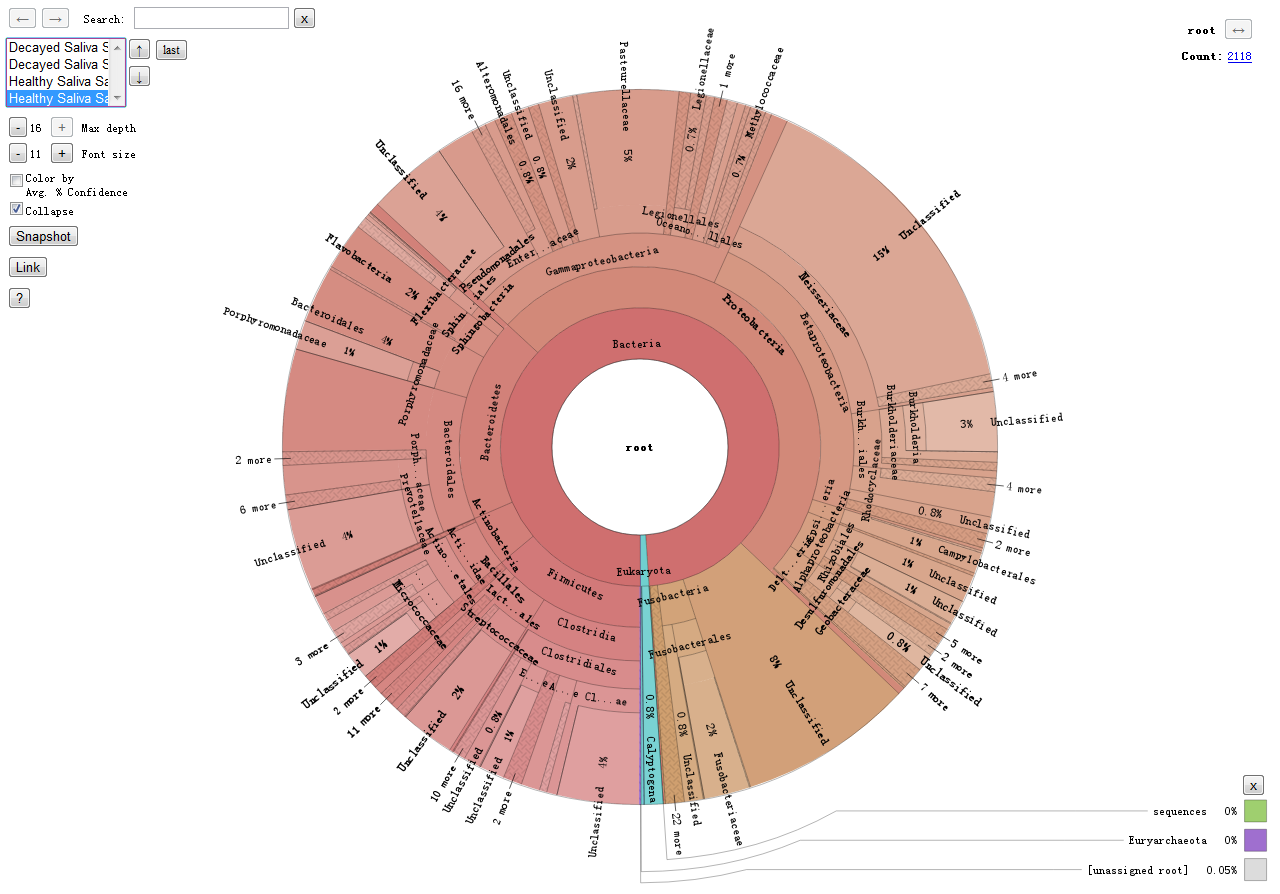


Figure S1 (D) The visualization result of the Krona of Healthy Saliva Sample 2.

But when showing multiple metagenomic samples, it can’t provide a “real” multiple samples visualization result. Though can switch among visualizations of different samples dynamically and interactively, it is still difficult to be used to compare multiple samples.

## Result of iTOL

Though it can be used to visualize metagenomic data, the iTOL was not designed for the visualization of this kind of data. And it will miss a lot of information of middle nodes. What’s more it can only be used to visualization of small metagenomic dataset or else it will be a hard work to use the visualization result to analysis the metagenomic data. Finally, there have no API for the producing of dataset file, needed by iTOL, from other popular metagenomic analysis software.


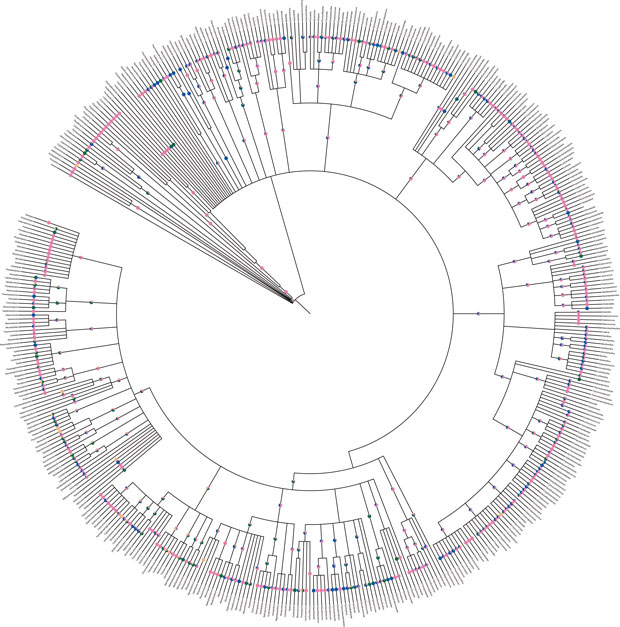


Figure S2 (A) The visualization result of the iTOL of comparison four saliva metagenomic dataset.


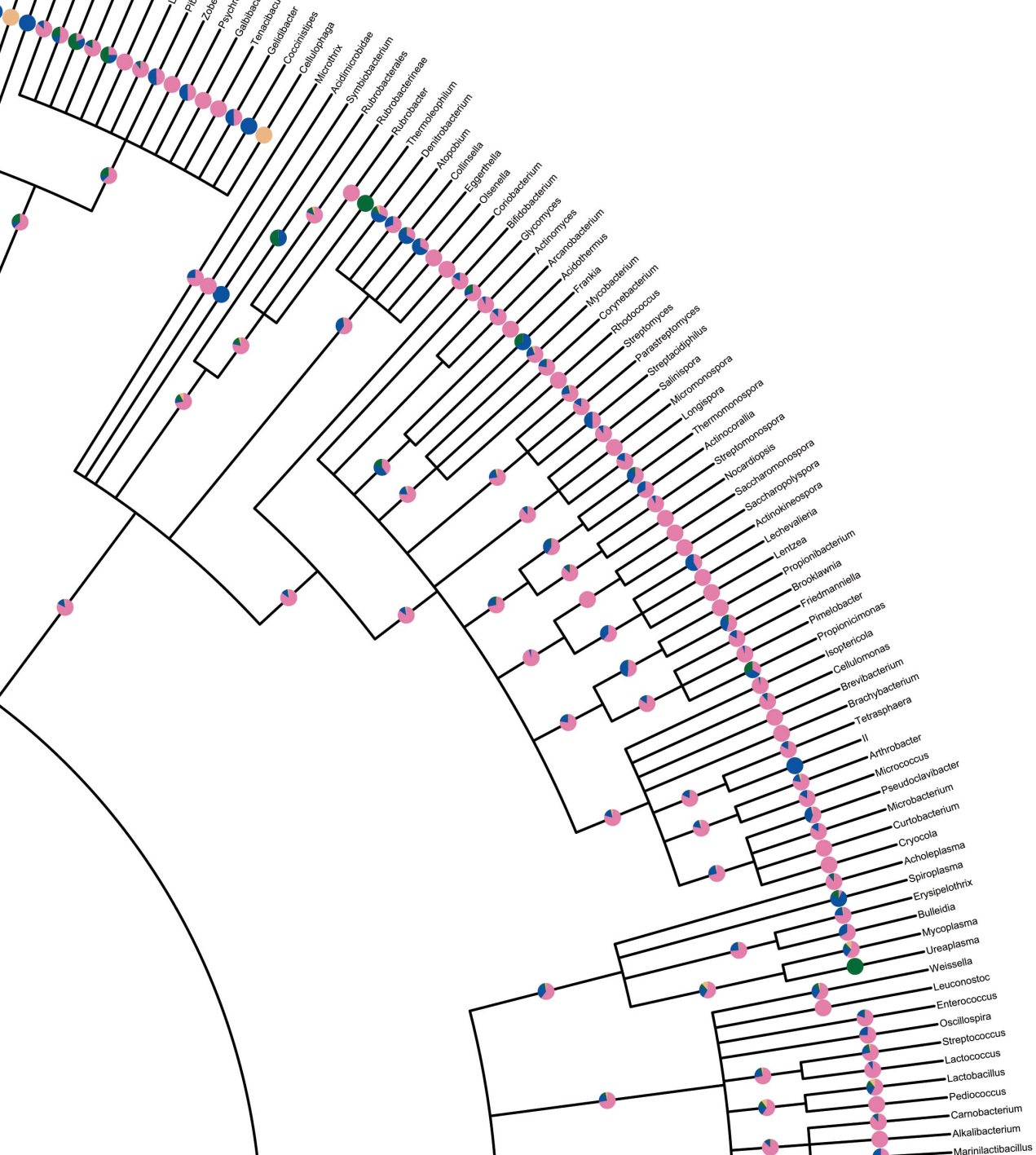


Figure S2 (B) The zoom-in visualization result of the iTOL of comparison four saliva metagenomic dataset.

## Result of MEGAN

The MEGAN software can be used to compare multiple metagenomic samples. But it is not an independent and open source visualization tool, and it cannot be easily imported into other applications or used as an online visualization solution.


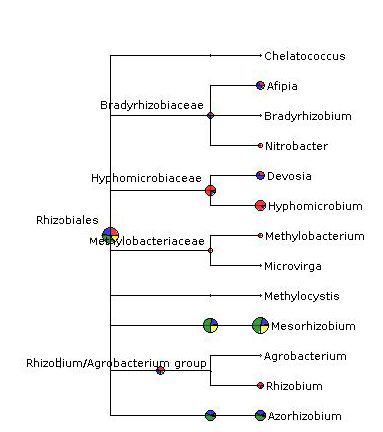


Figure S3 The zoom-in visualization result of the MEGAN of comparison four saliva metagenomic dataset.

## Result of MetaSee

The MetaSee provided a lot of ways to comparison multiple samples. Here we used our Global view for example to analysis these four datasets.

The Global view of MetaSee is a taxonomical hierarchy tree that contains every taxa and their proportion in the sample and shows the whole picture of all samples been compared. In Global view all the taxonomy units in the same level are in the same rank, and the height of each pillar stands for the relative abundance of each sample at this taxonomy unit. If you are interested in the detail information of a certain taxonomy unit, just click a small bar chart, then you can get a pair of pie charts and a pair of bar charts with relative abundance and absolutely abundance. Combined with Taxa view, Global view can show the difference among multiple samples at the global level as well as a taxonomy level.


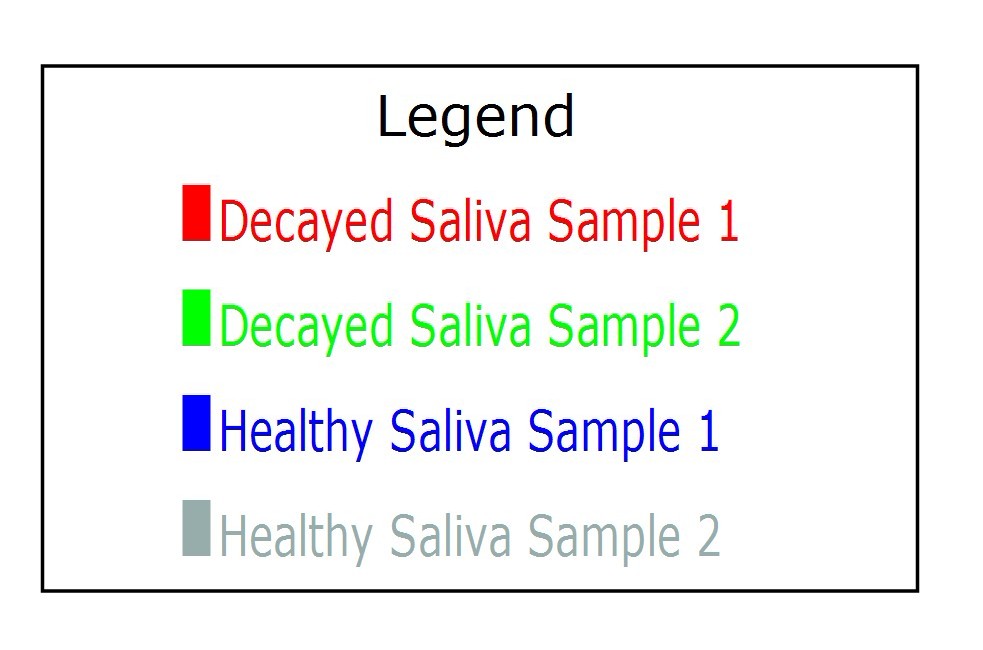


Figure 4S (A) The legend in global view of MetaSee


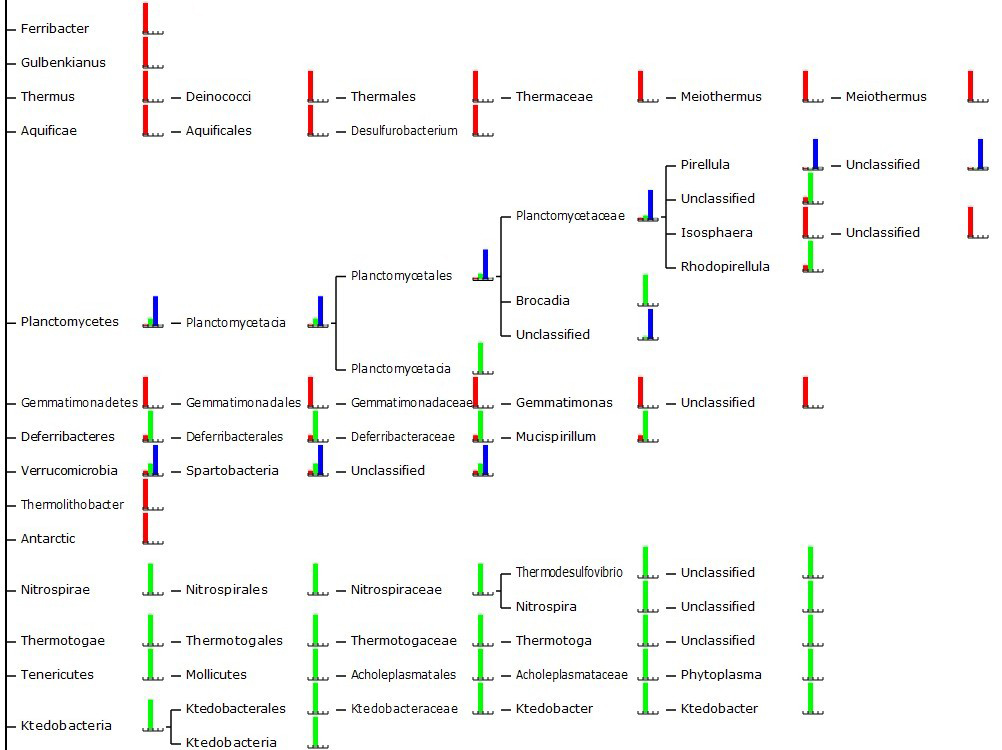


Figure 4S (B) The decayed saliva sample may more diversity in community structure. From this figure we can see that the two saliva samples distribute in some taxonomy with diversity number.


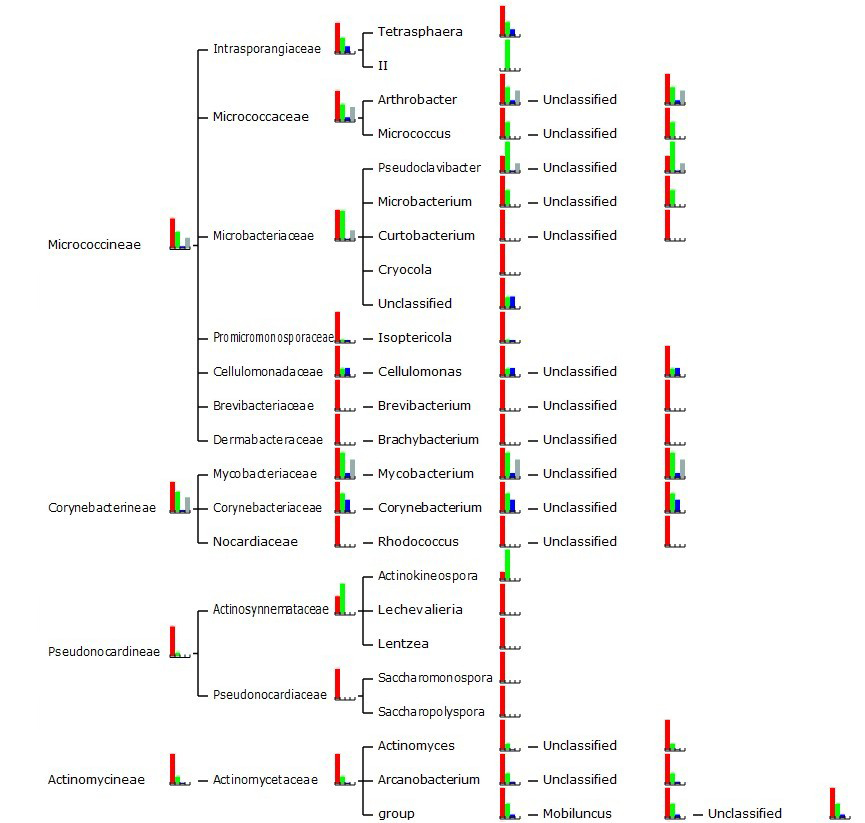


Figure 4S (C) The decayed saliva samples may share a common community structure core. From this figure we can see that the two decayed saliva samples distribute in some taxonomy with similar number but healthy saliva samples almost have no distribution at these taxonomies.


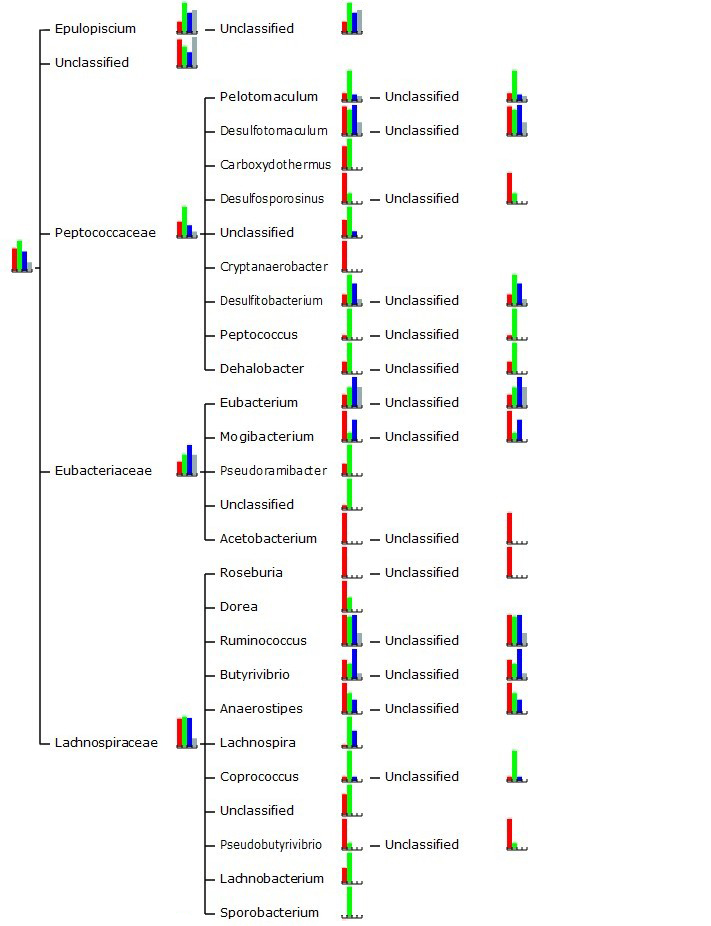


Fig 4S (D) All the saliva samples may share a common community structure core. From this figure we can see that the both two decayed saliva samples and healthy saliva samples distribute in some taxonomy with similar number.

# Appendix

## File used for iTOL

### Tree file

((((((Desulfuromonas),(Pelobacter),(Geobacter,Geoalkalibacter)),((Bdellovibrio,Bacteriovorax),(Bacteriovorax)),((Desulfobacula,Desulfococcus,Desulfobacterium,Desulfobacter),(Desulfoarculus)),(((Myxococcus),(Cystobacter)),((Chondromyces)),Enhygromyxa,((Polyangium))),((Desulfovibrio)),((Thermodesulforhabdus,Desulforhabdus,Syntrophobacter),(Syntrophus)),((Desulfurella))),(((Pseudomonas),(Acinetobacter,Moraxella,Psychrobacter)),((Stenotrophomonas,Panacagrimonas,Dyella,Lysobacter,Nevskia,Xylella,Xanthomonas)),((Vibrio)),((Halomonas,((Portiera)),Chromohalobacter),Marinomonas,Oceanospirillum,Neptunomonas),((Pectobacterium,Citrobacter,Sodalis,Serratia,Escherichia,Buchnera,Rahnella,Buttiauxella,Photorhabdus,(Saintpaul),Klebsiella,Providencia,Pantoea,Arsenophonus,Enterobacter)),((Microbulbifer,Marinobacter,Glaciecola,Alteromonas),(Pseudoalteromonas),(Shewanella),(Colwellia),(Psychromonas)),((Dichelobacter)),((Legionella),(Coxiella,Rickettsiella)),((Halothiobacillus),(Natronocella,Thioalkalivibrio,Alkalilimnicola,Halorhodospira,Aquasalina),(Allochromatium,Chromatium,Thiocapsa,Thiorhodococcus)),((Methylocaldum,Methylobacter,Methylococcus)),symbionts,((Pasteurella,Gallibacterium,Haemophilus,Mannheimia)),((Methylophaga,Thiomicrospira),(Thioploca,Thiothrix,Beggiatoa),(Francisella)),((Succinimonas),(Aeromonas,Zobellella)),Nitrincola,((Acidithiobacillus)),Solimonas,Riesia,Methylonatronum),(((Burkholderia,Ralstonia),(Hydrogenophaga,Variovorax,Rhodoferax,Acidovorax,Diaphorobacter,Comamonas,Polaromonas,Delftia),(Herbaspirillum,Janthinobacterium,Collimonas),Rubrivivax,(Bordetella,Achromobacter,Taylorella),Leptothrix,Thiomonas,Mitsuaria),((Sterolibacterium,Garrityella,Thauera)),((Nitrosospira,Nitrosomonas)),((Neisseria,Iodobacter,Paludimonas,Chromobacterium,Laribacter)),((Methylophilus,Methylobacillus))),(((Sphingomonas,Kaistobacter,Sphingobium,Zymomonas),(Erythrobacter)),((Nitrobacter,Afipia,Bradyrhizobium),(Hyphomicrobium,Devosia),(Mesorhizobium),(Azorhizobium),(Methylobacterium,Microvirga,Roseomonas),(Rhizobium,Liberibacter,(Rhizobium),Agrobacterium),Kaistina,(Methylocystis),(Chelatococcus)),(Rhodocista,Magnetospirillum,Azospirillum),(Stella,Acetobacter)),((Kopriimonas)),(Odyssella,((Rickettsia)),(Anaplasma),Holosporaceae),((Roseinatronobacter,Rhodobacter,Paracoccus),(Maricaulis))),(((Sulfurospirillum,Campylobacter,Arcobacter),(Helicobacter,Sulfurimonas)),Nitratiruptor,Sulfurovum),Magnetococcus),(((Streptococcus,Lactococcus),Weissella,(Carnobacterium,Alkalibacterium,Marinilactibacillus),(Lactobacillus,Pediococcus),(Enterococcus),(Aerococcus,Globicatella,Facklamia),(Oscillospira),Leuconostoc),((Bacillus,Geobacillus,Exiguobacterium,Anoxybacillus,Lysinibacillus,Lentibacillus),(Paenibacillus,Brevibacillus),Gemella,Staphylococcus,(Sulfobacillus,Alicyclobacillus),(Sporolactobacillus),Salinicoccus,(Listeria),(Planifilum),Macrococcus),(((Clostridium,Faecalibacterium,Alkaliphilus,Tepidimicrobium,Geosporobacter,Tindallia,Acetivibrio,Caloramator,Dorea,Gracilibacter),(Peptoniphilus,Sporanaerobacter,Peptostreptococcus,Sedimentibacter,Anaerococcus),(Schwartzia,Megasphaera,Acidaminococcus,Selenomonas,Anaeroglobus,Dialister,Veillonella,Sporomusa,Thermosinus),(Acidaminococcus),Epulopiscium,(Pelotomaculum,Desulfotomaculum,Carboxydothermus,Desulfosporosinus,Cryptanaerobacter,Desulfitobacterium,Peptococcus,Dehalobacter),(Eubacterium,Mogibacterium,Pseudoramibacter,Acetobacterium),(Roseburia,Dorea,Ruminococcus,Butyrivibrio,Anaerostipes,Lachnospira,Coprococcus,Pseudobutyrivibrio,Lachnobacterium,Sporobacterium),Heliobacteriaceae,Natronoanaerobium,(Ruminococcus),(Thermaerobacter),(Syntrophomonas,Thermaerobacter,Syntrophospora,Caldicellulosiruptor),(Oscillibacter)),(((Moorella),Thermoanaerobacter,Thermovenabulum,Thermosediminibacter,Thermoanaerobacterium)),((Halanaerobium)),((Natranaerobius))),(((Erysipelothrix,Bulleidia)),((Acholeplasma)),((Spiroplasma)),(Mycoplasma,Ureaplasma))),(((((Tetrasphaera,II),(Arthrobacter,Micrococcus),(Pseudoclavibacter,Microbacterium,Curtobacterium,Cryocola),(Isoptericola),(Cellulomonas),(Brevibacterium),(Brachybacterium)),((Mycobacterium),(Corynebacterium),(Rhodococcus)),((Actinokineospora,Lechevalieria,Lentzea),(Saccharomonospora,Saccharopolyspora)),((Actinomyces,Arcanobacterium)),((Streptomyces,Parastreptomyces,Streptacidiphilus)),((Friedmanniella,Pimelobacter,Propionicimonas),(Propionibacterium,Brooklawnia)),((Salinispora,Micromonospora,Longispora)),((Glycomyces)),((Thermomonospora,Actinocorallia),(Streptomonospora,Nocardiopsis)),((Acidothermus),(Frankia))),((Bifidobacterium))),((((Denitrobacterium,Atopobium,Collinsella,Eggerthella,Olsenella,Coriobacterium)))),Microthrix,(((Rubrobacterineae,(Rubrobacter),(Thermoleophilum)),Rubrobacterales)),Acidimicrobidae,Symbiobacterium),((((Paraprevotella),(Porphyromonadaceae,Parabacteroides),(Bacteroides),(Alistipes))),(((Polaribacter,Flavobacterium,Capnocytophaga,Chryseobacterium,Empedobacter,Wautersiella,Leptobacterium,Pibocella,Zobellia,Psychroserpens,Galbibacter,Tenacibaculum,Gelidibacter,Coccinistipes,Cellulophaga),(Owenweeksia))),(((Cytophaga,Marinicola,Flexibacter,Hongiella,Sporocytophaga,Arcicella),(Sphingobacterium,Pedobacter,Sphingoterrabacterium),(Lewinella,Saprospira),(Chitinophaga),(Sediminibacterium),(Rhodothermus),(Persicobacter))),((Prevotella),(Bacteroides),(Tannerella,Parabacteroides,Porphyromonas,Dysgonomonas,Barnesiella,Paludibacter),(Alistipes,Ruminofilibacter)),Niastella),(((Leptotrichia,Streptobacillus,Fusobacterium,Ilyobacter),(Cetobacterium)),((Fusobacterium))),(((Holophaga)),(((Solibacter)))),(((Anabaena)),(Synechococcus,Cyanobium),((Prochlorococcus)),(Limnothrix,Leptolyngbya,Microcoleus)),(((Spirochaeta,Treponema),Sphaerochaeta,(Leptospira))),((((Chlorobaculum,(Pelodictyon,Chlorobium))))),(((Roseiflexus,Chloroflexus)),(Dehalococcoides),((Herpetosiphon))),Ferribacter,Gulbenkianus,(((((Meiothermus))))),((Desulfurobacterium)),((((Pirellula,Isosphaera,Rhodopirellula),Brocadia),Planctomycetacia)),(((Gemmatimonas))),(((Mucispirillum))),(Spartobacteria),Thermolithobacter,Antarctic,(((Thermodesulfovibrio,Nitrospira))),(((Thermotoga))),((((Phytoplasma)))),((((Ktedobacter))),Ktedobacteria),Tammella,Fervidomicrobium,(((Thermodesulfatator)))),(((Pyrenomonas)),(((((((Calyptogena))))))),(((((((Polytrichum))),((((Physcomitrella))))),((((((((((Cuscuta)))),(((((Lactuca))))))),((Platanus))))),(((((Angiopteris)))),((((Equisetum)))))))),((((Auxenochlorella,Chlorella))))),((((Palmaria)),((Ceramium)),((Paralemanea)))),(((Lepocinclis,Monomorphina))),((((((Thalassiosira))),(((Chaetoceros))))))),((vectors)),(((((Picrophilus))),(((Archaeoglobus))),(((Methanopyrus))))));

### Dataset file

LABELS,Decayed Saliva Sample 1,Decayed Saliva Sample 2,Healthy Saliva Sample 1,Healthy Saliva Sample 2,

COLORS,#F082B2,#0057AE,#006E28,#F2BA88,

Geobacteraceae|Archaeoglobus,R50,17107,6467,2406,2118

Geobacteraceae|Thermodesulfatator,R1000,16545,6436,2355,2096

Geobacteraceae|Nitratiruptor,R1000,1908,921,1288,1064

Geobacteraceae|Desulfoarculus,R1000,126,174,267,51

Desulfuromonas|Geobacteraceae,R1000,53,106,191,30

Desulfuromonas,R1000,20,12,0,0

Pelobacter,R1000,18,58,161,13

Geobacter|Geoalkalibacter,R1000,15,36,30,17

Geobacter,R1000,14,32,30,17

Geoalkalibacter,R1000,1,4,0,0

Bdellovibrio|Bacteriovoracaceae,R1000,16,22,15,2

Bdellovibrio|Bacteriovorax,R1000,15,21,15,2

Bdellovibrio,R1000,14,18,11,2

Bacteriovorax,R1000,1,3,4,0

Bacteriovoracaceae,R1000,1,1,0,0

Desulfobacula|Desulfoarculus,R1000,7,3,0,0

Desulfobacula|Desulfobacterium,R1000,5,1,0,0

Desulfobacula,R1000,2,0,0,0

Desulfococcus,R1000,1,0,0,0

Desulfobacterium,R1000,2,0,0,0

Desulfobacter,R1000,0,1,0,0

Desulfoarculus,R1000,2,2,0,0

Myxococcus|Polyangium,R1000,22,10,8,4

Myxococcus|Cystobacter,R1000,9,9,7,2

Myxococcus,R1000,9,8,6,2

Cystobacter,R1000,0,1,1,0

Chondromyces,R1000,1,0,0,0

Enhygromyxa,R1000,5,0,0,0

Polyangium,R1000,0,1,1,2

Desulfovibrio,R1000,8,5,1,2

Thermodesulforhabdus|Syntrophus,R1000,1,4,0,0

Thermodesulforhabdus|Syntrophobacter,R1000,1,2,0,0

Thermodesulforhabdus,R1000,1,0,0,0

Desulforhabdus,R1000,0,1,0,0

Syntrophobacter,R1000,0,1,0,0

Syntrophus,R1000,0,2,0,0

Desulfurella,R1000,0,6,3,0

Pseudomonas|Methylonatronum,R1000,957,430,520,421

Pseudomonas|Psychrobacter,R1000,266,187,272,113

Pseudomonas,R1000,246,187,272,77

Moraxellaceae|Acinetobacter,R1000,20,36,0,0

Acinetobacter,R1000,20,4,0,0

Moraxella,R1000,0,0,0,26

Psychrobacter,R1000,0,0,0,6

Stenotrophomonas|Panacagrimonas,R1000,57,21,46,10

Stenotrophomonas,R1000,39,6,15,1

Panacagrimonas,R1000,3,0,0,0

Dyella,R1000,9,1,0,0

Lysobacter,R1000,1,1,22,1

Nevskia,R1000,2,4,9,6

Xylella,R1000,3,0,0,0

Xanthomonas,R1000,0,10,1,0

Vibrio,R1000,87,9,6,17

Halomonas|Neptunomonas,R1000,31,9,16,18

Halomonas|Portiera,R1000,20,1,7,0

Halomonas,R1000,19,1,6,0

Portiera,R1000,1,0,0,0

Chromohalobacter,R1000,0,0,0,1

Marinomonas,R1000,4,1,0,0

Oceanospirillum,R1000,3,7,16,10

Neptunomonas,R1000,4,1,0,0

Pectobacterium|Citrobacter,R1000,159,17,10,24

Pectobacterium,R1000,31,2,1,9

Citrobacter,R1000,52,3,3,1

Sodalis,R1000,6,1,0,0

Serratia,R1000,10,3,6,0

Escherichia,R1000,26,3,1,2

Buchnera,R1000,5,0,0,0

Rahnella,R1000,2,0,0,0

Buttiauxella,R1000,14,0,0,0

Photorhabdus,R1000,1,0,0,0

Salmonella,R1000,8,0,0,0

Saintpaul,R1000,8,0,0,0

Klebsiella,R1000,2,0,0,0

Providencia,R1000,1,0,0,0

Pantoea,R1000,1,0,0,0

Arsenophonus,R1000,0,2,3,0

Enterobacter,R1000,0,0,1,1

Microbulbifer|Psychromonas,R1000,94,27,9,17

Microbulbifer|Glaciecola,R1000,6,2,2,0

Microbulbifer,R1000,3,2,0,0

Marinobacter,R1000,2,0,0,0

Glaciecola,R1000,1,0,0,0

Alteromonas,R1000,0,2,0,0

Pseudoalteromonas,R1000,19,21,7,9

Shewanella,R1000,49,3,2,5

Colwellia,R1000,19,1,1,0

Psychromonas,R1000,1,0,0,0

Dichelobacter,R1000,3,3,0,0

Legionella|Coxiella,R1000,34,26,6,23

Legionella,R1000,12,1,3,0

Coxiella|Rickettsiella,R1000,7,5,3,8

Coxiella,R1000,2,3,0,0

Rickettsiella,R1000,5,5,3,5

Halothiobacillus|Natronocella,R1000,32,13,55,12

Halothiobacillus,R1000,5,0,0,0

Natronocella|Thioalkalivibrio,R1000,18,4,29,11

Natronocella,R1000,4,0,0,0

Thioalkalivibrio,R1000,9,2,0,0

Alkalilimnicola,R1000,4,3,29,8

Halorhodospira,R1000,1,0,0,0

Aquasalina,R1000,0,1,1,0

Allochromatium|Thiorhodococcus,R1000,9,9,26,1

Allochromatium,R1000,2,8,24,0

Chromatium,R1000,6,2,1,0

Thiocapsa,R1000,1,0,0,0

Thiorhodococcus,R1000,0,1,0,0

Methylocaldum|Methylobacter,R1000,6,5,38,15

Methylocaldum,R1000,1,1,1,0

Methylobacter,R1000,4,5,37,14

Methylococcus,R1000,1,0,0,0

symbionts,R1000,2,3,13,0

Pasteurella|Gallibacterium,R1000,47,62,2,101

Pasteurella,R1000,30,46,67,0

Gallibacterium,R1000,3,1,6,0

Haemophilus,R1000,5,13,0,0

Mannheimia,R1000,0,1,2,4

Methylophaga|Francisella,R1000,18,1,1,5

Methylophaga|Thiomicrospira,R1000,10,1,4,0

Methylophaga,R1000,10,3,0,0

Thiomicrospira,R1000,0,0,1,1

Thioploca|Thiothrix,R1000,7,1,0,0

Thioploca,R1000,1,1,0,0

Thiothrix,R1000,2,0,0,0

Beggiatoa,R1000,4,0,0,0

Francisella,R1000,1,1,0,0

Succinimonas|Aeromonas,R1000,44,25,1,11

Succinimonas,R1000,5,1,0,0

Aeromonas|Zobellella,R1000,39,24,1,11

Aeromonas,R1000,34,19,1,5

Zobellella,R1000,5,5,6,0

Nitrincola,R1000,1,0,0,0

Acidithiobacillus,R1000,19,14,0,0

Solimonas,R1000,1,0,0,0

Riesia,R1000,0,4,0,0

Methylonatronum,R1000,0,0,0,6

Ralstonia|Methylophilus,R1000,373,133,305,486

Ralstonia|Mitsuaria,R1000,126,76,212,82

Burkholderia|Ralstonia,R1000,42,51,205,67

Burkholderia,R1000,38,51,205,57

Ralstonia,R1000,4,10,0,0

Hydrogenophaga|Variovorax,R1000,59,13,3,0

Hydrogenophaga,R1000,6,1,1,0

Variovorax,R1000,27,1,1,0

Rhodoferax,R1000,2,0,0,0

Acidovorax,R1000,7,0,0,0

Diaphorobacter,R1000,8,0,0,0

Comamonas,R1000,6,11,1,0

Polaromonas,R1000,1,0,0,0

Delftia,R1000,2,0,0,0

Herbaspirillum|Janthinobacterium,R1000,3,3,4,0

Herbaspirillum,R1000,1,3,0,0

Janthinobacterium,R1000,1,3,1,0

Collimonas,R1000,1,0,0,0

Rubrivivax,R1000,9,3,1,2

Bordetella|Achromobacter,R1000,8,4,5,6

Bordetella,R1000,7,3,5,5

Achromobacter,R1000,1,1,0,0

Taylorella,R1000,0,0,0,1

Leptothrix,R1000,3,2,1,0

Thiomonas,R1000,1,0,0,0

Mitsuaria,R1000,1,0,0,0

Garrityella|Thauera,R1000,29,9,26,37

Sterolibacterium,R1000,13,4,26,13

Garrityella,R1000,1,0,0,0

Thauera,R1000,2,0,0,0

Nitrosospira|Nitrosomonas,R1000,6,6,27,14

Nitrosospira,R1000,3,5,27,8

Nitrosomonas,R1000,3,1,6,0

Neisseria|Laribacter,R1000,188,34,28,334

Neisseria,R1000,163,26,20,317

Iodobacter,R1000,13,8,7,13

Paludimonas,R1000,4,1,4,0

Chromobacterium,R1000,7,0,0,0

Laribacter,R1000,1,0,0,0

Methylophilus|Methylobacillus,R1000,3,1,3,0

Methylophilus,R1000,3,0,0,0

Methylobacillus,R1000,0,1,3,0

Sphingomonas|Maricaulis,R1000,187,122,190,72

Sphingomonas|Erythrobacter,R1000,25,17,60,12

Sphingomonas|Kaistobacter,R1000,21,17,60,11

Sphingomonas,R1000,17,17,53,11

Kaistobacter,R1000,1,7,0,0

Sphingobium,R1000,1,0,0,0

Zymomonas,R1000,2,0,0,0

Erythrobacter,R1000,4,1,0,0

Nitrobacter|Chelatococcus,R1000,45,39,70,30

Nitrobacter|Afipia,R1000,4,3,2,0

Nitrobacter,R1000,2,0,0,0

Afipia,R1000,2,3,1,0

Bradyrhizobium,R1000,0,0,1,0

Hyphomicrobium|Devosia,R1000,18,4,2,0

Hyphomicrobium,R1000,16,2,0,0

Devosia,R1000,2,4,0,0

Mesorhizobium,R1000,3,16,48,23

Azorhizobium,R1000,2,12,17,1

Methylobacterium|Roseomonas,R1000,6,0,0,0

Methylobacterium,R1000,2,0,0,0

Microvirga,R1000,1,0,0,0

Roseomonas,R1000,3,0,0,0

Rhizobium|Agrobacterium,R1000,5,2,2,0

Rhizobium,R1000,3,1,0,0

group,R1000,1,1,1,0

Rhizobium,R1000,1,1,1,0

Agrobacterium,R1000,0,0,0,1

Kaistina,R1000,3,1,0,0

Methylocystis,R1000,1,0,0,0

Chelatococcus,R1000,0,1,0,0

Rhodocista|Acetobacter,R1000,34,16,9,1

Rhodospirillaceae,R1000,28,15,9,1

Rhodocista,R1000,11,11,1,0

Magnetospirillum,R1000,9,0,0,0

Azospirillum,R1000,4,1,0,0

Stella|Acetobacter,R1000,6,1,0,0

Stella,R1000,4,0,0,0

Acetobacter,R1000,2,1,0,0

Kopriimonas,R1000,1,0,0,0

Odyssella|Holosporaceae,R1000,3,1,0,0

Odyssella,R1000,1,0,0,0

Rickettsia,R1000,1,0,0,0

Anaplasma,R1000,1,0,0,0

Holosporaceae,R1000,0,0,1,0

Roseinatronobacter|Maricaulis,R1000,3,14,25,3

Roseinatronobacter|Paracoccus,R1000,3,12,25,3

Roseinatronobacter,R1000,1,9,2,0

Rhodobacter,R1000,1,3,23,2

Paracoccus,R1000,1,1,0,0

Maricaulis,R1000,0,2,0,0

Sulfurospirillum|Sulfurovum,R1000,241,57,3,25

Sulfurospirillum|Sulfurimonas,R1000,233,49,1,22

Sulfurospirillum|Arcobacter,R1000,178,33,19,0

Sulfurospirillum,R1000,10,5,1,0

Campylobacter,R1000,148,27,18,0

Arcobacter,R1000,20,1,0,0

Helicobacter|Sulfurimonas,R1000,49,16,1,2

Helicobacter,R1000,35,16,1,2

Sulfurimonas,R1000,14,0,0,0

Nitratiruptor,R1000,2,2,0,0

Sulfurovum,R1000,6,6,2,3

Magnetococcus,R1000,4,2,3,2

Streptococcus|Ureaplasma,R1000,6206,2819,770,295

Streptococcus|Leuconostoc,R1000,1482,380,123,70

Streptococcus|Lactococcus,R1000,1253,325,112,53

Streptococcus,R1000,1243,325,112,52

Lactococcus,R1000,10,1,0,0

Weissella,R1000,16,8,2,1

Carnobacterium|Marinilactibacillus,R1000,40,4,2,0

Carnobacterium,R1000,37,3,2,0

Alkalibacterium,R1000,1,0,0,0

Marinilactibacillus,R1000,2,1,0,0

Lactobacillus|Pediococcus,R1000,51,17,9,9

Lactobacillus,R1000,48,17,9,9

Pediococcus,R1000,2,0,0,0

Enterococcus,R1000,90,18,4,0

Aerococcus|Facklamia,R1000,7,2,0,0

Aerococcus,R1000,6,1,0,0

Globicatella,R1000,1,0,0,0

Facklamia,R1000,0,1,0,0

Oscillospira,R1000,18,6,0,0

Leuconostoc,R1000,1,0,0,0

Bacillales,R1000,436,295,136,22

Bacillus|Lentibacillus,R1000,245,181,129,10

Bacillus,R1000,219,135,121,8

Geobacillus,R1000,4,5,0,0

Exiguobacterium,R1000,17,37,8,2

Anoxybacillus,R1000,3,2,0,0

Lysinibacillus,R1000,1,2,0,0

Lentibacillus,R1000,1,0,0,0

Paenibacillus|Brevibacillus,R1000,40,74,6,3

Paenibacillus,R1000,23,30,4,3

Brevibacillus,R1000,17,40,2,0

Gemella,R1000,51,6,7,0

Staphylococcus,R1000,28,1,0,0

Sulfobacillus|Alicyclobacillus,R1000,19,12,0,0

Sulfobacillus,R1000,12,10,0,0

Alicyclobacillus,R1000,7,2,0,0

Sporolactobacillus,R1000,8,7,1,0

Salinicoccus,R1000,1,0,0,0

Listeria,R1000,4,2,0,0

Planifilum,R1000,11,9,0,0

Macrococcus,R1000,1,0,0,0

Clostridium|Natranaerobius,R1000,3895,1964,474,189

Clostridium|Oscillibacter,R1000,3796,1916,465,185

Clostridium|Geosporobacter,R1000,1692,738,332,89

Clostridium,R1000,1545,705,330,79

Faecalibacterium,R1000,12,14,0,0

Alkaliphilus,R1000,5,0,0,0

Tepidimicrobium,R1000,1,0,0,0

Geosporobacter,R1000,10,2,1,0

Tindallia,R1000,21,5,4,0

Acetivibrio,R1000,12,3,0,0

Caloramator,R1000,2,1,2,0

Dorea,R1000,9,0,0,0

Gracilibacter,R1000,2,0,0,0

Peptoniphilus|Anaerococcus,R1000,26,18,1,10

Peptoniphilus,R1000,20,9,0,0

Sporanaerobacter,R1000,1,0,0,0

Peptostreptococcus,R1000,5,2,7,0

Sedimentibacter,R1000,0,4,1,3

Anaerococcus,R1000,0,3,0,0

Schwartzia|Thermosinus,R1000,1273,775,16,25

Schwartzia,R1000,305,240,12,5

Megasphaera,R1000,36,24,0,0

Acidaminococcus,R1000,65,105,3,0

Selenomonas,R1000,109,17,1,0

Anaeroglobus,R1000,43,82,1,0

Dialister,R1000,33,49,1,0

Veillonella,R1000,644,228,15,0

Sporomusa,R1000,38,30,1,1

Thermosinus,R1000,0,0,0,1

Acidaminococcus,R1000,27,18,0,0

Epulopiscium,R1000,4,4,1,1

Pelotomaculum|Dehalobacter,R1000,175,131,19,8

Pelotomaculum,R1000,52,75,6,4

Desulfotomaculum,R1000,54,18,8,3

Carboxydothermus,R1000,4,2,0,0

Desulfosporosinus,R1000,25,3,0,0

Cryptanaerobacter,R1000,3,0,0,0

Desulfitobacterium,R1000,14,15,4,1

Peptococcus,R1000,1,3,0,0

Dehalobacter,R1000,1,1,0,0

Eubacterium|Acetobacterium,R1000,132,76,42,25

Eubacterium,R1000,119,72,41,24

Mogibacterium,R1000,10,1,1,0

Pseudoramibacter,R1000,1,1,0,0

Acetobacterium,R1000,1,0,0,0

Roseburia|Sporobacterium,R1000,264,106,38,10

Roseburia,R1000,3,0,0,0

Dorea,R1000,6,1,0,0

Ruminococcus,R1000,121,43,17,6

Butyrivibrio,R1000,81,24,18,3

Anaerostipes,R1000,16,4,1,0

Lachnospira,R1000,1,5,1,0

Coprococcus,R1000,7,19,1,0

Pseudobutyrivibrio,R1000,14,1,0,0

Lachnobacterium,R1000,4,3,0,0

Sporobacterium,R1000,0,1,0,0

Heliobacteriaceae,R1000,12,0,0,0

Natronoanaerobium,R1000,3,4,0,0

Ruminococcus,R1000,9,0,0,0

Thermaerobacter,R1000,9,5,2,0

Syntrophomonas|Caldicellulosiruptor,R1000,13,2,5,1

Syntrophomonas,R1000,6,2,5,1

Thermaerobacter,R1000,1,0,0,0

Syntrophospora,R1000,5,0,0,0

Caldicellulosiruptor,R1000,1,0,0,0

Oscillibacter,R1000,2,1,0,0

Thermoanaerobacter|Thermoanaerobacterium,R1000,90,46,8,1

Thermoanaerobacter,R1000,17,14,5,0

Thermovenabulum,R1000,15,13,2,0

Thermosediminibacter,R1000,1,0,0,0

Thermoanaerobacterium,R1000,1,0,0,0

Halanaerobium,R1000,8,2,3,0

Natranaerobius,R1000,0,0,1,0

Erysipelothrix|Ureaplasma,R1000,256,137,22,10

Erysipelothrix|Bulleidia,R1000,147,46,3,3

Erysipelothrix,R1000,125,35,3,3

Bulleidia,R1000,22,11,0,0

Acholeplasma,R1000,16,1,1,0

Spiroplasma,R1000,3,29,7,0

Mycoplasma|Ureaplasma,R1000,35,14,4,7

Mycoplasma,R1000,35,14,3,7

Ureaplasma,R1000,0,0,1,0

Tetrasphaera|Symbiobacterium,R1000,3973,697,30,97

Tetrasphaera|Bifidobacterium,R1000,3759,569,19,90

Tetrasphaera|Frankia,R1000,3647,548,19,89

Tetrasphaera|Brachybacterium,R1000,870,180,9,39

Tetrasphaera|II,R1000,64,12,2,0

Tetrasphaera,R1000,64,11,2,0

II,R1000,0,1,0,0

Arthrobacter|Micrococcus,R1000,468,97,8,27

Arthrobacter,R1000,463,97,8,26

Micrococcus,R1000,5,1,0,0

Pseudoclavibacter|Cryocola,R1000,172,63,1,7

Pseudoclavibacter,R1000,75,53,1,5

Microbacterium,R1000,25,5,0,0

Curtobacterium,R1000,7,0,0,0

Cryocola,R1000,28,0,0,0

Isoptericola,R1000,107,3,1,0

Cellulomonas,R1000,54,5,2,0

Brevibacterium,R1000,4,0,0,0

Brachybacterium,R1000,1,0,0,0

Mycobacterium|Corynebacterium,R1000,408,103,4,25

Mycobacterium,R1000,170,54,4,13

Corynebacterium,R1000,209,49,12,0

Rhodococcus,R1000,29,0,0,0

Actinokineospora|Saccharopolyspora,R1000,151,7,0,0

Actinokineospora|Lentzea,R1000,11,7,0,0

Actinokineospora,R1000,5,7,0,0

Lechevalieria,R1000,5,0,0,0

Lentzea,R1000,1,0,0,0

Saccharomonospora|Saccharopolyspora,R1000,140,0,0,0

Saccharomonospora,R1000,127,0,0,0

Saccharopolyspora,R1000,13,0,0,0

Actinomyces|Mobiluncus,R1000,1883,171,11,0

Actinomyces,R1000,1477,112,6,0

Arcanobacterium,R1000,286,38,3,0

Mobiluncus,R1000,120,21,2,0

Streptomyces|Streptacidiphilus,R1000,139,40,5,7

Streptomyces,R1000,133,39,5,6

Parastreptomyces,R1000,5,1,0,0

Streptacidiphilus,R1000,1,1,0,0

Friedmanniella|Brooklawnia,R1000,114,31,2,0

Friedmanniella|Propionicimonas,R1000,99,18,1,0

Friedmanniella,R1000,79,16,0,0

Pimelobacter,R1000,19,1,0,0

Propionicimonas,R1000,1,1,1,0

Propionibacterium|Brooklawnia,R1000,15,13,1,0

Propionibacterium,R1000,4,0,0,0

Brooklawnia,R1000,6,4,1,0

Salinispora|Longispora,R1000,48,5,1,0

Salinispora,R1000,43,5,0,0

Micromonospora,R1000,1,0,0,0

Longispora,R1000,4,1,0,0

Glycomyces,R1000,9,2,2,0

Thermomonospora|Nocardiopsis,R1000,23,7,2,0

Thermomonospora|Actinocorallia,R1000,10,6,1,0

Thermomonospora,R1000,8,5,1,0

Actinocorallia,R1000,2,1,0,0

Streptomonospora|Nocardiopsis,R1000,13,1,1,0

Streptomonospora,R1000,12,1,0,0

Nocardiopsis,R1000,1,0,0,0

Acidothermus|Frankia,R1000,2,2,1,0

Acidothermus,R1000,2,0,0,0

Frankia,R1000,0,2,1,0

Bifidobacterium,R1000,112,21,1,0

Denitrobacterium|Coriobacterium,R1000,168,101,7,4

Denitrobacterium,R1000,16,25,6,3

Atopobium,R1000,142,61,1,0

Collinsella,R1000,7,13,1,0

Eggerthella,R1000,1,2,0,0

Olsenella,R1000,1,0,0,0

Coriobacterium,R1000,1,0,0,0

Microthrix,R1000,3,1,0,0

Rubrobacterineae|Rubrobacterales,R1000,18,2,3,2

Rubrobacterineae|Thermoleophilum,R1000,18,1,3,1

Rubrobacterineae,R1000,17,1,2,1

Rubrobacter,R1000,1,0,0,0

Thermoleophilum,R1000,0,0,1,0

Rubrobacterales,R1000,0,1,1,0

Acidimicrobidae,R1000,6,0,0,0

Symbiobacterium,R1000,0,7,0,0

Paraprevotella|Niastella,R1000,2026,1370,104,389

Paraprevotella|Alistipes,R1000,405,337,31,82

Paraprevotella,R1000,14,8,20,0

Porphyromonadaceae|Parabacteroides,R1000,116,320,31,28

Porphyromonadaceae,R1000,114,320,31,28

Parabacteroides,R1000,2,0,0,0

Bacteroides,R1000,58,9,25,0

Alistipes,R1000,2,0,0,0

Polaribacter|Owenweeksia,R1000,108,19,45,0

Polaribacter|Cellulophaga,R1000,108,18,45,0

Polaribacter,R1000,13,2,0,0

Flavobacterium,R1000,11,4,6,0

Capnocytophaga,R1000,7,8,22,0

Chryseobacterium,R1000,19,2,2,0

Empedobacter,R1000,1,1,2,0

Wautersiella,R1000,9,0,0,0

Leptobacterium,R1000,17,1,1,0

Pibocella,R1000,3,3,0,0

Zobellia,R1000,1,0,0,0

Psychroserpens,R1000,1,1,0,0

Galbibacter,R1000,1,0,0,0

Tenacibaculum,R1000,1,0,0,0

Gelidibacter,R1000,2,2,0,0

Coccinistipes,R1000,0,1,0,0

Cellulophaga,R1000,0,0,0,1

Owenweeksia,R1000,0,1,0,0

Cytophaga|Persicobacter,R1000,42,54,33,18

Cytophaga|Arcicella,R1000,21,34,29,15

Cytophaga,R1000,14,9,3,0

Marinicola,R1000,2,0,0,0

Flexibacter,R1000,3,9,27,9

Hongiella,R1000,1,0,0,0

Sporocytophaga,R1000,1,14,2,1

Arcicella,R1000,0,0,0,1

Sphingobacterium|Sphingoterrabacterium,R1000,5,8,2,2

Sphingobacterium,R1000,3,2,2,0

Pedobacter,R1000,2,4,2,0

Sphingoterrabacterium,R1000,0,2,0,0

Lewinella|Saprospira,R1000,12,6,1,0

Lewinella,R1000,7,2,0,0

Saprospira,R1000,5,0,0,0

Chitinophaga,R1000,1,6,1,0

Sediminibacterium,R1000,1,0,0,0

Rhodothermus,R1000,1,0,0,0

Persicobacter,R1000,0,0,1,0

Prevotella|Ruminofilibacter,R1000,1442,914,37,231

Prevotella,R1000,815,397,18,78

Bacteroides,R1000,489,402,98,0

Tannerella|Paludibacter,R1000,55,24,1,42

Tannerella,R1000,17,8,1,14

Parabacteroides,R1000,14,4,4,0

Porphyromonas,R1000,11,6,21,0

Dysgonomonas,R1000,9,6,0,0

Barnesiella,R1000,2,0,0,0

Paludibacter,R1000,2,3,0,0

Alistipes|Ruminofilibacter,R1000,11,55,17,11

Alistipes,R1000,8,48,17,8

Ruminofilibacter,R1000,2,7,2,0

Niastella,R1000,1,0,0,0

Leptotrichia|Fusobacterium,R1000,752,139,10,64

Leptotrichia|Cetobacterium,R1000,709,102,2,46

Leptotrichia|Ilyobacter,R1000,708,102,2,45

Leptotrichia,R1000,392,34,14,0

Streptobacillus,R1000,86,18,2,1

Fusobacterium,R1000,106,24,19,0

Ilyobacter,R1000,124,26,11,0

Cetobacterium,R1000,1,1,0,0

Fusobacterium,R1000,43,37,8,18

Holophaga|Solibacter,R1000,59,36,10,6

Holophaga,R1000,4,1,0,0

Solibacter,R1000,2,0,0,0

Anabaena|Microcoleus,R1000,36,6,2,0

Anabaena,R1000,3,1,0,0

Synechococcus|Cyanobium,R1000,18,0,0,0

Synechococcus,R1000,8,0,0,0

Cyanobium,R1000,10,0,0,0

Prochlorococcus,R1000,3,2,1,0

Limnothrix|Microcoleus,R1000,11,2,0,0

Limnothrix,R1000,9,0,0,0

Leptolyngbya,R1000,1,0,0,0

Microcoleus,R1000,0,2,0,0

Spirochaeta|Leptospira,R1000,106,11,4,8

Spirochaeta|Treponema,R1000,99,10,4,7

Spirochaeta,R1000,16,3,3,2

Treponema,R1000,80,7,1,4

Sphaerochaeta,R1000,4,0,0,0

Leptospira,R1000,3,1,1,0

Chlorobaculum|Chlorobium,R1000,3,2,0,0

Chlorobaculum,R1000,1,0,0,0

Pelodictyon|Chlorobium,R1000,2,2,0,0

Pelodictyon,R1000,1,1,0,0

Chlorobium,R1000,1,1,0,0

Roseiflexus|Herpetosiphon,R1000,40,23,9,3

Roseiflexus|Chloroflexus,R1000,24,11,7,1

Roseiflexus,R1000,7,2,0,0

Chloroflexus,R1000,17,9,7,1

Dehalococcoides,R1000,4,6,1,0

Herpetosiphon,R1000,1,0,0,0

Ferribacter,R1000,3,0,0,0

Gulbenkianus,R1000,15,0,0,0

Meiothermus,R1000,1,0,0,0

Desulfurobacterium,R1000,1,0,0,0

Pirellula|Planctomycetacia,R1000,7,12,18,1

Pirellula|Brocadia,R1000,7,9,18,1

Pirellula|Rhodopirellula,R1000,7,5,13,1

Pirellula,R1000,4,1,13,1

Isosphaera,R1000,1,0,0,0

Rhodopirellula,R1000,1,2,0,0

Brocadia,R1000,0,3,0,0

Planctomycetacia,R1000,0,3,0,0

Gemmatimonas,R1000,1,0,0,0

Mucispirillum,R1000,1,2,0,0

Spartobacteria,R1000,1,1,1,0

Thermolithobacter,R1000,1,0,0,0

Antarctic,R1000,1,0,0,0

Thermodesulfovibrio|Nitrospira,R1000,0,3,0,0

Thermodesulfovibrio,R1000,0,1,0,0

Nitrospira,R1000,0,2,0,0

Thermotoga,R1000,0,1,0,0

Phytoplasma,R1000,0,1,0,0

Ktedobacter|Ktedobacteria,R1000,0,9,0,0

Ktedobacter,R1000,0,7,0,0

Ktedobacteria,R1000,0,2,0,0

Tammella,R1000,0,1,1,0

Fervidomicrobium,R1000,0,0,1,0

Thermodesulfatator,R1000,0,0,1,0

Pyrenomonas|Chaetoceros,R1000,89,30,51,21

Pyrenomonas,R1000,1,0,0,0

Calyptogena,R1000,22,11,47,18

Polytrichum|Chlorella,R1000,56,1,0,0

Polytrichum|Equisetum,R1000,30,0,0,0

Polytrichum|Physcomitrella,R1000,5,0,0,0

Polytrichopsida,R1000,4,0,0,0

Polytrichum,R1000,4,0,0,0

Physcomitrella,R1000,1,0,0,0

lamiids|Equisetum,R1000,25,0,0,0

Cuscuta|Lactuca,R1000,13,0,0,0

Cuscuta,R1000,6,0,0,0

Lactuca,R1000,4,0,0,0

Platanus,R1000,3,0,0,0

Angiopteris|Equisetum,R1000,12,0,0,0

Angiopteris,R1000,7,0,0,0

Equisetum,R1000,5,0,0,0

Auxenochlorella|Chlorella,R1000,26,1,0,0

Auxenochlorella,R1000,24,1,0,0

Chlorella,R1000,2,0,0,0

Palmaria|Ceramium,R1000,7,17,3,0

Palmaria,R1000,3,17,3,0

Ceramium,R1000,3,0,0,0

Paralemanea,R1000,1,0,0,0

Lepocinclis|Monomorphina,R1000,1,1,0,0

Lepocinclis,R1000,1,0,0,0

Monomorphina,R1000,0,0,1,0

Thalassiosira|Chaetoceros,R1000,2,1,3,0

Thalassiosira,R1000,1,1,3,0

Chaetoceros,R1000,1,0,0,0

vectors,R1000,457,0,0,0

Picrophilus|Methanopyrus,R1000,16,1,0,0

Picrophilus,R1000,11,0,0,0

Archaeoglobus,R1000,5,0,0,0

Methanopyrus,R1000,0,0,0,1

## File used for MEGAN

#Datasets Decayed Saliva Sample 1 Decayed Saliva Sample 2 Healthy Saliva Sample 1 Healthy Saliva Sample 2

root; 17107 6467 2406 2118

root;Bacteria; 16545 6436 2355 2096

root;Bacteria;Proteobacteria; 1908 921 1288 1064

root;Bacteria;Proteobacteria;Deltaproteobacteria; 126 174 267 51

root;Bacteria;Proteobacteria;Deltaproteobacteria;Desulfuromonadales; 53 106 191 30

root;Bacteria;Proteobacteria;Deltaproteobacteria;Desulfuromonadales;Desulfuromonadaceae; 20 12 0 0

root;Bacteria;Proteobacteria;Deltaproteobacteria;Desulfuromonadales;Desulfuromonadaceae;Desulfuromonas; 20 12 0 0

root;Bacteria;Proteobacteria;Deltaproteobacteria;Desulfuromonadales;Desulfuromonadaceae;Desulfuromonas;Unclassified; 20 12 0 0

root;Bacteria;Proteobacteria;Deltaproteobacteria;Desulfuromonadales;Pelobacteraceae; 18 58 161 13

root;Bacteria;Proteobacteria;Deltaproteobacteria;Desulfuromonadales;Pelobacteraceae;Pelobacter; 18 58 161 13

root;Bacteria;Proteobacteria;Deltaproteobacteria;Desulfuromonadales;Pelobacteraceae;Pelobacter;Unclassified; 18 58 161 13

root;Bacteria;Proteobacteria;Deltaproteobacteria;Desulfuromonadales;Geobacteraceae; 15 36 30 17

root;Bacteria;Proteobacteria;Deltaproteobacteria;Desulfuromonadales;Geobacteraceae;Geobacter; 14 32 30 17

root;Bacteria;Proteobacteria;Deltaproteobacteria;Desulfuromonadales;Geobacteraceae;Geobacter;Unclassified; 14 32 30 17

root;Bacteria;Proteobacteria;Deltaproteobacteria;Desulfuromonadales;Geobacteraceae;Geoalkalibacter; 1 4 0 0

root;Bacteria;Proteobacteria;Deltaproteobacteria;Bdellovibrionales; 16 22 15 2

root;Bacteria;Proteobacteria;Deltaproteobacteria;Bdellovibrionales;Bdellovibrionaceae; 15 21 15 2

root;Bacteria;Proteobacteria;Deltaproteobacteria;Bdellovibrionales;Bdellovibrionaceae;Bdellovibrio; 14 18 11 2

root;Bacteria;Proteobacteria;Deltaproteobacteria;Bdellovibrionales;Bdellovibrionaceae;Bdellovibrio;Unclassified; 14 18 11 2

root;Bacteria;Proteobacteria;Deltaproteobacteria;Bdellovibrionales;Bdellovibrionaceae;Bacteriovorax; 1 3 4 0

root;Bacteria;Proteobacteria;Deltaproteobacteria;Bdellovibrionales;Bacteriovoracaceae; 1 1 0 0

root;Bacteria;Proteobacteria;Deltaproteobacteria;Bdellovibrionales;Bacteriovoracaceae;Bacteriovorax; 1 1 0 0

root;Bacteria;Proteobacteria;Deltaproteobacteria;Bdellovibrionales;Bacteriovoracaceae;Bacteriovorax;Unclassified; 1 1 0 0

root;Bacteria;Proteobacteria;Deltaproteobacteria;Unclassified; 19 18 49 13

root;Bacteria;Proteobacteria;Deltaproteobacteria;Desulfobacterales; 7 3 0 0

root;Bacteria;Proteobacteria;Deltaproteobacteria;Desulfobacterales;Desulfobacteraceae; 5 1 0 0

root;Bacteria;Proteobacteria;Deltaproteobacteria;Desulfobacterales;Desulfobacteraceae;Desulfobacula; 2 0 0 0

root;Bacteria;Proteobacteria;Deltaproteobacteria;Desulfobacterales;Desulfobacteraceae;Desulfobacula;Unclassified; 2 0 0 0

root;Bacteria;Proteobacteria;Deltaproteobacteria;Desulfobacterales;Desulfobacteraceae;Desulfococcus; 1 0 0 0

root;Bacteria;Proteobacteria;Deltaproteobacteria;Desulfobacterales;Desulfobacteraceae;Desulfobacterium; 2 0 0 0

root;Bacteria;Proteobacteria;Deltaproteobacteria;Desulfobacterales;Desulfobacteraceae;Desulfobacterium;Unclassified; 2 0 0 0

root;Bacteria;Proteobacteria;Deltaproteobacteria;Desulfobacterales;Desulfobacteraceae;Desulfobacter; 0 1 0 0

root;Bacteria;Proteobacteria;Deltaproteobacteria;Desulfobacterales;Desulfobacteraceae;Desulfobacter;Unclassified; 0 1 0 0

root;Bacteria;Proteobacteria;Deltaproteobacteria;Desulfobacterales;Desulfoarculaceae; 2 2 0 0

root;Bacteria;Proteobacteria;Deltaproteobacteria;Desulfobacterales;Desulfoarculaceae;Desulfoarculus; 2 2 0 0

root;Bacteria;Proteobacteria;Deltaproteobacteria;Myxococcales; 22 10 8 4

root;Bacteria;Proteobacteria;Deltaproteobacteria;Myxococcales;Cystobacterineae; 9 9 7 2

root;Bacteria;Proteobacteria;Deltaproteobacteria;Myxococcales;Cystobacterineae;Myxococcaceae; 9 8 6 2

root;Bacteria;Proteobacteria;Deltaproteobacteria;Myxococcales;Cystobacterineae;Myxococcaceae;Myxococcus; 9 8 6 2

root;Bacteria;Proteobacteria;Deltaproteobacteria;Myxococcales;Cystobacterineae;Cystobacteraceae; 0 1 1 0

root;Bacteria;Proteobacteria;Deltaproteobacteria;Myxococcales;Cystobacterineae;Cystobacteraceae;Cystobacter; 0 1 1 0

root;Bacteria;Proteobacteria;Deltaproteobacteria;Myxococcales;Cystobacterineae;Cystobacteraceae;Cystobacter;Unclassified; 0 1 1 0

root;Bacteria;Proteobacteria;Deltaproteobacteria;Myxococcales;Unclassified; 7 0 0 0

root;Bacteria;Proteobacteria;Deltaproteobacteria;Myxococcales;Sorangiineae; 1 0 0 0

root;Bacteria;Proteobacteria;Deltaproteobacteria;Myxococcales;Sorangiineae;Polyangiaceae; 1 0 0 0

root;Bacteria;Proteobacteria;Deltaproteobacteria;Myxococcales;Sorangiineae;Polyangiaceae;Chondromyces; 1 0 0 0

root;Bacteria;Proteobacteria;Deltaproteobacteria;Myxococcales;Enhygromyxa; 5 0 0 0

root;Bacteria;Proteobacteria;Deltaproteobacteria;Myxococcales;Enhygromyxa;Unclassified; 5 0 0 0

root;Bacteria;Proteobacteria;Deltaproteobacteria;Myxococcales;Sorangineae; 0 1 1 2

root;Bacteria;Proteobacteria;Deltaproteobacteria;Myxococcales;Sorangineae;Polyangiaceae; 0 1 1 2

root;Bacteria;Proteobacteria;Deltaproteobacteria;Myxococcales;Sorangineae;Polyangiaceae;Polyangium; 0 1 1 2

root;Bacteria;Proteobacteria;Deltaproteobacteria;Desulfovibrionales; 8 5 1 2

root;Bacteria;Proteobacteria;Deltaproteobacteria;Desulfovibrionales;Desulfovibrionaceae; 8 5 1 2

root;Bacteria;Proteobacteria;Deltaproteobacteria;Desulfovibrionales;Desulfovibrionaceae;Desulfovibrio; 8 5 1 2

root;Bacteria;Proteobacteria;Deltaproteobacteria;Desulfovibrionales;Desulfovibrionaceae;Desulfovibrio;Unclassified; 8 5 1 2

root;Bacteria;Proteobacteria;Deltaproteobacteria;Syntrophobacterales; 1 4 0 0

root;Bacteria;Proteobacteria;Deltaproteobacteria;Syntrophobacterales;Syntrophobacteraceae; 1 2 0 0

root;Bacteria;Proteobacteria;Deltaproteobacteria;Syntrophobacterales;Syntrophobacteraceae;Thermodesulforhabdus; 1 0 0 0

root;Bacteria;Proteobacteria;Deltaproteobacteria;Syntrophobacterales;Syntrophobacteraceae;Desulforhabdus; 0 1 0 0

root;Bacteria;Proteobacteria;Deltaproteobacteria;Syntrophobacterales;Syntrophobacteraceae;Syntrophobacter; 0 1 0 0

root;Bacteria;Proteobacteria;Deltaproteobacteria;Syntrophobacterales;Syntrophobacteraceae;Syntrophobacter;Unclassified; 0 1 0 0

root;Bacteria;Proteobacteria;Deltaproteobacteria;Syntrophobacterales;Syntrophaceae; 0 2 0 0

root;Bacteria;Proteobacteria;Deltaproteobacteria;Syntrophobacterales;Syntrophaceae;Syntrophus; 0 2 0 0

root;Bacteria;Proteobacteria;Deltaproteobacteria;Syntrophobacterales;Syntrophaceae;Syntrophus;Unclassified; 0 2 0 0

root;Bacteria;Proteobacteria;Deltaproteobacteria;Desulfurellales; 0 6 3 0

root;Bacteria;Proteobacteria;Deltaproteobacteria;Desulfurellales;Desulfurellaceae; 0 6 3 0

root;Bacteria;Proteobacteria;Deltaproteobacteria;Desulfurellales;Desulfurellaceae;Desulfurella; 0 6 3 0

root;Bacteria;Proteobacteria;Deltaproteobacteria;Desulfurellales;Desulfurellaceae;Desulfurella;Unclassified; 0 6 3 0

root;Bacteria;Proteobacteria;Gammaproteobacteria; 957 430 520 421

root;Bacteria;Proteobacteria;Gammaproteobacteria;Pseudomonadales; 266 187 272 113

root;Bacteria;Proteobacteria;Gammaproteobacteria;Pseudomonadales;Pseudomonadaceae; 246 187 272 77

root;Bacteria;Proteobacteria;Gammaproteobacteria;Pseudomonadales;Pseudomonadaceae;Pseudomonas; 246 187 272 77

root;Bacteria;Proteobacteria;Gammaproteobacteria;Pseudomonadales;Pseudomonadaceae;Pseudomonas;Unclassified; 246 187 272 77

root;Bacteria;Proteobacteria;Gammaproteobacteria;Pseudomonadales;Moraxellaceae; 20 36 0 0

root;Bacteria;Proteobacteria;Gammaproteobacteria;Pseudomonadales;Moraxellaceae;Acinetobacter; 20 4 0 0

root;Bacteria;Proteobacteria;Gammaproteobacteria;Pseudomonadales;Moraxellaceae;Acinetobacter;Unclassified; 20 4 0 0

root;Bacteria;Proteobacteria;Gammaproteobacteria;Pseudomonadales;Moraxellaceae;Moraxella; 0 0 0 26

root;Bacteria;Proteobacteria;Gammaproteobacteria;Pseudomonadales;Moraxellaceae;Moraxella;Unclassified; 0 0 0 26

root;Bacteria;Proteobacteria;Gammaproteobacteria;Pseudomonadales;Moraxellaceae;Psychrobacter; 0 0 0 6

root;Bacteria;Proteobacteria;Gammaproteobacteria;Pseudomonadales;Moraxellaceae;Psychrobacter;Unclassified; 0 0 0 6

root;Bacteria;Proteobacteria;Gammaproteobacteria;Unclassified; 56 7 58 33

root;Bacteria;Proteobacteria;Gammaproteobacteria;Xanthomonadales; 57 21 46 10

root;Bacteria;Proteobacteria;Gammaproteobacteria;Xanthomonadales;Xanthomonadaceae; 57 21 46 10

root;Bacteria;Proteobacteria;Gammaproteobacteria;Xanthomonadales;Xanthomonadaceae;Stenotrophomonas; 39 6 15 1

root;Bacteria;Proteobacteria;Gammaproteobacteria;Xanthomonadales;Xanthomonadaceae;Stenotrophomonas;Unclassified; 39 6 15 1

root;Bacteria;Proteobacteria;Gammaproteobacteria;Xanthomonadales;Xanthomonadaceae;Panacagrimonas; 3 0 0 0

root;Bacteria;Proteobacteria;Gammaproteobacteria;Xanthomonadales;Xanthomonadaceae;Dyella; 9 1 0 0

root;Bacteria;Proteobacteria;Gammaproteobacteria;Xanthomonadales;Xanthomonadaceae;Dyella;Unclassified; 9 1 0 0

root;Bacteria;Proteobacteria;Gammaproteobacteria;Xanthomonadales;Xanthomonadaceae;Lysobacter; 1 1 22 1

root;Bacteria;Proteobacteria;Gammaproteobacteria;Xanthomonadales;Xanthomonadaceae;Lysobacter;Unclassified; 1 1 22 1

root;Bacteria;Proteobacteria;Gammaproteobacteria;Xanthomonadales;Xanthomonadaceae;Nevskia; 2 4 9 6

root;Bacteria;Proteobacteria;Gammaproteobacteria;Xanthomonadales;Xanthomonadaceae;Xylella; 3 0 0 0

root;Bacteria;Proteobacteria;Gammaproteobacteria;Xanthomonadales;Xanthomonadaceae;Xanthomonas; 0 10 1 0

root;Bacteria;Proteobacteria;Gammaproteobacteria;Xanthomonadales;Xanthomonadaceae;Xanthomonas;Unclassified; 0 10 1 0

root;Bacteria;Proteobacteria;Gammaproteobacteria;Vibrionales; 87 9 6 17

root;Bacteria;Proteobacteria;Gammaproteobacteria;Vibrionales;Vibrionaceae; 87 9 6 17

root;Bacteria;Proteobacteria;Gammaproteobacteria;Vibrionales;Vibrionaceae;Vibrio; 87 9 6 17

root;Bacteria;Proteobacteria;Gammaproteobacteria;Vibrionales;Vibrionaceae;Vibrio;Unclassified; 87 9 6 17

root;Bacteria;Proteobacteria;Gammaproteobacteria;Oceanospirillales; 31 9 16 18

root;Bacteria;Proteobacteria;Gammaproteobacteria;Oceanospirillales;Halomonadaceae; 20 1 7 0

root;Bacteria;Proteobacteria;Gammaproteobacteria;Oceanospirillales;Halomonadaceae;Halomonas; 19 1 6 0

root;Bacteria;Proteobacteria;Gammaproteobacteria;Oceanospirillales;Halomonadaceae;Halomonas;Unclassified; 19 1 6 0

root;Bacteria;Proteobacteria;Gammaproteobacteria;Oceanospirillales;Halomonadaceae;group; 1 0 0 0

root;Bacteria;Proteobacteria;Gammaproteobacteria;Oceanospirillales;Halomonadaceae;group;endosymbionts; 1 0 0 0

root;Bacteria;Proteobacteria;Gammaproteobacteria;Oceanospirillales;Halomonadaceae;group;endosymbionts;Portiera; 1 0 0 0

root;Bacteria;Proteobacteria;Gammaproteobacteria;Oceanospirillales;Halomonadaceae;Chromohalobacter; 0 0 0 1

root;Bacteria;Proteobacteria;Gammaproteobacteria;Oceanospirillales;Halomonadaceae;Chromohalobacter;Unclassified; 0 0 0 1

root;Bacteria;Proteobacteria;Gammaproteobacteria;Oceanospirillales;Marinomonas; 4 1 0 0

root;Bacteria;Proteobacteria;Gammaproteobacteria;Oceanospirillales;Marinomonas;Unclassified; 4 1 0 0

root;Bacteria;Proteobacteria;Gammaproteobacteria;Oceanospirillales;Oceanospirillum; 3 7 16 10

root;Bacteria;Proteobacteria;Gammaproteobacteria;Oceanospirillales;Oceanospirillum;Unclassified; 3 7 16 10

root;Bacteria;Proteobacteria;Gammaproteobacteria;Oceanospirillales;Neptunomonas; 4 1 0 0

root;Bacteria;Proteobacteria;Gammaproteobacteria;Oceanospirillales;Neptunomonas;Unclassified; 4 1 0 0

root;Bacteria;Proteobacteria;Gammaproteobacteria;Enterobacteriales; 159 17 10 24

root;Bacteria;Proteobacteria;Gammaproteobacteria;Enterobacteriales;Enterobacteriaceae; 159 17 10 24

root;Bacteria;Proteobacteria;Gammaproteobacteria;Enterobacteriales;Enterobacteriaceae;Pectobacterium; 31 2 1 9

root;Bacteria;Proteobacteria;Gammaproteobacteria;Enterobacteriales;Enterobacteriaceae;Citrobacter; 52 3 3 1

root;Bacteria;Proteobacteria;Gammaproteobacteria;Enterobacteriales;Enterobacteriaceae;Citrobacter;Unclassified; 52 3 3 1

root;Bacteria;Proteobacteria;Gammaproteobacteria;Enterobacteriales;Enterobacteriaceae;Sodalis; 6 1 0 0

root;Bacteria;Proteobacteria;Gammaproteobacteria;Enterobacteriales;Enterobacteriaceae;Sodalis;Unclassified; 6 1 0 0

root;Bacteria;Proteobacteria;Gammaproteobacteria;Enterobacteriales;Enterobacteriaceae;Serratia; 10 3 6 0

root;Bacteria;Proteobacteria;Gammaproteobacteria;Enterobacteriales;Enterobacteriaceae;Serratia;Unclassified; 10 3 6 0

root;Bacteria;Proteobacteria;Gammaproteobacteria;Enterobacteriales;Enterobacteriaceae;Escherichia; 26 3 1 2

root;Bacteria;Proteobacteria;Gammaproteobacteria;Enterobacteriales;Enterobacteriaceae;Escherichia;Unclassified; 26 3 1 2

root;Bacteria;Proteobacteria;Gammaproteobacteria;Enterobacteriales;Enterobacteriaceae;Buchnera; 5 0 0 0

root;Bacteria;Proteobacteria;Gammaproteobacteria;Enterobacteriales;Enterobacteriaceae;Rahnella; 2 0 0 0

root;Bacteria;Proteobacteria;Gammaproteobacteria;Enterobacteriales;Enterobacteriaceae;Rahnella;Unclassified; 2 0 0 0

root;Bacteria;Proteobacteria;Gammaproteobacteria;Enterobacteriales;Enterobacteriaceae;Buttiauxella; 14 0 0 0

root;Bacteria;Proteobacteria;Gammaproteobacteria;Enterobacteriales;Enterobacteriaceae;Photorhabdus; 1 0 0 0

root;Bacteria;Proteobacteria;Gammaproteobacteria;Enterobacteriales;Enterobacteriaceae;Photorhabdus;Unclassified; 1 0 0 0

root;Bacteria;Proteobacteria;Gammaproteobacteria;Enterobacteriales;Enterobacteriaceae;Salmonella; 8 0 0 0

root;Bacteria;Proteobacteria;Gammaproteobacteria;Enterobacteriales;Enterobacteriaceae;Salmonella;Saintpaul; 8 0 0 0

root;Bacteria;Proteobacteria;Gammaproteobacteria;Enterobacteriales;Enterobacteriaceae;Klebsiella; 2 0 0 0

root;Bacteria;Proteobacteria;Gammaproteobacteria;Enterobacteriales;Enterobacteriaceae;Klebsiella;Unclassified; 2 0 0 0

root;Bacteria;Proteobacteria;Gammaproteobacteria;Enterobacteriales;Enterobacteriaceae;Providencia; 1 0 0 0

root;Bacteria;Proteobacteria;Gammaproteobacteria;Enterobacteriales;Enterobacteriaceae;Providencia;Unclassified; 1 0 0 0

root;Bacteria;Proteobacteria;Gammaproteobacteria;Enterobacteriales;Enterobacteriaceae;Pantoea; 1 0 0 0

root;Bacteria;Proteobacteria;Gammaproteobacteria;Enterobacteriales;Enterobacteriaceae;Pantoea;Unclassified; 1 0 0 0

root;Bacteria;Proteobacteria;Gammaproteobacteria;Enterobacteriales;Enterobacteriaceae;Unclassified; 0 4 4 1

root;Bacteria;Proteobacteria;Gammaproteobacteria;Enterobacteriales;Enterobacteriaceae;Arsenophonus; 0 2 3 0

root;Bacteria;Proteobacteria;Gammaproteobacteria;Enterobacteriales;Enterobacteriaceae;Enterobacter; 0 0 1 1

root;Bacteria;Proteobacteria;Gammaproteobacteria;Enterobacteriales;Enterobacteriaceae;Enterobacter;Unclassified; 0 0 1 1

root;Bacteria;Proteobacteria;Gammaproteobacteria;Alteromonadales; 94 27 9 17

root;Bacteria;Proteobacteria;Gammaproteobacteria;Alteromonadales;Alteromonadaceae; 6 2 2 0

root;Bacteria;Proteobacteria;Gammaproteobacteria;Alteromonadales;Alteromonadaceae;Microbulbifer; 3 2 0 0

root;Bacteria;Proteobacteria;Gammaproteobacteria;Alteromonadales;Alteromonadaceae;Microbulbifer;Unclassified; 3 2 0 0

root;Bacteria;Proteobacteria;Gammaproteobacteria;Alteromonadales;Alteromonadaceae;Marinobacter; 2 0 0 0

root;Bacteria;Proteobacteria;Gammaproteobacteria;Alteromonadales;Alteromonadaceae;Marinobacter;Unclassified; 2 0 0 0

root;Bacteria;Proteobacteria;Gammaproteobacteria;Alteromonadales;Alteromonadaceae;Glaciecola; 1 0 0 0

root;Bacteria;Proteobacteria;Gammaproteobacteria;Alteromonadales;Alteromonadaceae;Glaciecola;Unclassified; 1 0 0 0

root;Bacteria;Proteobacteria;Gammaproteobacteria;Alteromonadales;Alteromonadaceae;Alteromonas; 0 2 0 0

root;Bacteria;Proteobacteria;Gammaproteobacteria;Alteromonadales;Alteromonadaceae;Alteromonas;Unclassified; 0 2 0 0

root;Bacteria;Proteobacteria;Gammaproteobacteria;Alteromonadales;Pseudoalteromonadaceae; 19 21 7 9

root;Bacteria;Proteobacteria;Gammaproteobacteria;Alteromonadales;Pseudoalteromonadaceae;Pseudoalteromonas; 19 21 7 9

root;Bacteria;Proteobacteria;Gammaproteobacteria;Alteromonadales;Pseudoalteromonadaceae;Pseudoalteromonas;Unclassified; 19 21 7 9

root;Bacteria;Proteobacteria;Gammaproteobacteria;Alteromonadales;Shewanellaceae; 49 3 2 5

root;Bacteria;Proteobacteria;Gammaproteobacteria;Alteromonadales;Shewanellaceae;Shewanella; 49 3 2 5

root;Bacteria;Proteobacteria;Gammaproteobacteria;Alteromonadales;Shewanellaceae;Shewanella;Unclassified; 49 3 2 5

root;Bacteria;Proteobacteria;Gammaproteobacteria;Alteromonadales;Colwelliaceae; 19 1 1 0

root;Bacteria;Proteobacteria;Gammaproteobacteria;Alteromonadales;Colwelliaceae;Colwellia; 19 1 1 0

root;Bacteria;Proteobacteria;Gammaproteobacteria;Alteromonadales;Colwelliaceae;Colwellia;Unclassified; 19 1 1 0

root;Bacteria;Proteobacteria;Gammaproteobacteria;Alteromonadales;Psychromonadaceae; 1 0 0 0

root;Bacteria;Proteobacteria;Gammaproteobacteria;Alteromonadales;Psychromonadaceae;Psychromonas; 1 0 0 0

root;Bacteria;Proteobacteria;Gammaproteobacteria;Alteromonadales;Psychromonadaceae;Psychromonas;Unclassified; 1 0 0 0

root;Bacteria;Proteobacteria;Gammaproteobacteria;Cardiobacteriales; 3 3 0 0

root;Bacteria;Proteobacteria;Gammaproteobacteria;Cardiobacteriales;Cardiobacteriaceae; 3 3 0 0

root;Bacteria;Proteobacteria;Gammaproteobacteria;Cardiobacteriales;Cardiobacteriaceae;Dichelobacter; 3 3 0 0

root;Bacteria;Proteobacteria;Gammaproteobacteria;Legionellales; 34 26 6 23

root;Bacteria;Proteobacteria;Gammaproteobacteria;Legionellales;Legionellaceae; 27 21 3 15

root;Bacteria;Proteobacteria;Gammaproteobacteria;Legionellales;Legionellaceae;Legionella; 12 1 3 0

root;Bacteria;Proteobacteria;Gammaproteobacteria;Legionellales;Legionellaceae;Legionella;Unclassified; 12 1 3 0

root;Bacteria;Proteobacteria;Gammaproteobacteria;Legionellales;Legionellaceae;Unclassified; 15 20 3 12

root;Bacteria;Proteobacteria;Gammaproteobacteria;Legionellales;Coxiellaceae; 7 5 3 8

root;Bacteria;Proteobacteria;Gammaproteobacteria;Legionellales;Coxiellaceae;Coxiella; 2 3 0 0

root;Bacteria;Proteobacteria;Gammaproteobacteria;Legionellales;Coxiellaceae;Coxiella;Unclassified; 2 3 0 0

root;Bacteria;Proteobacteria;Gammaproteobacteria;Legionellales;Coxiellaceae;Rickettsiella; 5 5 3 5

root;Bacteria;Proteobacteria;Gammaproteobacteria;Legionellales;Coxiellaceae;Rickettsiella;Unclassified; 5 5 3 5

root;Bacteria;Proteobacteria;Gammaproteobacteria;Chromatiales; 32 13 55 12

root;Bacteria;Proteobacteria;Gammaproteobacteria;Chromatiales;Halothiobacillaceae; 5 0 0 0

root;Bacteria;Proteobacteria;Gammaproteobacteria;Chromatiales;Halothiobacillaceae;Halothiobacillus; 5 0 0 0

root;Bacteria;Proteobacteria;Gammaproteobacteria;Chromatiales;Ectothiorhodospiraceae; 18 4 29 11

root;Bacteria;Proteobacteria;Gammaproteobacteria;Chromatiales;Ectothiorhodospiraceae;Natronocella; 4 0 0 0

root;Bacteria;Proteobacteria;Gammaproteobacteria;Chromatiales;Ectothiorhodospiraceae;Thioalkalivibrio; 9 2 0 0

root;Bacteria;Proteobacteria;Gammaproteobacteria;Chromatiales;Ectothiorhodospiraceae;Thioalkalivibrio;Unclassified; 9 2 0 0

root;Bacteria;Proteobacteria;Gammaproteobacteria;Chromatiales;Ectothiorhodospiraceae;Alkalilimnicola; 4 3 29 8

root;Bacteria;Proteobacteria;Gammaproteobacteria;Chromatiales;Ectothiorhodospiraceae;Halorhodospira; 1 0 0 0

root;Bacteria;Proteobacteria;Gammaproteobacteria;Chromatiales;Ectothiorhodospiraceae;Halorhodospira;Unclassified; 1 0 0 0

root;Bacteria;Proteobacteria;Gammaproteobacteria;Chromatiales;Ectothiorhodospiraceae;Aquasalina; 0 1 1 0

root;Bacteria;Proteobacteria;Gammaproteobacteria;Chromatiales;Chromatiaceae; 9 9 26 1

root;Bacteria;Proteobacteria;Gammaproteobacteria;Chromatiales;Chromatiaceae;Allochromatium; 2 8 24 0

root;Bacteria;Proteobacteria;Gammaproteobacteria;Chromatiales;Chromatiaceae;Chromatium; 6 2 1 0

root;Bacteria;Proteobacteria;Gammaproteobacteria;Chromatiales;Chromatiaceae;Thiocapsa; 1 0 0 0

root;Bacteria;Proteobacteria;Gammaproteobacteria;Chromatiales;Chromatiaceae;Thiocapsa;Unclassified; 1 0 0 0

root;Bacteria;Proteobacteria;Gammaproteobacteria;Chromatiales;Chromatiaceae;Thiorhodococcus; 0 1 0 0

root;Bacteria;Proteobacteria;Gammaproteobacteria;Methylococcales; 6 5 38 15

root;Bacteria;Proteobacteria;Gammaproteobacteria;Methylococcales;Methylococcaceae; 6 5 38 15

root;Bacteria;Proteobacteria;Gammaproteobacteria;Methylococcales;Methylococcaceae;Methylocaldum; 1 1 1 0

root;Bacteria;Proteobacteria;Gammaproteobacteria;Methylococcales;Methylococcaceae;Methylocaldum;Unclassified; 1 1 1 0

root;Bacteria;Proteobacteria;Gammaproteobacteria;Methylococcales;Methylococcaceae;Methylobacter; 4 5 37 14

root;Bacteria;Proteobacteria;Gammaproteobacteria;Methylococcales;Methylococcaceae;Methylobacter;Unclassified; 4 5 37 14

root;Bacteria;Proteobacteria;Gammaproteobacteria;Methylococcales;Methylococcaceae;Methylococcus; 1 0 0 0

root;Bacteria;Proteobacteria;Gammaproteobacteria;Methylococcales;Methylococcaceae;Methylococcus;Unclassified; 1 0 0 0

root;Bacteria;Proteobacteria;Gammaproteobacteria;symbionts; 2 3 13 0

root;Bacteria;Proteobacteria;Gammaproteobacteria;symbionts;Unclassified; 2 3 13 0

root;Bacteria;Proteobacteria;Gammaproteobacteria;Pasteurellales; 47 62 2 101

root;Bacteria;Proteobacteria;Gammaproteobacteria;Pasteurellales;Pasteurellaceae; 47 62 2 101

root;Bacteria;Proteobacteria;Gammaproteobacteria;Pasteurellales;Pasteurellaceae;Unclassified; 9 14 11 0

root;Bacteria;Proteobacteria;Gammaproteobacteria;Pasteurellales;Pasteurellaceae;Pasteurella; 30 46 67 0

root;Bacteria;Proteobacteria;Gammaproteobacteria;Pasteurellales;Pasteurellaceae;Gallibacterium; 3 1 6 0

root;Bacteria;Proteobacteria;Gammaproteobacteria;Pasteurellales;Pasteurellaceae;Haemophilus; 5 13 0 0

root;Bacteria;Proteobacteria;Gammaproteobacteria;Pasteurellales;Pasteurellaceae;Haemophilus;Unclassified; 5 13 0 0

root;Bacteria;Proteobacteria;Gammaproteobacteria;Pasteurellales;Pasteurellaceae;Mannheimia; 0 1 2 4

root;Bacteria;Proteobacteria;Gammaproteobacteria;Thiotrichales; 18 1 1 5

root;Bacteria;Proteobacteria;Gammaproteobacteria;Thiotrichales;Piscirickettsiaceae; 10 1 4 0

root;Bacteria;Proteobacteria;Gammaproteobacteria;Thiotrichales;Piscirickettsiaceae;Methylophaga; 10 3 0 0

root;Bacteria;Proteobacteria;Gammaproteobacteria;Thiotrichales;Piscirickettsiaceae;Methylophaga;Unclassified; 10 3 0 0

root;Bacteria;Proteobacteria;Gammaproteobacteria;Thiotrichales;Piscirickettsiaceae;Thiomicrospira; 0 0 1 1

root;Bacteria;Proteobacteria;Gammaproteobacteria;Thiotrichales;Piscirickettsiaceae;Thiomicrospira;Unclassified; 0 0 1 1

root;Bacteria;Proteobacteria;Gammaproteobacteria;Thiotrichales;Thiotrichaceae; 7 1 0 0

root;Bacteria;Proteobacteria;Gammaproteobacteria;Thiotrichales;Thiotrichaceae;Thioploca; 1 1 0 0

root;Bacteria;Proteobacteria;Gammaproteobacteria;Thiotrichales;Thiotrichaceae;Thiothrix; 2 0 0 0

root;Bacteria;Proteobacteria;Gammaproteobacteria;Thiotrichales;Thiotrichaceae;Thiothrix;Unclassified; 2 0 0 0

root;Bacteria;Proteobacteria;Gammaproteobacteria;Thiotrichales;Thiotrichaceae;Beggiatoa; 4 0 0 0

root;Bacteria;Proteobacteria;Gammaproteobacteria;Thiotrichales;Thiotrichaceae;Beggiatoa;Unclassified; 4 0 0 0

root;Bacteria;Proteobacteria;Gammaproteobacteria;Thiotrichales;Francisellaceae; 1 1 0 0

root;Bacteria;Proteobacteria;Gammaproteobacteria;Thiotrichales;Francisellaceae;Francisella; 1 1 0 0

root;Bacteria;Proteobacteria;Gammaproteobacteria;Thiotrichales;Francisellaceae;Francisella;Unclassified; 1 1 0 0

root;Bacteria;Proteobacteria;Gammaproteobacteria;Aeromonadales; 44 25 1 11

root;Bacteria;Proteobacteria;Gammaproteobacteria;Aeromonadales;Succinivibrionaceae; 5 1 0 0

root;Bacteria;Proteobacteria;Gammaproteobacteria;Aeromonadales;Succinivibrionaceae;Succinimonas; 5 1 0 0

root;Bacteria;Proteobacteria;Gammaproteobacteria;Aeromonadales;Aeromonadaceae; 39 24 1 11

root;Bacteria;Proteobacteria;Gammaproteobacteria;Aeromonadales;Aeromonadaceae;Aeromonas; 34 19 1 5

root;Bacteria;Proteobacteria;Gammaproteobacteria;Aeromonadales;Aeromonadaceae;Aeromonas;Unclassified; 34 19 1 5

root;Bacteria;Proteobacteria;Gammaproteobacteria;Aeromonadales;Aeromonadaceae;Zobellella; 5 5 6 0

root;Bacteria;Proteobacteria;Gammaproteobacteria;Nitrincola; 1 0 0 0

root;Bacteria;Proteobacteria;Gammaproteobacteria;Acidithiobacillales; 19 14 0 0

root;Bacteria;Proteobacteria;Gammaproteobacteria;Acidithiobacillales;Acidithiobacillaceae; 19 14 0 0

root;Bacteria;Proteobacteria;Gammaproteobacteria;Acidithiobacillales;Acidithiobacillaceae;Acidithiobacillus; 19 14 0 0

root;Bacteria;Proteobacteria;Gammaproteobacteria;Acidithiobacillales;Acidithiobacillaceae;Acidithiobacillus;Unclassified; 19 14 0 0

root;Bacteria;Proteobacteria;Gammaproteobacteria;Solimonas; 1 0 0 0

root;Bacteria;Proteobacteria;Gammaproteobacteria;Riesia; 0 4 0 0

root;Bacteria;Proteobacteria;Gammaproteobacteria;Methylonatronum; 0 0 0 6

root;Bacteria;Proteobacteria;Betaproteobacteria; 373 133 305 486

root;Bacteria;Proteobacteria;Betaproteobacteria;Burkholderiales; 126 76 212 82

root;Bacteria;Proteobacteria;Betaproteobacteria;Burkholderiales;Burkholderiaceae; 42 51 205 67

root;Bacteria;Proteobacteria;Betaproteobacteria;Burkholderiales;Burkholderiaceae;Burkholderia; 38 51 205 57

root;Bacteria;Proteobacteria;Betaproteobacteria;Burkholderiales;Burkholderiaceae;Burkholderia;Unclassified; 37 51 205 57

root;Bacteria;Proteobacteria;Betaproteobacteria;Burkholderiales;Burkholderiaceae;Burkholderia;group; 1 0 0 0

root;Bacteria;Proteobacteria;Betaproteobacteria;Burkholderiales;Burkholderiaceae;Ralstonia; 4 10 0 0

root;Bacteria;Proteobacteria;Betaproteobacteria;Burkholderiales;Burkholderiaceae;Ralstonia;Unclassified; 4 10 0 0

root;Bacteria;Proteobacteria;Betaproteobacteria;Burkholderiales;Comamonadaceae; 59 13 3 0

root;Bacteria;Proteobacteria;Betaproteobacteria;Burkholderiales;Comamonadaceae;Hydrogenophaga; 6 1 1 0

root;Bacteria;Proteobacteria;Betaproteobacteria;Burkholderiales;Comamonadaceae;Hydrogenophaga;Unclassified; 6 1 1 0

root;Bacteria;Proteobacteria;Betaproteobacteria;Burkholderiales;Comamonadaceae;Variovorax; 27 1 1 0

root;Bacteria;Proteobacteria;Betaproteobacteria;Burkholderiales;Comamonadaceae;Variovorax;Unclassified; 27 1 1 0

root;Bacteria;Proteobacteria;Betaproteobacteria;Burkholderiales;Comamonadaceae;Rhodoferax; 2 0 0 0

root;Bacteria;Proteobacteria;Betaproteobacteria;Burkholderiales;Comamonadaceae;Rhodoferax;Unclassified; 2 0 0 0

root;Bacteria;Proteobacteria;Betaproteobacteria;Burkholderiales;Comamonadaceae;Acidovorax; 7 0 0 0

root;Bacteria;Proteobacteria;Betaproteobacteria;Burkholderiales;Comamonadaceae;Acidovorax;Unclassified; 7 0 0 0

root;Bacteria;Proteobacteria;Betaproteobacteria;Burkholderiales;Comamonadaceae;Diaphorobacter; 8 0 0 0

root;Bacteria;Proteobacteria;Betaproteobacteria;Burkholderiales;Comamonadaceae;Diaphorobacter;Unclassified; 8 0 0 0

root;Bacteria;Proteobacteria;Betaproteobacteria;Burkholderiales;Comamonadaceae;Comamonas; 6 11 1 0

root;Bacteria;Proteobacteria;Betaproteobacteria;Burkholderiales;Comamonadaceae;Comamonas;Unclassified; 6 11 1 0

root;Bacteria;Proteobacteria;Betaproteobacteria;Burkholderiales;Comamonadaceae;Polaromonas; 1 0 0 0

root;Bacteria;Proteobacteria;Betaproteobacteria;Burkholderiales;Comamonadaceae;Polaromonas;Unclassified; 1 0 0 0

root;Bacteria;Proteobacteria;Betaproteobacteria;Burkholderiales;Comamonadaceae;Delftia; 2 0 0 0

root;Bacteria;Proteobacteria;Betaproteobacteria;Burkholderiales;Comamonadaceae;Delftia;Unclassified; 2 0 0 0

root;Bacteria;Proteobacteria;Betaproteobacteria;Burkholderiales;Oxalobacteraceae; 3 3 4 0

root;Bacteria;Proteobacteria;Betaproteobacteria;Burkholderiales;Oxalobacteraceae;Herbaspirillum; 1 3 0 0

root;Bacteria;Proteobacteria;Betaproteobacteria;Burkholderiales;Oxalobacteraceae;Herbaspirillum;Unclassified; 1 3 0 0

root;Bacteria;Proteobacteria;Betaproteobacteria;Burkholderiales;Oxalobacteraceae;Janthinobacterium; 1 3 1 0

root;Bacteria;Proteobacteria;Betaproteobacteria;Burkholderiales;Oxalobacteraceae;Janthinobacterium;Unclassified; 1 3 1 0

root;Bacteria;Proteobacteria;Betaproteobacteria;Burkholderiales;Oxalobacteraceae;Collimonas; 1 0 0 0

root;Bacteria;Proteobacteria;Betaproteobacteria;Burkholderiales;Oxalobacteraceae;Collimonas;Unclassified; 1 0 0 0

root;Bacteria;Proteobacteria;Betaproteobacteria;Burkholderiales;Rubrivivax; 9 3 1 2

root;Bacteria;Proteobacteria;Betaproteobacteria;Burkholderiales;Rubrivivax;Unclassified; 9 3 1 2

root;Bacteria;Proteobacteria;Betaproteobacteria;Burkholderiales;Alcaligenaceae; 8 4 5 6

root;Bacteria;Proteobacteria;Betaproteobacteria;Burkholderiales;Alcaligenaceae;Bordetella; 7 3 5 5

root;Bacteria;Proteobacteria;Betaproteobacteria;Burkholderiales;Alcaligenaceae;Bordetella;Unclassified; 7 3 5 5

root;Bacteria;Proteobacteria;Betaproteobacteria;Burkholderiales;Alcaligenaceae;Achromobacter; 1 1 0 0

root;Bacteria;Proteobacteria;Betaproteobacteria;Burkholderiales;Alcaligenaceae;Achromobacter;Unclassified; 1 1 0 0

root;Bacteria;Proteobacteria;Betaproteobacteria;Burkholderiales;Alcaligenaceae;Taylorella; 0 0 0 1

root;Bacteria;Proteobacteria;Betaproteobacteria;Burkholderiales;Leptothrix; 3 2 1 0

root;Bacteria;Proteobacteria;Betaproteobacteria;Burkholderiales;Leptothrix;Unclassified; 3 2 1 0

root;Bacteria;Proteobacteria;Betaproteobacteria;Burkholderiales;Thiomonas; 1 0 0 0

root;Bacteria;Proteobacteria;Betaproteobacteria;Burkholderiales;Thiomonas;Unclassified; 1 0 0 0

root;Bacteria;Proteobacteria;Betaproteobacteria;Burkholderiales;Mitsuaria; 1 0 0 0

root;Bacteria;Proteobacteria;Betaproteobacteria;Rhodocyclales; 29 9 26 37

root;Bacteria;Proteobacteria;Betaproteobacteria;Rhodocyclales;Rhodocyclaceae; 29 9 26 37

root;Bacteria;Proteobacteria;Betaproteobacteria;Rhodocyclales;Rhodocyclaceae;Unclassified; 13 5 24 0

root;Bacteria;Proteobacteria;Betaproteobacteria;Rhodocyclales;Rhodocyclaceae;Sterolibacterium; 13 4 26 13

root;Bacteria;Proteobacteria;Betaproteobacteria;Rhodocyclales;Rhodocyclaceae;Sterolibacterium;Unclassified; 13 4 26 13

root;Bacteria;Proteobacteria;Betaproteobacteria;Rhodocyclales;Rhodocyclaceae;Garrityella; 1 0 0 0

root;Bacteria;Proteobacteria;Betaproteobacteria;Rhodocyclales;Rhodocyclaceae;Thauera; 2 0 0 0

root;Bacteria;Proteobacteria;Betaproteobacteria;Rhodocyclales;Rhodocyclaceae;Thauera;Unclassified; 2 0 0 0

root;Bacteria;Proteobacteria;Betaproteobacteria;Unclassified; 21 7 12 16

root;Bacteria;Proteobacteria;Betaproteobacteria;Nitrosomonadales; 6 6 27 14

root;Bacteria;Proteobacteria;Betaproteobacteria;Nitrosomonadales;Nitrosomonadaceae; 6 6 27 14

root;Bacteria;Proteobacteria;Betaproteobacteria;Nitrosomonadales;Nitrosomonadaceae;Nitrosospira; 3 5 27 8

root;Bacteria;Proteobacteria;Betaproteobacteria;Nitrosomonadales;Nitrosomonadaceae;Nitrosospira;Unclassified; 3 5 27 8

root;Bacteria;Proteobacteria;Betaproteobacteria;Nitrosomonadales;Nitrosomonadaceae;Nitrosomonas; 3 1 6 0

root;Bacteria;Proteobacteria;Betaproteobacteria;Nitrosomonadales;Nitrosomonadaceae;Nitrosomonas;Unclassified; 3 1 6 0

root;Bacteria;Proteobacteria;Betaproteobacteria;Neisseriales; 188 34 28 334

root;Bacteria;Proteobacteria;Betaproteobacteria;Neisseriales;Neisseriaceae; 188 34 28 334

root;Bacteria;Proteobacteria;Betaproteobacteria;Neisseriales;Neisseriaceae;Neisseria; 163 26 20 317

root;Bacteria;Proteobacteria;Betaproteobacteria;Neisseriales;Neisseriaceae;Neisseria;Unclassified; 163 26 20 317

root;Bacteria;Proteobacteria;Betaproteobacteria;Neisseriales;Neisseriaceae;Iodobacter; 13 8 7 13

root;Bacteria;Proteobacteria;Betaproteobacteria;Neisseriales;Neisseriaceae;Paludimonas; 4 1 4 0

root;Bacteria;Proteobacteria;Betaproteobacteria;Neisseriales;Neisseriaceae;Chromobacterium; 7 0 0 0

root;Bacteria;Proteobacteria;Betaproteobacteria;Neisseriales;Neisseriaceae;Laribacter; 1 0 0 0

root;Bacteria;Proteobacteria;Betaproteobacteria;Methylophilales; 3 1 3 0

root;Bacteria;Proteobacteria;Betaproteobacteria;Methylophilales;Methylophilaceae; 3 1 3 0

root;Bacteria;Proteobacteria;Betaproteobacteria;Methylophilales;Methylophilaceae;Methylophilus; 3 0 0 0

root;Bacteria;Proteobacteria;Betaproteobacteria;Methylophilales;Methylophilaceae;Methylophilus;Unclassified; 3 0 0 0

root;Bacteria;Proteobacteria;Betaproteobacteria;Methylophilales;Methylophilaceae;Methylobacillus; 0 1 3 0

root;Bacteria;Proteobacteria;Betaproteobacteria;Methylophilales;Methylophilaceae;Methylobacillus;Unclassified; 0 1 3 0

root;Bacteria;Proteobacteria;Alphaproteobacteria; 187 122 190 72

root;Bacteria;Proteobacteria;Alphaproteobacteria;Sphingomonadales; 25 17 60 12

root;Bacteria;Proteobacteria;Alphaproteobacteria;Sphingomonadales;Sphingomonadaceae; 21 17 60 11

root;Bacteria;Proteobacteria;Alphaproteobacteria;Sphingomonadales;Sphingomonadaceae;Sphingomonas; 17 17 53 11

root;Bacteria;Proteobacteria;Alphaproteobacteria;Sphingomonadales;Sphingomonadaceae;Sphingomonas;Unclassified; 17 17 53 11

root;Bacteria;Proteobacteria;Alphaproteobacteria;Sphingomonadales;Sphingomonadaceae;Kaistobacter; 1 7 0 0

root;Bacteria;Proteobacteria;Alphaproteobacteria;Sphingomonadales;Sphingomonadaceae;Kaistobacter;Unclassified; 1 7 0 0

root;Bacteria;Proteobacteria;Alphaproteobacteria;Sphingomonadales;Sphingomonadaceae;Sphingobium; 1 0 0 0

root;Bacteria;Proteobacteria;Alphaproteobacteria;Sphingomonadales;Sphingomonadaceae;Sphingobium;Unclassified; 1 0 0 0

root;Bacteria;Proteobacteria;Alphaproteobacteria;Sphingomonadales;Sphingomonadaceae;Zymomonas; 2 0 0 0

root;Bacteria;Proteobacteria;Alphaproteobacteria;Sphingomonadales;Erythrobacteraceae; 4 1 0 0

root;Bacteria;Proteobacteria;Alphaproteobacteria;Sphingomonadales;Erythrobacteraceae;Erythrobacter; 4 1 0 0

root;Bacteria;Proteobacteria;Alphaproteobacteria;Sphingomonadales;Erythrobacteraceae;Erythrobacter;Unclassified; 4 1 0 0

root;Bacteria;Proteobacteria;Alphaproteobacteria;Rhizobiales; 45 39 70 30

root;Bacteria;Proteobacteria;Alphaproteobacteria;Rhizobiales;Bradyrhizobiaceae; 4 3 2 0

root;Bacteria;Proteobacteria;Alphaproteobacteria;Rhizobiales;Bradyrhizobiaceae;Nitrobacter; 2 0 0 0

root;Bacteria;Proteobacteria;Alphaproteobacteria;Rhizobiales;Bradyrhizobiaceae;Nitrobacter;Unclassified; 2 0 0 0

root;Bacteria;Proteobacteria;Alphaproteobacteria;Rhizobiales;Bradyrhizobiaceae;Afipia; 2 3 1 0

root;Bacteria;Proteobacteria;Alphaproteobacteria;Rhizobiales;Bradyrhizobiaceae;Afipia;Unclassified; 2 3 1 0

root;Bacteria;Proteobacteria;Alphaproteobacteria;Rhizobiales;Bradyrhizobiaceae;Bradyrhizobium; 0 0 1 0

root;Bacteria;Proteobacteria;Alphaproteobacteria;Rhizobiales;Bradyrhizobiaceae;Bradyrhizobium;Unclassified; 0 0 1 0

root;Bacteria;Proteobacteria;Alphaproteobacteria;Rhizobiales;Hyphomicrobiaceae; 18 4 2 0

root;Bacteria;Proteobacteria;Alphaproteobacteria;Rhizobiales;Hyphomicrobiaceae;Hyphomicrobium; 16 2 0 0

root;Bacteria;Proteobacteria;Alphaproteobacteria;Rhizobiales;Hyphomicrobiaceae;Hyphomicrobium;Unclassified; 16 2 0 0

root;Bacteria;Proteobacteria;Alphaproteobacteria;Rhizobiales;Hyphomicrobiaceae;Devosia; 2 4 0 0

root;Bacteria;Proteobacteria;Alphaproteobacteria;Rhizobiales;Hyphomicrobiaceae;Devosia;Unclassified; 2 4 0 0

root;Bacteria;Proteobacteria;Alphaproteobacteria;Rhizobiales;Phyllobacteriaceae; 3 16 48 23

root;Bacteria;Proteobacteria;Alphaproteobacteria;Rhizobiales;Phyllobacteriaceae;Mesorhizobium; 3 16 48 23

root;Bacteria;Proteobacteria;Alphaproteobacteria;Rhizobiales;Phyllobacteriaceae;Mesorhizobium;Unclassified; 3 16 48 23

root;Bacteria;Proteobacteria;Alphaproteobacteria;Rhizobiales;Xanthobacteraceae; 2 12 17 1

root;Bacteria;Proteobacteria;Alphaproteobacteria;Rhizobiales;Xanthobacteraceae;Azorhizobium; 2 12 17 1

root;Bacteria;Proteobacteria;Alphaproteobacteria;Rhizobiales;Unclassified; 3 3 2 0

root;Bacteria;Proteobacteria;Alphaproteobacteria;Rhizobiales;Methylobacteriaceae; 6 0 0 0

root;Bacteria;Proteobacteria;Alphaproteobacteria;Rhizobiales;Methylobacteriaceae;Methylobacterium; 2 0 0 0

root;Bacteria;Proteobacteria;Alphaproteobacteria;Rhizobiales;Methylobacteriaceae;Methylobacterium;Unclassified; 2 0 0 0

root;Bacteria;Proteobacteria;Alphaproteobacteria;Rhizobiales;Methylobacteriaceae;Microvirga; 1 0 0 0

root;Bacteria;Proteobacteria;Alphaproteobacteria;Rhizobiales;Methylobacteriaceae;Roseomonas; 3 0 0 0

root;Bacteria;Proteobacteria;Alphaproteobacteria;Rhizobiales;Rhizobiaceae; 5 2 2 0

root;Bacteria;Proteobacteria;Alphaproteobacteria;Rhizobiales;Rhizobiaceae;Rhizobium; 3 1 0 0

root;Bacteria;Proteobacteria;Alphaproteobacteria;Rhizobiales;Rhizobiaceae;Rhizobium;Unclassified; 3 1 0 0

root;Bacteria;Proteobacteria;Alphaproteobacteria;Rhizobiales;Rhizobiaceae;Liberibacter; 1 0 0 0

root;Bacteria;Proteobacteria;Alphaproteobacteria;Rhizobiales;Rhizobiaceae;group; 1 1 1 0

root;Bacteria;Proteobacteria;Alphaproteobacteria;Rhizobiales;Rhizobiaceae;group;Rhizobium; 1 1 1 0

root;Bacteria;Proteobacteria;Alphaproteobacteria;Rhizobiales;Rhizobiaceae;group;Rhizobium;Unclassified; 1 1 1 0

root;Bacteria;Proteobacteria;Alphaproteobacteria;Rhizobiales;Rhizobiaceae;Agrobacterium; 0 0 0 1

root;Bacteria;Proteobacteria;Alphaproteobacteria;Rhizobiales;Rhizobiaceae;Agrobacterium;Unclassified; 0 0 0 1

root;Bacteria;Proteobacteria;Alphaproteobacteria;Rhizobiales;Kaistina; 3 1 0 0

root;Bacteria;Proteobacteria;Alphaproteobacteria;Rhizobiales;Methylocystaceae; 1 0 0 0

root;Bacteria;Proteobacteria;Alphaproteobacteria;Rhizobiales;Methylocystaceae;Methylocystis; 1 0 0 0

root;Bacteria;Proteobacteria;Alphaproteobacteria;Rhizobiales;Methylocystaceae;Methylocystis;Unclassified; 1 0 0 0

root;Bacteria;Proteobacteria;Alphaproteobacteria;Rhizobiales;Beijerinckiaceae; 0 1 0 0

root;Bacteria;Proteobacteria;Alphaproteobacteria;Rhizobiales;Beijerinckiaceae;Chelatococcus; 0 1 0 0

root;Bacteria;Proteobacteria;Alphaproteobacteria;Rhizobiales;Beijerinckiaceae;Chelatococcus;Unclassified; 0 1 0 0

root;Bacteria;Proteobacteria;Alphaproteobacteria;Unclassified; 76 36 25 26

root;Bacteria;Proteobacteria;Alphaproteobacteria;Rhodospirillales; 34 16 9 1

root;Bacteria;Proteobacteria;Alphaproteobacteria;Rhodospirillales;Rhodospirillaceae; 28 15 9 1

root;Bacteria;Proteobacteria;Alphaproteobacteria;Rhodospirillales;Rhodospirillaceae;Unclassified; 4 4 7 1

root;Bacteria;Proteobacteria;Alphaproteobacteria;Rhodospirillales;Rhodospirillaceae;Rhodocista; 11 11 1 0

root;Bacteria;Proteobacteria;Alphaproteobacteria;Rhodospirillales;Rhodospirillaceae;Rhodocista;Unclassified; 11 11 1 0

root;Bacteria;Proteobacteria;Alphaproteobacteria;Rhodospirillales;Rhodospirillaceae;Magnetospirillum; 9 0 0 0

root;Bacteria;Proteobacteria;Alphaproteobacteria;Rhodospirillales;Rhodospirillaceae;Magnetospirillum;Unclassified; 9 0 0 0

root;Bacteria;Proteobacteria;Alphaproteobacteria;Rhodospirillales;Rhodospirillaceae;Azospirillum; 4 1 0 0

root;Bacteria;Proteobacteria;Alphaproteobacteria;Rhodospirillales;Rhodospirillaceae;Azospirillum;Unclassified; 4 1 0 0

root;Bacteria;Proteobacteria;Alphaproteobacteria;Rhodospirillales;Acetobacteraceae; 6 1 0 0

root;Bacteria;Proteobacteria;Alphaproteobacteria;Rhodospirillales;Acetobacteraceae;Stella; 4 0 0 0

root;Bacteria;Proteobacteria;Alphaproteobacteria;Rhodospirillales;Acetobacteraceae;Stella;Unclassified; 4 0 0 0

root;Bacteria;Proteobacteria;Alphaproteobacteria;Rhodospirillales;Acetobacteraceae;Acetobacter; 2 1 0 0

root;Bacteria;Proteobacteria;Alphaproteobacteria;Rhodospirillales;Acetobacteraceae;Acetobacter;Unclassified; 2 1 0 0

root;Bacteria;Proteobacteria;Alphaproteobacteria;Kopriimonadales; 1 0 0 0

root;Bacteria;Proteobacteria;Alphaproteobacteria;Kopriimonadales;Kopriimonadaceae; 1 0 0 0

root;Bacteria;Proteobacteria;Alphaproteobacteria;Kopriimonadales;Kopriimonadaceae;Kopriimonas; 1 0 0 0

root;Bacteria;Proteobacteria;Alphaproteobacteria;Rickettsiales; 3 1 0 0

root;Bacteria;Proteobacteria;Alphaproteobacteria;Rickettsiales;Odyssella; 1 0 0 0

root;Bacteria;Proteobacteria;Alphaproteobacteria;Rickettsiales;Odyssella;Unclassified; 1 0 0 0

root;Bacteria;Proteobacteria;Alphaproteobacteria;Rickettsiales;Rickettsiaceae; 1 0 0 0

root;Bacteria;Proteobacteria;Alphaproteobacteria;Rickettsiales;Rickettsiaceae;Rickettsieae; 1 0 0 0

root;Bacteria;Proteobacteria;Alphaproteobacteria;Rickettsiales;Rickettsiaceae;Rickettsieae;Rickettsia; 1 0 0 0

root;Bacteria;Proteobacteria;Alphaproteobacteria;Rickettsiales;Rickettsiaceae;Rickettsieae;Rickettsia;group; 1 0 0 0

root;Bacteria;Proteobacteria;Alphaproteobacteria;Rickettsiales;Rickettsiaceae;Rickettsieae;Rickettsia;group;Unclassified; 1 0 0 0

root;Bacteria;Proteobacteria;Alphaproteobacteria;Rickettsiales;Anaplasmataceae; 1 0 0 0

root;Bacteria;Proteobacteria;Alphaproteobacteria;Rickettsiales;Anaplasmataceae;Anaplasma; 1 0 0 0

root;Bacteria;Proteobacteria;Alphaproteobacteria;Rickettsiales;Anaplasmataceae;Anaplasma;Unclassified; 1 0 0 0

root;Bacteria;Proteobacteria;Alphaproteobacteria;Rickettsiales;Holosporaceae; 0 0 1 0

root;Bacteria;Proteobacteria;Alphaproteobacteria;Rickettsiales;Holosporaceae;Unclassified; 0 0 1 0

root;Bacteria;Proteobacteria;Alphaproteobacteria;Rhodobacterales; 3 14 25 3

root;Bacteria;Proteobacteria;Alphaproteobacteria;Rhodobacterales;Rhodobacteraceae; 3 12 25 3

root;Bacteria;Proteobacteria;Alphaproteobacteria;Rhodobacterales;Rhodobacteraceae;Roseinatronobacter; 1 9 2 0

root;Bacteria;Proteobacteria;Alphaproteobacteria;Rhodobacterales;Rhodobacteraceae;Rhodobacter; 1 3 23 2

root;Bacteria;Proteobacteria;Alphaproteobacteria;Rhodobacterales;Rhodobacteraceae;Rhodobacter;Unclassified; 1 3 23 2

root;Bacteria;Proteobacteria;Alphaproteobacteria;Rhodobacterales;Rhodobacteraceae;Paracoccus; 1 1 0 0

root;Bacteria;Proteobacteria;Alphaproteobacteria;Rhodobacterales;Rhodobacteraceae;Paracoccus;Unclassified; 1 1 0 0

root;Bacteria;Proteobacteria;Alphaproteobacteria;Rhodobacterales;Hyphomonadaceae; 0 2 0 0

root;Bacteria;Proteobacteria;Alphaproteobacteria;Rhodobacterales;Hyphomonadaceae;Maricaulis; 0 2 0 0

root;Bacteria;Proteobacteria;Epsilonproteobacteria; 241 57 3 25

root;Bacteria;Proteobacteria;Epsilonproteobacteria;Campylobacterales; 233 49 1 22

root;Bacteria;Proteobacteria;Epsilonproteobacteria;Campylobacterales;Campylobacteraceae; 178 33 19 0

root;Bacteria;Proteobacteria;Epsilonproteobacteria;Campylobacterales;Campylobacteraceae;Sulfurospirillum; 10 5 1 0

root;Bacteria;Proteobacteria;Epsilonproteobacteria;Campylobacterales;Campylobacteraceae;Sulfurospirillum;Unclassified; 10 5 1 0

root;Bacteria;Proteobacteria;Epsilonproteobacteria;Campylobacterales;Campylobacteraceae;Campylobacter; 148 27 18 0

root;Bacteria;Proteobacteria;Epsilonproteobacteria;Campylobacterales;Campylobacteraceae;Campylobacter;Unclassified; 148 27 18 0

root;Bacteria;Proteobacteria;Epsilonproteobacteria;Campylobacterales;Campylobacteraceae;Arcobacter; 20 1 0 0

root;Bacteria;Proteobacteria;Epsilonproteobacteria;Campylobacterales;Campylobacteraceae;Arcobacter;Unclassified; 20 1 0 0

root;Bacteria;Proteobacteria;Epsilonproteobacteria;Campylobacterales;Helicobacteraceae; 49 16 1 2

root;Bacteria;Proteobacteria;Epsilonproteobacteria;Campylobacterales;Helicobacteraceae;Helicobacter; 35 16 1 2

root;Bacteria;Proteobacteria;Epsilonproteobacteria;Campylobacterales;Helicobacteraceae;Helicobacter;Unclassified; 35 16 1 2

root;Bacteria;Proteobacteria;Epsilonproteobacteria;Campylobacterales;Helicobacteraceae;Sulfurimonas; 14 0 0 0

root;Bacteria;Proteobacteria;Epsilonproteobacteria;Campylobacterales;Helicobacteraceae;Sulfurimonas;Unclassified; 14 0 0 0

root;Bacteria;Proteobacteria;Epsilonproteobacteria;Campylobacterales;Unclassified; 6 1 0 0

root;Bacteria;Proteobacteria;Epsilonproteobacteria;Nitratiruptor; 2 2 0 0

root;Bacteria;Proteobacteria;Epsilonproteobacteria;Sulfurovum; 6 6 2 3

root;Bacteria;Proteobacteria;Epsilonproteobacteria;Sulfurovum;Unclassified; 6 6 2 3

root;Bacteria;Proteobacteria;Unclassified; 20 3 7 0

root;Bacteria;Proteobacteria;Magnetococcus; 4 2 3 2

root;Bacteria;Proteobacteria;Magnetococcus;Unclassified; 4 2 3 2

root;Bacteria;Firmicutes; 6206 2819 770 295

root;Bacteria;Firmicutes;Lactobacillales; 1482 380 123 70

root;Bacteria;Firmicutes;Lactobacillales;Streptococcaceae; 1253 325 112 53

root;Bacteria;Firmicutes;Lactobacillales;Streptococcaceae;Streptococcus; 1243 325 112 52

root;Bacteria;Firmicutes;Lactobacillales;Streptococcaceae;Streptococcus;Unclassified; 1243 325 112 52

root;Bacteria;Firmicutes;Lactobacillales;Streptococcaceae;Lactococcus; 10 1 0 0

root;Bacteria;Firmicutes;Lactobacillales;Streptococcaceae;Lactococcus;Unclassified; 10 1 0 0

root;Bacteria;Firmicutes;Lactobacillales;Weissella; 16 8 2 1

root;Bacteria;Firmicutes;Lactobacillales;Weissella;Unclassified; 16 8 2 1

root;Bacteria;Firmicutes;Lactobacillales;Carnobacteriaceae; 40 4 2 0

root;Bacteria;Firmicutes;Lactobacillales;Carnobacteriaceae;Carnobacterium; 37 3 2 0

root;Bacteria;Firmicutes;Lactobacillales;Carnobacteriaceae;Carnobacterium;Unclassified; 37 3 2 0

root;Bacteria;Firmicutes;Lactobacillales;Carnobacteriaceae;Alkalibacterium; 1 0 0 0

root;Bacteria;Firmicutes;Lactobacillales;Carnobacteriaceae;Alkalibacterium;Unclassified; 1 0 0 0

root;Bacteria;Firmicutes;Lactobacillales;Carnobacteriaceae;Marinilactibacillus; 2 1 0 0

root;Bacteria;Firmicutes;Lactobacillales;Lactobacillaceae; 51 17 9 9

root;Bacteria;Firmicutes;Lactobacillales;Lactobacillaceae;Lactobacillus; 48 17 9 9

root;Bacteria;Firmicutes;Lactobacillales;Lactobacillaceae;Lactobacillus;Unclassified; 48 17 9 9

root;Bacteria;Firmicutes;Lactobacillales;Lactobacillaceae;Pediococcus; 2 0 0 0

root;Bacteria;Firmicutes;Lactobacillales;Lactobacillaceae;Unclassified; 1 0 0 0

root;Bacteria;Firmicutes;Lactobacillales;Enterococcaceae; 90 18 4 0

root;Bacteria;Firmicutes;Lactobacillales;Enterococcaceae;Enterococcus; 90 18 4 0

root;Bacteria;Firmicutes;Lactobacillales;Enterococcaceae;Enterococcus;Unclassified; 90 18 4 0

root;Bacteria;Firmicutes;Lactobacillales;Aerococcaceae; 7 2 0 0

root;Bacteria;Firmicutes;Lactobacillales;Aerococcaceae;Aerococcus; 6 1 0 0

root;Bacteria;Firmicutes;Lactobacillales;Aerococcaceae;Aerococcus;Unclassified; 6 1 0 0

root;Bacteria;Firmicutes;Lactobacillales;Aerococcaceae;Globicatella; 1 0 0 0

root;Bacteria;Firmicutes;Lactobacillales;Aerococcaceae;Facklamia; 0 1 0 0

root;Bacteria;Firmicutes;Lactobacillales;Aerococcaceae;Facklamia;Unclassified; 0 1 0 0

root;Bacteria;Firmicutes;Lactobacillales;Oscillospiraceae; 18 6 0 0

root;Bacteria;Firmicutes;Lactobacillales;Oscillospiraceae;Oscillospira; 18 6 0 0

root;Bacteria;Firmicutes;Lactobacillales;Leuconostoc; 1 0 0 0

root;Bacteria;Firmicutes;Lactobacillales;Leuconostoc;Unclassified; 1 0 0 0

root;Bacteria;Firmicutes;Bacillales; 436 295 136 22

root;Bacteria;Firmicutes;Bacillales;Unclassified; 28 3 1 1

root;Bacteria;Firmicutes;Bacillales;Bacillaceae; 245 181 129 10

root;Bacteria;Firmicutes;Bacillales;Bacillaceae;Bacillus; 219 135 121 8

root;Bacteria;Firmicutes;Bacillales;Bacillaceae;Bacillus;Unclassified; 204 125 121 8

root;Bacteria;Firmicutes;Bacillales;Bacillaceae;Bacillus;group; 15 10 0 0

root;Bacteria;Firmicutes;Bacillales;Bacillaceae;Geobacillus; 4 5 0 0

root;Bacteria;Firmicutes;Bacillales;Bacillaceae;Geobacillus;Unclassified; 4 5 0 0

root;Bacteria;Firmicutes;Bacillales;Bacillaceae;Exiguobacterium; 17 37 8 2

root;Bacteria;Firmicutes;Bacillales;Bacillaceae;Exiguobacterium;Unclassified; 17 37 8 2

root;Bacteria;Firmicutes;Bacillales;Bacillaceae;Anoxybacillus; 3 2 0 0

root;Bacteria;Firmicutes;Bacillales;Bacillaceae;Anoxybacillus;Unclassified; 3 2 0 0

root;Bacteria;Firmicutes;Bacillales;Bacillaceae;Lysinibacillus; 1 2 0 0

root;Bacteria;Firmicutes;Bacillales;Bacillaceae;Lentibacillus; 1 0 0 0

root;Bacteria;Firmicutes;Bacillales;Bacillaceae;Lentibacillus;Unclassified; 1 0 0 0

root;Bacteria;Firmicutes;Bacillales;Paenibacillaceae; 40 74 6 3

root;Bacteria;Firmicutes;Bacillales;Paenibacillaceae;Paenibacillus; 23 30 4 3

root;Bacteria;Firmicutes;Bacillales;Paenibacillaceae;Paenibacillus;Unclassified; 23 30 4 3

root;Bacteria;Firmicutes;Bacillales;Paenibacillaceae;Brevibacillus; 17 40 2 0

root;Bacteria;Firmicutes;Bacillales;Paenibacillaceae;Brevibacillus;Unclassified; 17 40 2 0

root;Bacteria;Firmicutes;Bacillales;Paenibacillaceae;Unclassified; 0 4 0 0

root;Bacteria;Firmicutes;Bacillales;Gemella; 51 6 7 0

root;Bacteria;Firmicutes;Bacillales;Gemella;Unclassified; 51 6 7 0

root;Bacteria;Firmicutes;Bacillales;Staphylococcus; 28 1 0 0

root;Bacteria;Firmicutes;Bacillales;Staphylococcus;Unclassified; 28 1 0 0

root;Bacteria;Firmicutes;Bacillales;Alicyclobacillaceae; 19 12 0 0

root;Bacteria;Firmicutes;Bacillales;Alicyclobacillaceae;Sulfobacillus; 12 10 0 0

root;Bacteria;Firmicutes;Bacillales;Alicyclobacillaceae;Sulfobacillus;Unclassified; 12 10 0 0

root;Bacteria;Firmicutes;Bacillales;Alicyclobacillaceae;Alicyclobacillus; 7 2 0 0

root;Bacteria;Firmicutes;Bacillales;Alicyclobacillaceae;Alicyclobacillus;Unclassified; 7 2 0 0

root;Bacteria;Firmicutes;Bacillales;Sporolactobacillaceae; 8 7 1 0

root;Bacteria;Firmicutes;Bacillales;Sporolactobacillaceae;Sporolactobacillus; 8 7 1 0

root;Bacteria;Firmicutes;Bacillales;Salinicoccus; 1 0 0 0

root;Bacteria;Firmicutes;Bacillales;Listeriaceae; 4 2 0 0

root;Bacteria;Firmicutes;Bacillales;Listeriaceae;Listeria; 4 2 0 0

root;Bacteria;Firmicutes;Bacillales;Thermoactinomycetaceae; 11 9 0 0

root;Bacteria;Firmicutes;Bacillales;Thermoactinomycetaceae;Planifilum; 11 9 0 0

root;Bacteria;Firmicutes;Bacillales;Macrococcus; 1 0 0 0

root;Bacteria;Firmicutes;Clostridia; 3895 1964 474 189

root;Bacteria;Firmicutes;Clostridia;Clostridiales; 3796 1916 465 185

root;Bacteria;Firmicutes;Clostridia;Clostridiales;Clostridiaceae; 1692 738 332 89

root;Bacteria;Firmicutes;Clostridia;Clostridiales;Clostridiaceae;Clostridium; 1545 705 330 79

root;Bacteria;Firmicutes;Clostridia;Clostridiales;Clostridiaceae;Clostridium;Unclassified; 1545 705 330 79

root;Bacteria;Firmicutes;Clostridia;Clostridiales;Clostridiaceae;Unclassified; 73 8 5 0

root;Bacteria;Firmicutes;Clostridia;Clostridiales;Clostridiaceae;Faecalibacterium; 12 14 0 0

root;Bacteria;Firmicutes;Clostridia;Clostridiales;Clostridiaceae;Faecalibacterium;Unclassified; 12 14 0 0

root;Bacteria;Firmicutes;Clostridia;Clostridiales;Clostridiaceae;Alkaliphilus; 5 0 0 0

root;Bacteria;Firmicutes;Clostridia;Clostridiales;Clostridiaceae;Alkaliphilus;Unclassified; 5 0 0 0

root;Bacteria;Firmicutes;Clostridia;Clostridiales;Clostridiaceae;Tepidimicrobium; 1 0 0 0

root;Bacteria;Firmicutes;Clostridia;Clostridiales;Clostridiaceae;Geosporobacter; 10 2 1 0

root;Bacteria;Firmicutes;Clostridia;Clostridiales;Clostridiaceae;Tindallia; 21 5 4 0

root;Bacteria;Firmicutes;Clostridia;Clostridiales;Clostridiaceae;Acetivibrio; 12 3 0 0

root;Bacteria;Firmicutes;Clostridia;Clostridiales;Clostridiaceae;Caloramator; 2 1 2 0

root;Bacteria;Firmicutes;Clostridia;Clostridiales;Clostridiaceae;Caloramator;Unclassified; 2 1 2 0

root;Bacteria;Firmicutes;Clostridia;Clostridiales;Clostridiaceae;Dorea; 9 0 0 0

root;Bacteria;Firmicutes;Clostridia;Clostridiales;Clostridiaceae;Dorea;Unclassified; 9 0 0 0

root;Bacteria;Firmicutes;Clostridia;Clostridiales;Clostridiaceae;Gracilibacter; 2 0 0 0

root;Bacteria;Firmicutes;Clostridia;Clostridiales;Peptostreptococcaceae; 26 18 1 10

root;Bacteria;Firmicutes;Clostridia;Clostridiales;Peptostreptococcaceae;Peptoniphilus; 20 9 0 0

root;Bacteria;Firmicutes;Clostridia;Clostridiales;Peptostreptococcaceae;Peptoniphilus;Unclassified; 20 9 0 0

root;Bacteria;Firmicutes;Clostridia;Clostridiales;Peptostreptococcaceae;Sporanaerobacter; 1 0 0 0

root;Bacteria;Firmicutes;Clostridia;Clostridiales;Peptostreptococcaceae;Peptostreptococcus; 5 2 7 0

root;Bacteria;Firmicutes;Clostridia;Clostridiales;Peptostreptococcaceae;Peptostreptococcus;Unclassified; 5 2 7 0

root;Bacteria;Firmicutes;Clostridia;Clostridiales;Peptostreptococcaceae;Sedimentibacter; 0 4 1 3

root;Bacteria;Firmicutes;Clostridia;Clostridiales;Peptostreptococcaceae;Sedimentibacter;Unclassified; 0 4 1 3

root;Bacteria;Firmicutes;Clostridia;Clostridiales;Peptostreptococcaceae;Anaerococcus; 0 3 0 0

root;Bacteria;Firmicutes;Clostridia;Clostridiales;Peptostreptococcaceae;Anaerococcus;Unclassified; 0 3 0 0

root;Bacteria;Firmicutes;Clostridia;Clostridiales;Acidaminococcaceae; 1273 775 16 25

root;Bacteria;Firmicutes;Clostridia;Clostridiales;Acidaminococcaceae;Schwartzia; 305 240 12 5

root;Bacteria;Firmicutes;Clostridia;Clostridiales;Acidaminococcaceae;Megasphaera; 36 24 0 0

root;Bacteria;Firmicutes;Clostridia;Clostridiales;Acidaminococcaceae;Megasphaera;Unclassified; 36 24 0 0

root;Bacteria;Firmicutes;Clostridia;Clostridiales;Acidaminococcaceae;Acidaminococcus; 65 105 3 0

root;Bacteria;Firmicutes;Clostridia;Clostridiales;Acidaminococcaceae;Acidaminococcus;Unclassified; 65 105 3 0

root;Bacteria;Firmicutes;Clostridia;Clostridiales;Acidaminococcaceae;Selenomonas; 109 17 1 0

root;Bacteria;Firmicutes;Clostridia;Clostridiales;Acidaminococcaceae;Selenomonas;Unclassified; 109 17 1 0

root;Bacteria;Firmicutes;Clostridia;Clostridiales;Acidaminococcaceae;Anaeroglobus; 43 82 1 0

root;Bacteria;Firmicutes;Clostridia;Clostridiales;Acidaminococcaceae;Dialister; 33 49 1 0

root;Bacteria;Firmicutes;Clostridia;Clostridiales;Acidaminococcaceae;Dialister;Unclassified; 33 49 1 0

root;Bacteria;Firmicutes;Clostridia;Clostridiales;Acidaminococcaceae;Veillonella; 644 228 15 0

root;Bacteria;Firmicutes;Clostridia;Clostridiales;Acidaminococcaceae;Veillonella;Unclassified; 644 228 15 0

root;Bacteria;Firmicutes;Clostridia;Clostridiales;Acidaminococcaceae;Sporomusa; 38 30 1 1

root;Bacteria;Firmicutes;Clostridia;Clostridiales;Acidaminococcaceae;Sporomusa;Unclassified; 38 30 1 1

root;Bacteria;Firmicutes;Clostridia;Clostridiales;Acidaminococcaceae;Thermosinus; 0 0 0 1

root;Bacteria;Firmicutes;Clostridia;Clostridiales;Veillonellaceae; 62 23 0 0

root;Bacteria;Firmicutes;Clostridia;Clostridiales;Veillonellaceae;Acidaminococcus; 27 18 0 0

root;Bacteria;Firmicutes;Clostridia;Clostridiales;Veillonellaceae;Unclassified; 35 5 0 0

root;Bacteria;Firmicutes;Clostridia;Clostridiales;Epulopiscium; 4 4 1 1

root;Bacteria;Firmicutes;Clostridia;Clostridiales;Epulopiscium;Unclassified; 4 4 1 1

root;Bacteria;Firmicutes;Clostridia;Clostridiales;Unclassified; 119 33 9 16

root;Bacteria;Firmicutes;Clostridia;Clostridiales;Peptococcaceae; 175 131 19 8

root;Bacteria;Firmicutes;Clostridia;Clostridiales;Peptococcaceae;Pelotomaculum; 52 75 6 4

root;Bacteria;Firmicutes;Clostridia;Clostridiales;Peptococcaceae;Pelotomaculum;Unclassified; 52 75 6 4

root;Bacteria;Firmicutes;Clostridia;Clostridiales;Peptococcaceae;Desulfotomaculum; 54 18 8 3

root;Bacteria;Firmicutes;Clostridia;Clostridiales;Peptococcaceae;Desulfotomaculum;Unclassified; 54 18 8 3

root;Bacteria;Firmicutes;Clostridia;Clostridiales;Peptococcaceae;Carboxydothermus; 4 2 0 0

root;Bacteria;Firmicutes;Clostridia;Clostridiales;Peptococcaceae;Desulfosporosinus; 25 3 0 0

root;Bacteria;Firmicutes;Clostridia;Clostridiales;Peptococcaceae;Desulfosporosinus;Unclassified; 25 3 0 0

root;Bacteria;Firmicutes;Clostridia;Clostridiales;Peptococcaceae;Unclassified; 21 14 1 0

root;Bacteria;Firmicutes;Clostridia;Clostridiales;Peptococcaceae;Cryptanaerobacter; 3 0 0 0

root;Bacteria;Firmicutes;Clostridia;Clostridiales;Peptococcaceae;Desulfitobacterium; 14 15 4 1

root;Bacteria;Firmicutes;Clostridia;Clostridiales;Peptococcaceae;Desulfitobacterium;Unclassified; 14 15 4 1

root;Bacteria;Firmicutes;Clostridia;Clostridiales;Peptococcaceae;Peptococcus; 1 3 0 0

root;Bacteria;Firmicutes;Clostridia;Clostridiales;Peptococcaceae;Peptococcus;Unclassified; 1 3 0 0

root;Bacteria;Firmicutes;Clostridia;Clostridiales;Peptococcaceae;Dehalobacter; 1 1 0 0

root;Bacteria;Firmicutes;Clostridia;Clostridiales;Peptococcaceae;Dehalobacter;Unclassified; 1 1 0 0

root;Bacteria;Firmicutes;Clostridia;Clostridiales;Eubacteriaceae; 132 76 42 25

root;Bacteria;Firmicutes;Clostridia;Clostridiales;Eubacteriaceae;Eubacterium; 119 72 41 24

root;Bacteria;Firmicutes;Clostridia;Clostridiales;Eubacteriaceae;Eubacterium;Unclassified; 119 72 41 24

root;Bacteria;Firmicutes;Clostridia;Clostridiales;Eubacteriaceae;Mogibacterium; 10 1 1 0

root;Bacteria;Firmicutes;Clostridia;Clostridiales;Eubacteriaceae;Mogibacterium;Unclassified; 10 1 1 0

root;Bacteria;Firmicutes;Clostridia;Clostridiales;Eubacteriaceae;Pseudoramibacter; 1 1 0 0

root;Bacteria;Firmicutes;Clostridia;Clostridiales;Eubacteriaceae;Unclassified; 1 3 0 0

root;Bacteria;Firmicutes;Clostridia;Clostridiales;Eubacteriaceae;Acetobacterium; 1 0 0 0

root;Bacteria;Firmicutes;Clostridia;Clostridiales;Eubacteriaceae;Acetobacterium;Unclassified; 1 0 0 0

root;Bacteria;Firmicutes;Clostridia;Clostridiales;Lachnospiraceae; 264 106 38 10

root;Bacteria;Firmicutes;Clostridia;Clostridiales;Lachnospiraceae;Roseburia; 3 0 0 0

root;Bacteria;Firmicutes;Clostridia;Clostridiales;Lachnospiraceae;Roseburia;Unclassified; 3 0 0 0

root;Bacteria;Firmicutes;Clostridia;Clostridiales;Lachnospiraceae;Dorea; 6 1 0 0

root;Bacteria;Firmicutes;Clostridia;Clostridiales;Lachnospiraceae;Ruminococcus; 121 43 17 6

root;Bacteria;Firmicutes;Clostridia;Clostridiales;Lachnospiraceae;Ruminococcus;Unclassified; 121 43 17 6

root;Bacteria;Firmicutes;Clostridia;Clostridiales;Lachnospiraceae;Butyrivibrio; 81 24 18 3

root;Bacteria;Firmicutes;Clostridia;Clostridiales;Lachnospiraceae;Butyrivibrio;Unclassified; 81 24 18 3

root;Bacteria;Firmicutes;Clostridia;Clostridiales;Lachnospiraceae;Anaerostipes; 16 4 1 0

root;Bacteria;Firmicutes;Clostridia;Clostridiales;Lachnospiraceae;Anaerostipes;Unclassified; 16 4 1 0

root;Bacteria;Firmicutes;Clostridia;Clostridiales;Lachnospiraceae;Lachnospira; 1 5 1 0

root;Bacteria;Firmicutes;Clostridia;Clostridiales;Lachnospiraceae;Coprococcus; 7 19 1 0

root;Bacteria;Firmicutes;Clostridia;Clostridiales;Lachnospiraceae;Coprococcus;Unclassified; 7 19 1 0

root;Bacteria;Firmicutes;Clostridia;Clostridiales;Lachnospiraceae;Unclassified; 11 6 0 0

root;Bacteria;Firmicutes;Clostridia;Clostridiales;Lachnospiraceae;Pseudobutyrivibrio; 14 1 0 0

root;Bacteria;Firmicutes;Clostridia;Clostridiales;Lachnospiraceae;Pseudobutyrivibrio;Unclassified; 14 1 0 0

root;Bacteria;Firmicutes;Clostridia;Clostridiales;Lachnospiraceae;Lachnobacterium; 4 3 0 0

root;Bacteria;Firmicutes;Clostridia;Clostridiales;Lachnospiraceae;Sporobacterium; 0 1 0 0

root;Bacteria;Firmicutes;Clostridia;Clostridiales;Heliobacteriaceae; 12 0 0 0

root;Bacteria;Firmicutes;Clostridia;Clostridiales;Heliobacteriaceae;Unclassified; 12 0 0 0

root;Bacteria;Firmicutes;Clostridia;Clostridiales;Natronoanaerobium; 3 4 0 0

root;Bacteria;Firmicutes;Clostridia;Clostridiales;Natronoanaerobium;Unclassified; 3 4 0 0

root;Bacteria;Firmicutes;Clostridia;Clostridiales;Ruminococcaceae; 9 0 0 0

root;Bacteria;Firmicutes;Clostridia;Clostridiales;Ruminococcaceae;Ruminococcus; 9 0 0 0

root;Bacteria;Firmicutes;Clostridia;Clostridiales;Ruminococcaceae;Ruminococcus;Unclassified; 9 0 0 0

root;Bacteria;Firmicutes;Clostridia;Clostridiales;Sedis; 9 5 2 0

root;Bacteria;Firmicutes;Clostridia;Clostridiales;Sedis;Thermaerobacter; 9 5 2 0

root;Bacteria;Firmicutes;Clostridia;Clostridiales;Syntrophomonadaceae; 13 2 5 1

root;Bacteria;Firmicutes;Clostridia;Clostridiales;Syntrophomonadaceae;Syntrophomonas; 6 2 5 1

root;Bacteria;Firmicutes;Clostridia;Clostridiales;Syntrophomonadaceae;Syntrophomonas;Unclassified; 6 2 5 1

root;Bacteria;Firmicutes;Clostridia;Clostridiales;Syntrophomonadaceae;Thermaerobacter; 1 0 0 0

root;Bacteria;Firmicutes;Clostridia;Clostridiales;Syntrophomonadaceae;Syntrophospora; 5 0 0 0

root;Bacteria;Firmicutes;Clostridia;Clostridiales;Syntrophomonadaceae;Caldicellulosiruptor; 1 0 0 0

root;Bacteria;Firmicutes;Clostridia;Clostridiales;Oscillospiraceae; 2 1 0 0

root;Bacteria;Firmicutes;Clostridia;Clostridiales;Oscillospiraceae;Oscillibacter; 2 1 0 0

root;Bacteria;Firmicutes;Clostridia;Clostridiales;group; 1 0 0 0

root;Bacteria;Firmicutes;Clostridia;Clostridiales;group;Unclassified; 1 0 0 0

root;Bacteria;Firmicutes;Clostridia;Thermoanaerobacteriales; 90 46 8 1

root;Bacteria;Firmicutes;Clostridia;Thermoanaerobacteriales;Thermoanaerobacteriaceae; 90 46 8 1

root;Bacteria;Firmicutes;Clostridia;Thermoanaerobacteriales;Thermoanaerobacteriaceae;group; 56 19 1 1

root;Bacteria;Firmicutes;Clostridia;Thermoanaerobacteriales;Thermoanaerobacteriaceae;group;Moorella; 56 19 1 1

root;Bacteria;Firmicutes;Clostridia;Thermoanaerobacteriales;Thermoanaerobacteriaceae;group;Moorella;Unclassified; 56 19 1 1

root;Bacteria;Firmicutes;Clostridia;Thermoanaerobacteriales;Thermoanaerobacteriaceae;Thermoanaerobacter; 17 14 5 0

root;Bacteria;Firmicutes;Clostridia;Thermoanaerobacteriales;Thermoanaerobacteriaceae;Thermoanaerobacter;Unclassified; 17 14 5 0

root;Bacteria;Firmicutes;Clostridia;Thermoanaerobacteriales;Thermoanaerobacteriaceae;Thermovenabulum; 15 13 2 0

root;Bacteria;Firmicutes;Clostridia;Thermoanaerobacteriales;Thermoanaerobacteriaceae;Thermovenabulum;Unclassified; 15 13 2 0

root;Bacteria;Firmicutes;Clostridia;Thermoanaerobacteriales;Thermoanaerobacteriaceae;Thermosediminibacter; 1 0 0 0

root;Bacteria;Firmicutes;Clostridia;Thermoanaerobacteriales;Thermoanaerobacteriaceae;Thermoanaerobacterium; 1 0 0 0

root;Bacteria;Firmicutes;Clostridia;Halanaerobiales; 8 2 3 0

root;Bacteria;Firmicutes;Clostridia;Halanaerobiales;Halanaerobiaceae; 8 2 3 0

root;Bacteria;Firmicutes;Clostridia;Halanaerobiales;Halanaerobiaceae;Halanaerobium; 8 2 3 0

root;Bacteria;Firmicutes;Clostridia;Unclassified; 1 0 0 0

root;Bacteria;Firmicutes;Clostridia;Natranaerobiales; 0 0 1 0

root;Bacteria;Firmicutes;Clostridia;Natranaerobiales;Natranaerobiaceae; 0 0 1 0

root;Bacteria;Firmicutes;Clostridia;Natranaerobiales;Natranaerobiaceae;Natranaerobius; 0 0 1 0

root;Bacteria;Firmicutes;Unclassified; 137 43 15 4

root;Bacteria;Firmicutes;Mollicutes; 256 137 22 10

root;Bacteria;Firmicutes;Mollicutes;Anaeroplasmatales; 147 46 3 3

root;Bacteria;Firmicutes;Mollicutes;Anaeroplasmatales;Erysipelotrichaceae; 147 46 3 3

root;Bacteria;Firmicutes;Mollicutes;Anaeroplasmatales;Erysipelotrichaceae;Erysipelothrix; 125 35 3 3

root;Bacteria;Firmicutes;Mollicutes;Anaeroplasmatales;Erysipelotrichaceae;Erysipelothrix;Unclassified; 125 35 3 3

root;Bacteria;Firmicutes;Mollicutes;Anaeroplasmatales;Erysipelotrichaceae;Bulleidia; 22 11 0 0

root;Bacteria;Firmicutes;Mollicutes;Anaeroplasmatales;Erysipelotrichaceae;Bulleidia;Unclassified; 22 11 0 0

root;Bacteria;Firmicutes;Mollicutes;Acholeplasmatales; 16 1 1 0

root;Bacteria;Firmicutes;Mollicutes;Acholeplasmatales;Acholeplasmataceae; 16 1 1 0

root;Bacteria;Firmicutes;Mollicutes;Acholeplasmatales;Acholeplasmataceae;Acholeplasma; 16 1 1 0

root;Bacteria;Firmicutes;Mollicutes;Acholeplasmatales;Acholeplasmataceae;Acholeplasma;Unclassified; 16 1 1 0

root;Bacteria;Firmicutes;Mollicutes;Entomoplasmatales; 3 29 7 0

root;Bacteria;Firmicutes;Mollicutes;Entomoplasmatales;Spiroplasmataceae; 3 29 7 0

root;Bacteria;Firmicutes;Mollicutes;Entomoplasmatales;Spiroplasmataceae;Spiroplasma; 3 29 7 0

root;Bacteria;Firmicutes;Mollicutes;Entomoplasmatales;Spiroplasmataceae;Spiroplasma;Unclassified; 3 29 7 0

root;Bacteria;Firmicutes;Mollicutes;Mycoplasmataceae; 35 14 4 7

root;Bacteria;Firmicutes;Mollicutes;Mycoplasmataceae;Mycoplasma; 35 14 3 7

root;Bacteria;Firmicutes;Mollicutes;Mycoplasmataceae;Mycoplasma;Unclassified; 35 14 3 7

root;Bacteria;Firmicutes;Mollicutes;Mycoplasmataceae;Ureaplasma; 0 0 1 0

root;Bacteria;Firmicutes;Mollicutes;Mycoplasmataceae;Ureaplasma;Unclassified; 0 0 1 0

root;Bacteria;Firmicutes;Mollicutes;Unclassified; 55 47 7 0

root;Bacteria;Unclassified; 1404 382 108 167

root;Bacteria;Actinobacteria; 3973 697 30 97

root;Bacteria;Actinobacteria;Actinobacteridae; 3759 569 19 90

root;Bacteria;Actinobacteria;Actinobacteridae;Actinomycetales; 3647 548 19 89

root;Bacteria;Actinobacteria;Actinobacteridae;Actinomycetales;Micrococcineae; 870 180 9 39

root;Bacteria;Actinobacteria;Actinobacteridae;Actinomycetales;Micrococcineae;Intrasporangiaceae; 64 12 2 0

root;Bacteria;Actinobacteria;Actinobacteridae;Actinomycetales;Micrococcineae;Intrasporangiaceae;Tetrasphaera; 64 11 2 0

root;Bacteria;Actinobacteria;Actinobacteridae;Actinomycetales;Micrococcineae;Intrasporangiaceae;II; 0 1 0 0

root;Bacteria;Actinobacteria;Actinobacteridae;Actinomycetales;Micrococcineae;Micrococcaceae; 468 97 8 27

root;Bacteria;Actinobacteria;Actinobacteridae;Actinomycetales;Micrococcineae;Micrococcaceae;Arthrobacter; 463 97 8 26

root;Bacteria;Actinobacteria;Actinobacteridae;Actinomycetales;Micrococcineae;Micrococcaceae;Arthrobacter;Unclassified; 463 97 8 26

root;Bacteria;Actinobacteria;Actinobacteridae;Actinomycetales;Micrococcineae;Micrococcaceae;Micrococcus; 5 1 0 0

root;Bacteria;Actinobacteria;Actinobacteridae;Actinomycetales;Micrococcineae;Micrococcaceae;Micrococcus;Unclassified; 5 1 0 0

root;Bacteria;Actinobacteria;Actinobacteridae;Actinomycetales;Micrococcineae;Microbacteriaceae; 172 63 1 7

root;Bacteria;Actinobacteria;Actinobacteridae;Actinomycetales;Micrococcineae;Microbacteriaceae;Pseudoclavibacter; 75 53 1 5

root;Bacteria;Actinobacteria;Actinobacteridae;Actinomycetales;Micrococcineae;Microbacteriaceae;Pseudoclavibacter;Unclassified; 75 53 1 5

root;Bacteria;Actinobacteria;Actinobacteridae;Actinomycetales;Micrococcineae;Microbacteriaceae;Microbacterium; 25 5 0 0

root;Bacteria;Actinobacteria;Actinobacteridae;Actinomycetales;Micrococcineae;Microbacteriaceae;Microbacterium;Unclassified; 25 5 0 0

root;Bacteria;Actinobacteria;Actinobacteridae;Actinomycetales;Micrococcineae;Microbacteriaceae;Curtobacterium; 7 0 0 0

root;Bacteria;Actinobacteria;Actinobacteridae;Actinomycetales;Micrococcineae;Microbacteriaceae;Curtobacterium;Unclassified; 7 0 0 0

root;Bacteria;Actinobacteria;Actinobacteridae;Actinomycetales;Micrococcineae;Microbacteriaceae;Cryocola; 28 0 0 0

root;Bacteria;Actinobacteria;Actinobacteridae;Actinomycetales;Micrococcineae;Microbacteriaceae;Unclassified; 37 5 2 0

root;Bacteria;Actinobacteria;Actinobacteridae;Actinomycetales;Micrococcineae;Promicromonosporaceae; 107 3 1 0

root;Bacteria;Actinobacteria;Actinobacteridae;Actinomycetales;Micrococcineae;Promicromonosporaceae;Isoptericola; 107 3 1 0

root;Bacteria;Actinobacteria;Actinobacteridae;Actinomycetales;Micrococcineae;Cellulomonadaceae; 54 5 2 0

root;Bacteria;Actinobacteria;Actinobacteridae;Actinomycetales;Micrococcineae;Cellulomonadaceae;Cellulomonas; 54 5 2 0

root;Bacteria;Actinobacteria;Actinobacteridae;Actinomycetales;Micrococcineae;Cellulomonadaceae;Cellulomonas;Unclassified; 54 5 2 0

root;Bacteria;Actinobacteria;Actinobacteridae;Actinomycetales;Micrococcineae;Brevibacteriaceae; 4 0 0 0

root;Bacteria;Actinobacteria;Actinobacteridae;Actinomycetales;Micrococcineae;Brevibacteriaceae;Brevibacterium; 4 0 0 0

root;Bacteria;Actinobacteria;Actinobacteridae;Actinomycetales;Micrococcineae;Brevibacteriaceae;Brevibacterium;Unclassified; 4 0 0 0

root;Bacteria;Actinobacteria;Actinobacteridae;Actinomycetales;Micrococcineae;Dermabacteraceae; 1 0 0 0

root;Bacteria;Actinobacteria;Actinobacteridae;Actinomycetales;Micrococcineae;Dermabacteraceae;Brachybacterium; 1 0 0 0

root;Bacteria;Actinobacteria;Actinobacteridae;Actinomycetales;Micrococcineae;Dermabacteraceae;Brachybacterium;Unclassified; 1 0 0 0

root;Bacteria;Actinobacteria;Actinobacteridae;Actinomycetales;Corynebacterineae; 408 103 4 25

root;Bacteria;Actinobacteria;Actinobacteridae;Actinomycetales;Corynebacterineae;Mycobacteriaceae; 170 54 4 13

root;Bacteria;Actinobacteria;Actinobacteridae;Actinomycetales;Corynebacterineae;Mycobacteriaceae;Mycobacterium; 170 54 4 13

root;Bacteria;Actinobacteria;Actinobacteridae;Actinomycetales;Corynebacterineae;Mycobacteriaceae;Mycobacterium;Unclassified; 170 54 4 13

root;Bacteria;Actinobacteria;Actinobacteridae;Actinomycetales;Corynebacterineae;Corynebacteriaceae; 209 49 12 0

root;Bacteria;Actinobacteria;Actinobacteridae;Actinomycetales;Corynebacterineae;Corynebacteriaceae;Corynebacterium; 209 49 12 0

root;Bacteria;Actinobacteria;Actinobacteridae;Actinomycetales;Corynebacterineae;Corynebacteriaceae;Corynebacterium;Unclassified; 209 49 12 0

root;Bacteria;Actinobacteria;Actinobacteridae;Actinomycetales;Corynebacterineae;Nocardiaceae; 29 0 0 0

root;Bacteria;Actinobacteria;Actinobacteridae;Actinomycetales;Corynebacterineae;Nocardiaceae;Rhodococcus; 29 0 0 0

root;Bacteria;Actinobacteria;Actinobacteridae;Actinomycetales;Corynebacterineae;Nocardiaceae;Rhodococcus;Unclassified; 29 0 0 0

root;Bacteria;Actinobacteria;Actinobacteridae;Actinomycetales;Pseudonocardineae; 151 7 0 0

root;Bacteria;Actinobacteria;Actinobacteridae;Actinomycetales;Pseudonocardineae;Actinosynnemataceae; 11 7 0 0

root;Bacteria;Actinobacteria;Actinobacteridae;Actinomycetales;Pseudonocardineae;Actinosynnemataceae;Actinokineospora; 5 7 0 0

root;Bacteria;Actinobacteria;Actinobacteridae;Actinomycetales;Pseudonocardineae;Actinosynnemataceae;Lechevalieria; 5 0 0 0

root;Bacteria;Actinobacteria;Actinobacteridae;Actinomycetales;Pseudonocardineae;Actinosynnemataceae;Lentzea; 1 0 0 0

root;Bacteria;Actinobacteria;Actinobacteridae;Actinomycetales;Pseudonocardineae;Pseudonocardiaceae; 140 0 0 0

root;Bacteria;Actinobacteria;Actinobacteridae;Actinomycetales;Pseudonocardineae;Pseudonocardiaceae;Saccharomonospora; 127 0 0 0

root;Bacteria;Actinobacteria;Actinobacteridae;Actinomycetales;Pseudonocardineae;Pseudonocardiaceae;Saccharopolyspora; 13 0 0 0

root;Bacteria;Actinobacteria;Actinobacteridae;Actinomycetales;Actinomycineae; 1883 171 11 0

root;Bacteria;Actinobacteria;Actinobacteridae;Actinomycetales;Actinomycineae;Actinomycetaceae; 1883 171 11 0

root;Bacteria;Actinobacteria;Actinobacteridae;Actinomycetales;Actinomycineae;Actinomycetaceae;Actinomyces; 1477 112 6 0

root;Bacteria;Actinobacteria;Actinobacteridae;Actinomycetales;Actinomycineae;Actinomycetaceae;Actinomyces;Unclassified; 1477 112 6 0

root;Bacteria;Actinobacteria;Actinobacteridae;Actinomycetales;Actinomycineae;Actinomycetaceae;Arcanobacterium; 286 38 3 0

root;Bacteria;Actinobacteria;Actinobacteridae;Actinomycetales;Actinomycineae;Actinomycetaceae;Arcanobacterium;Unclassified; 286 38 3 0

root;Bacteria;Actinobacteria;Actinobacteridae;Actinomycetales;Actinomycineae;Actinomycetaceae;group; 120 21 2 0

root;Bacteria;Actinobacteria;Actinobacteridae;Actinomycetales;Actinomycineae;Actinomycetaceae;group;Mobiluncus; 120 21 2 0

root;Bacteria;Actinobacteria;Actinobacteridae;Actinomycetales;Actinomycineae;Actinomycetaceae;group;Mobiluncus;Unclassified; 120 21 2 0

root;Bacteria;Actinobacteria;Actinobacteridae;Actinomycetales;Streptomycineae; 139 40 5 7

root;Bacteria;Actinobacteria;Actinobacteridae;Actinomycetales;Streptomycineae;Streptomycetaceae; 139 40 5 7

root;Bacteria;Actinobacteria;Actinobacteridae;Actinomycetales;Streptomycineae;Streptomycetaceae;Streptomyces; 133 39 5 6

root;Bacteria;Actinobacteria;Actinobacteridae;Actinomycetales;Streptomycineae;Streptomycetaceae;Streptomyces;Unclassified; 133 39 5 6

root;Bacteria;Actinobacteria;Actinobacteridae;Actinomycetales;Streptomycineae;Streptomycetaceae;Parastreptomyces; 5 1 0 0

root;Bacteria;Actinobacteria;Actinobacteridae;Actinomycetales;Streptomycineae;Streptomycetaceae;Streptacidiphilus; 1 1 0 0

root;Bacteria;Actinobacteria;Actinobacteridae;Actinomycetales;Propionibacterineae; 114 31 2 0

root;Bacteria;Actinobacteria;Actinobacteridae;Actinomycetales;Propionibacterineae;Nocardioidaceae; 99 18 1 0

root;Bacteria;Actinobacteria;Actinobacteridae;Actinomycetales;Propionibacterineae;Nocardioidaceae;Friedmanniella; 79 16 0 0

root;Bacteria;Actinobacteria;Actinobacteridae;Actinomycetales;Propionibacterineae;Nocardioidaceae;Pimelobacter; 19 1 0 0

root;Bacteria;Actinobacteria;Actinobacteridae;Actinomycetales;Propionibacterineae;Nocardioidaceae;Propionicimonas; 1 1 1 0

root;Bacteria;Actinobacteria;Actinobacteridae;Actinomycetales;Propionibacterineae;Propionibacteriaceae; 15 13 1 0

root;Bacteria;Actinobacteria;Actinobacteridae;Actinomycetales;Propionibacterineae;Propionibacteriaceae;Propionibacterium; 4 0 0 0

root;Bacteria;Actinobacteria;Actinobacteridae;Actinomycetales;Propionibacterineae;Propionibacteriaceae;Propionibacterium;Unclassified; 4 0 0 0

root;Bacteria;Actinobacteria;Actinobacteridae;Actinomycetales;Propionibacterineae;Propionibacteriaceae;Brooklawnia; 6 4 1 0

root;Bacteria;Actinobacteria;Actinobacteridae;Actinomycetales;Propionibacterineae;Propionibacteriaceae;Unclassified; 5 9 0 0

root;Bacteria;Actinobacteria;Actinobacteridae;Actinomycetales;Micromonosporineae; 48 5 1 0

root;Bacteria;Actinobacteria;Actinobacteridae;Actinomycetales;Micromonosporineae;Micromonosporaceae; 48 5 1 0

root;Bacteria;Actinobacteria;Actinobacteridae;Actinomycetales;Micromonosporineae;Micromonosporaceae;Salinispora; 43 5 0 0

root;Bacteria;Actinobacteria;Actinobacteridae;Actinomycetales;Micromonosporineae;Micromonosporaceae;Micromonospora; 1 0 0 0

root;Bacteria;Actinobacteria;Actinobacteridae;Actinomycetales;Micromonosporineae;Micromonosporaceae;Micromonospora;Unclassified; 1 0 0 0

root;Bacteria;Actinobacteria;Actinobacteridae;Actinomycetales;Micromonosporineae;Micromonosporaceae;Longispora; 4 1 0 0

root;Bacteria;Actinobacteria;Actinobacteridae;Actinomycetales;Glycomycineae; 9 2 2 0

root;Bacteria;Actinobacteria;Actinobacteridae;Actinomycetales;Glycomycineae;Glycomycetaceae; 9 2 2 0

root;Bacteria;Actinobacteria;Actinobacteridae;Actinomycetales;Glycomycineae;Glycomycetaceae;Glycomyces; 9 2 2 0

root;Bacteria;Actinobacteria;Actinobacteridae;Actinomycetales;Streptosporangineae; 23 7 2 0

root;Bacteria;Actinobacteria;Actinobacteridae;Actinomycetales;Streptosporangineae;Thermomonosporaceae; 10 6 1 0

root;Bacteria;Actinobacteria;Actinobacteridae;Actinomycetales;Streptosporangineae;Thermomonosporaceae;Thermomonospora; 8 5 1 0

root;Bacteria;Actinobacteria;Actinobacteridae;Actinomycetales;Streptosporangineae;Thermomonosporaceae;Actinocorallia; 2 1 0 0

root;Bacteria;Actinobacteria;Actinobacteridae;Actinomycetales;Streptosporangineae;Nocardiopsaceae; 13 1 1 0

root;Bacteria;Actinobacteria;Actinobacteridae;Actinomycetales;Streptosporangineae;Nocardiopsaceae;Streptomonospora; 12 1 0 0

root;Bacteria;Actinobacteria;Actinobacteridae;Actinomycetales;Streptosporangineae;Nocardiopsaceae;Nocardiopsis; 1 0 0 0

root;Bacteria;Actinobacteria;Actinobacteridae;Actinomycetales;Streptosporangineae;Nocardiopsaceae;Nocardiopsis;Unclassified; 1 0 0 0

root;Bacteria;Actinobacteria;Actinobacteridae;Actinomycetales;Streptosporangineae;Nocardiopsaceae;Unclassified; 0 0 0 1

root;Bacteria;Actinobacteria;Actinobacteridae;Actinomycetales;Frankineae; 2 2 1 0

root;Bacteria;Actinobacteria;Actinobacteridae;Actinomycetales;Frankineae;Acidothermaceae; 2 0 0 0

root;Bacteria;Actinobacteria;Actinobacteridae;Actinomycetales;Frankineae;Acidothermaceae;Acidothermus; 2 0 0 0

root;Bacteria;Actinobacteria;Actinobacteridae;Actinomycetales;Frankineae;Frankiaceae; 0 2 1 0

root;Bacteria;Actinobacteria;Actinobacteridae;Actinomycetales;Frankineae;Frankiaceae;Frankia; 0 2 1 0

root;Bacteria;Actinobacteria;Actinobacteridae;Actinomycetales;Frankineae;Frankiaceae;Frankia;Unclassified; 0 2 1 0

root;Bacteria;Actinobacteria;Actinobacteridae;Bifidobacteriales; 112 21 1 0

root;Bacteria;Actinobacteria;Actinobacteridae;Bifidobacteriales;Bifidobacteriaceae; 112 21 1 0

root;Bacteria;Actinobacteria;Actinobacteridae;Bifidobacteriales;Bifidobacteriaceae;Bifidobacterium; 112 21 1 0

root;Bacteria;Actinobacteria;Actinobacteridae;Bifidobacteriales;Bifidobacteriaceae;Bifidobacterium;Unclassified; 112 21 1 0

root;Bacteria;Actinobacteria;Coriobacteridae; 168 101 7 4

root;Bacteria;Actinobacteria;Coriobacteridae;Coriobacteriales; 168 101 7 4

root;Bacteria;Actinobacteria;Coriobacteridae;Coriobacteriales;Coriobacterineae; 168 101 7 4

root;Bacteria;Actinobacteria;Coriobacteridae;Coriobacteriales;Coriobacterineae;Coriobacteriaceae; 168 101 7 4

root;Bacteria;Actinobacteria;Coriobacteridae;Coriobacteriales;Coriobacterineae;Coriobacteriaceae;Denitrobacterium; 16 25 6 3

root;Bacteria;Actinobacteria;Coriobacteridae;Coriobacteriales;Coriobacterineae;Coriobacteriaceae;Atopobium; 142 61 1 0

root;Bacteria;Actinobacteria;Coriobacteridae;Coriobacteriales;Coriobacterineae;Coriobacteriaceae;Atopobium;Unclassified; 142 61 1 0

root;Bacteria;Actinobacteria;Coriobacteridae;Coriobacteriales;Coriobacterineae;Coriobacteriaceae;Collinsella; 7 13 1 0

root;Bacteria;Actinobacteria;Coriobacteridae;Coriobacteriales;Coriobacterineae;Coriobacteriaceae;Eggerthella; 1 2 0 0

root;Bacteria;Actinobacteria;Coriobacteridae;Coriobacteriales;Coriobacterineae;Coriobacteriaceae;Olsenella; 1 0 0 0

root;Bacteria;Actinobacteria;Coriobacteridae;Coriobacteriales;Coriobacterineae;Coriobacteriaceae;Olsenella;Unclassified; 1 0 0 0

root;Bacteria;Actinobacteria;Coriobacteridae;Coriobacteriales;Coriobacterineae;Coriobacteriaceae;Coriobacterium; 1 0 0 0

root;Bacteria;Actinobacteria;Microthrix; 3 1 0 0

root;Bacteria;Actinobacteria;Microthrix;Unclassified; 3 1 0 0

root;Bacteria;Actinobacteria;Rubrobacteridae; 36 19 4 2

root;Bacteria;Actinobacteria;Rubrobacteridae;Unclassified; 18 17 1 0

root;Bacteria;Actinobacteria;Rubrobacteridae;Rubrobacterales; 18 2 3 2

root;Bacteria;Actinobacteria;Rubrobacteridae;Rubrobacterales;Rubrobacterineae; 18 1 3 1

root;Bacteria;Actinobacteria;Rubrobacteridae;Rubrobacterales;Rubrobacterineae;Rubrobacterineae; 17 1 2 1

root;Bacteria;Actinobacteria;Rubrobacteridae;Rubrobacterales;Rubrobacterineae;Rubrobacteraceae; 1 0 0 0

root;Bacteria;Actinobacteria;Rubrobacteridae;Rubrobacterales;Rubrobacterineae;Rubrobacteraceae;Rubrobacter; 1 0 0 0

root;Bacteria;Actinobacteria;Rubrobacteridae;Rubrobacterales;Rubrobacterineae;Rubrobacteraceae;Rubrobacter;Unclassified; 1 0 0 0

root;Bacteria;Actinobacteria;Rubrobacteridae;Rubrobacterales;Rubrobacterineae;Thermoleophilaceae; 0 0 1 0

root;Bacteria;Actinobacteria;Rubrobacteridae;Rubrobacterales;Rubrobacterineae;Thermoleophilaceae;Thermoleophilum; 0 0 1 0

root;Bacteria;Actinobacteria;Rubrobacteridae;Rubrobacterales;Rubrobacterales; 0 1 1 0

root;Bacteria;Actinobacteria;Acidimicrobidae; 6 0 0 0

root;Bacteria;Actinobacteria;Acidimicrobidae;Unclassified; 6 0 0 0

root;Bacteria;Actinobacteria;Unclassified; 1 1 0 0

root;Bacteria;Actinobacteria;Symbiobacterium; 0 7 0 0

root;Bacteria;Bacteroidetes; 2026 1370 104 389

root;Bacteria;Bacteroidetes;Bacteroidia; 405 337 31 82

root;Bacteria;Bacteroidetes;Bacteroidia;Bacteroidales; 405 337 31 82

root;Bacteria;Bacteroidetes;Bacteroidia;Bacteroidales;Prevotellaceae; 229 8 29 0

root;Bacteria;Bacteroidetes;Bacteroidia;Bacteroidales;Prevotellaceae;Unclassified; 215 9 0 0

root;Bacteria;Bacteroidetes;Bacteroidia;Bacteroidales;Prevotellaceae;Paraprevotella; 14 8 20 0

root;Bacteria;Bacteroidetes;Bacteroidia;Bacteroidales;Porphyromonadaceae; 116 320 31 28

root;Bacteria;Bacteroidetes;Bacteroidia;Bacteroidales;Porphyromonadaceae;Porphyromonadaceae; 114 320 31 28

root;Bacteria;Bacteroidetes;Bacteroidia;Bacteroidales;Porphyromonadaceae;Parabacteroides; 2 0 0 0

root;Bacteria;Bacteroidetes;Bacteroidia;Bacteroidales;Porphyromonadaceae;Parabacteroides;Unclassified; 2 0 0 0

root;Bacteria;Bacteroidetes;Bacteroidia;Bacteroidales;Bacteroidaceae; 58 9 25 0

root;Bacteria;Bacteroidetes;Bacteroidia;Bacteroidales;Bacteroidaceae;Bacteroides; 58 9 25 0

root;Bacteria;Bacteroidetes;Bacteroidia;Bacteroidales;Bacteroidaceae;Bacteroides;Unclassified; 58 9 25 0

root;Bacteria;Bacteroidetes;Bacteroidia;Bacteroidales;Rikenellaceae; 2 0 0 0

root;Bacteria;Bacteroidetes;Bacteroidia;Bacteroidales;Rikenellaceae;Alistipes; 2 0 0 0

root;Bacteria;Bacteroidetes;Bacteroidia;Bacteroidales;Rikenellaceae;Alistipes;Unclassified; 2 0 0 0

root;Bacteria;Bacteroidetes;Flavobacteria; 113 23 3 48

root;Bacteria;Bacteroidetes;Flavobacteria;Unclassified; 5 4 3 3

root;Bacteria;Bacteroidetes;Flavobacteria;Flavobacteriales; 108 19 45 0

root;Bacteria;Bacteroidetes;Flavobacteria;Flavobacteriales;Flavobacteriaceae; 108 18 45 0

root;Bacteria;Bacteroidetes;Flavobacteria;Flavobacteriales;Flavobacteriaceae;Polaribacter; 13 2 0 0

root;Bacteria;Bacteroidetes;Flavobacteria;Flavobacteriales;Flavobacteriaceae;Polaribacter;Unclassified; 13 2 0 0

root;Bacteria;Bacteroidetes;Flavobacteria;Flavobacteriales;Flavobacteriaceae;Flavobacterium; 11 4 6 0

root;Bacteria;Bacteroidetes;Flavobacteria;Flavobacteriales;Flavobacteriaceae;Flavobacterium;Unclassified; 11 4 6 0

root;Bacteria;Bacteroidetes;Flavobacteria;Flavobacteriales;Flavobacteriaceae;Capnocytophaga; 7 8 22 0

root;Bacteria;Bacteroidetes;Flavobacteria;Flavobacteriales;Flavobacteriaceae;Capnocytophaga;Unclassified; 7 8 22 0

root;Bacteria;Bacteroidetes;Flavobacteria;Flavobacteriales;Flavobacteriaceae;Chryseobacterium; 19 2 2 0

root;Bacteria;Bacteroidetes;Flavobacteria;Flavobacteriales;Flavobacteriaceae;Chryseobacterium;Unclassified; 19 2 2 0

root;Bacteria;Bacteroidetes;Flavobacteria;Flavobacteriales;Flavobacteriaceae;Empedobacter; 1 1 2 0

root;Bacteria;Bacteroidetes;Flavobacteria;Flavobacteriales;Flavobacteriaceae;Unclassified; 22 4 0 0

root;Bacteria;Bacteroidetes;Flavobacteria;Flavobacteriales;Flavobacteriaceae;Wautersiella; 9 0 0 0

root;Bacteria;Bacteroidetes;Flavobacteria;Flavobacteriales;Flavobacteriaceae;Wautersiella;Unclassified; 9 0 0 0

root;Bacteria;Bacteroidetes;Flavobacteria;Flavobacteriales;Flavobacteriaceae;Leptobacterium; 17 1 1 0

root;Bacteria;Bacteroidetes;Flavobacteria;Flavobacteriales;Flavobacteriaceae;Pibocella; 3 3 0 0

root;Bacteria;Bacteroidetes;Flavobacteria;Flavobacteriales;Flavobacteriaceae;Zobellia; 1 0 0 0

root;Bacteria;Bacteroidetes;Flavobacteria;Flavobacteriales;Flavobacteriaceae;Psychroserpens; 1 1 0 0

root;Bacteria;Bacteroidetes;Flavobacteria;Flavobacteriales;Flavobacteriaceae;Galbibacter; 1 0 0 0

root;Bacteria;Bacteroidetes;Flavobacteria;Flavobacteriales;Flavobacteriaceae;Tenacibaculum; 1 0 0 0

root;Bacteria;Bacteroidetes;Flavobacteria;Flavobacteriales;Flavobacteriaceae;Tenacibaculum;Unclassified; 1 0 0 0

root;Bacteria;Bacteroidetes;Flavobacteria;Flavobacteriales;Flavobacteriaceae;Gelidibacter; 2 2 0 0

root;Bacteria;Bacteroidetes;Flavobacteria;Flavobacteriales;Flavobacteriaceae;Gelidibacter;Unclassified; 2 2 0 0

root;Bacteria;Bacteroidetes;Flavobacteria;Flavobacteriales;Flavobacteriaceae;Coccinistipes; 0 1 0 0

root;Bacteria;Bacteroidetes;Flavobacteria;Flavobacteriales;Flavobacteriaceae;Cellulophaga; 0 0 0 1

root;Bacteria;Bacteroidetes;Flavobacteria;Flavobacteriales;Flavobacteriaceae;Cellulophaga;Unclassified; 0 0 0 1

root;Bacteria;Bacteroidetes;Flavobacteria;Flavobacteriales;Cryomorphaceae; 0 1 0 0

root;Bacteria;Bacteroidetes;Flavobacteria;Flavobacteriales;Cryomorphaceae;Owenweeksia; 0 1 0 0

root;Bacteria;Bacteroidetes;Flavobacteria;Flavobacteriales;Cryomorphaceae;Owenweeksia;Unclassified; 0 1 0 0

root;Bacteria;Bacteroidetes;Sphingobacteria; 42 55 33 18

root;Bacteria;Bacteroidetes;Sphingobacteria;Sphingobacteriales; 42 54 33 18

root;Bacteria;Bacteroidetes;Sphingobacteria;Sphingobacteriales;Flexibacteraceae; 21 34 29 15

root;Bacteria;Bacteroidetes;Sphingobacteria;Sphingobacteriales;Flexibacteraceae;Cytophaga; 14 9 3 0

root;Bacteria;Bacteroidetes;Sphingobacteria;Sphingobacteriales;Flexibacteraceae;Cytophaga;Unclassified; 14 9 3 0

root;Bacteria;Bacteroidetes;Sphingobacteria;Sphingobacteriales;Flexibacteraceae;Marinicola; 2 0 0 0

root;Bacteria;Bacteroidetes;Sphingobacteria;Sphingobacteriales;Flexibacteraceae;Flexibacter; 3 9 27 9

root;Bacteria;Bacteroidetes;Sphingobacteria;Sphingobacteriales;Flexibacteraceae;Flexibacter;Unclassified; 3 9 27 9

root;Bacteria;Bacteroidetes;Sphingobacteria;Sphingobacteriales;Flexibacteraceae;Hongiella; 1 0 0 0

root;Bacteria;Bacteroidetes;Sphingobacteria;Sphingobacteriales;Flexibacteraceae;Hongiella;Unclassified; 1 0 0 0

root;Bacteria;Bacteroidetes;Sphingobacteria;Sphingobacteriales;Flexibacteraceae;Sporocytophaga; 1 14 2 1

root;Bacteria;Bacteroidetes;Sphingobacteria;Sphingobacteriales;Flexibacteraceae;Sporocytophaga;Unclassified; 1 14 2 1

root;Bacteria;Bacteroidetes;Sphingobacteria;Sphingobacteriales;Flexibacteraceae;Unclassified; 0 2 1 0

root;Bacteria;Bacteroidetes;Sphingobacteria;Sphingobacteriales;Flexibacteraceae;Arcicella; 0 0 0 1

root;Bacteria;Bacteroidetes;Sphingobacteria;Sphingobacteriales;Flexibacteraceae;Arcicella;Unclassified; 0 0 0 1

root;Bacteria;Bacteroidetes;Sphingobacteria;Sphingobacteriales;Sphingobacteriaceae; 5 8 2 2

root;Bacteria;Bacteroidetes;Sphingobacteria;Sphingobacteriales;Sphingobacteriaceae;Sphingobacterium; 3 2 2 0

root;Bacteria;Bacteroidetes;Sphingobacteria;Sphingobacteriales;Sphingobacteriaceae;Sphingobacterium;Unclassified; 3 2 2 0

root;Bacteria;Bacteroidetes;Sphingobacteria;Sphingobacteriales;Sphingobacteriaceae;Pedobacter; 2 4 2 0

root;Bacteria;Bacteroidetes;Sphingobacteria;Sphingobacteriales;Sphingobacteriaceae;Pedobacter;Unclassified; 2 4 2 0

root;Bacteria;Bacteroidetes;Sphingobacteria;Sphingobacteriales;Sphingobacteriaceae;Sphingoterrabacterium; 0 2 0 0

root;Bacteria;Bacteroidetes;Sphingobacteria;Sphingobacteriales;Sphingobacteriaceae;Sphingoterrabacterium;Unclassified; 0 2 0 0

root;Bacteria;Bacteroidetes;Sphingobacteria;Sphingobacteriales;Saprospiraceae; 12 6 1 0

root;Bacteria;Bacteroidetes;Sphingobacteria;Sphingobacteriales;Saprospiraceae;Lewinella; 7 2 0 0

root;Bacteria;Bacteroidetes;Sphingobacteria;Sphingobacteriales;Saprospiraceae;Lewinella;Unclassified; 7 2 0 0

root;Bacteria;Bacteroidetes;Sphingobacteria;Sphingobacteriales;Saprospiraceae;Saprospira; 5 0 0 0

root;Bacteria;Bacteroidetes;Sphingobacteria;Sphingobacteriales;Saprospiraceae;Unclassified; 0 4 1 0

root;Bacteria;Bacteroidetes;Sphingobacteria;Sphingobacteriales;Unclassified; 1 0 0 0

root;Bacteria;Bacteroidetes;Sphingobacteria;Sphingobacteriales;Crenotrichaceae; 1 6 1 0

root;Bacteria;Bacteroidetes;Sphingobacteria;Sphingobacteriales;Crenotrichaceae;Chitinophaga; 1 6 1 0

root;Bacteria;Bacteroidetes;Sphingobacteria;Sphingobacteriales;Crenotrichaceae;Chitinophaga;Unclassified; 1 6 1 0

root;Bacteria;Bacteroidetes;Sphingobacteria;Sphingobacteriales;Chitinophagaceae; 1 0 0 0

root;Bacteria;Bacteroidetes;Sphingobacteria;Sphingobacteriales;Chitinophagaceae;Sediminibacterium; 1 0 0 0

root;Bacteria;Bacteroidetes;Sphingobacteria;Sphingobacteriales;sedis; 1 0 0 0

root;Bacteria;Bacteroidetes;Sphingobacteria;Sphingobacteriales;sedis;Rhodothermus; 1 0 0 0

root;Bacteria;Bacteroidetes;Sphingobacteria;Sphingobacteriales;sedis;Rhodothermus;Unclassified; 1 0 0 0

root;Bacteria;Bacteroidetes;Sphingobacteria;Sphingobacteriales;Flammeovirgaceae; 0 0 1 0

root;Bacteria;Bacteroidetes;Sphingobacteria;Sphingobacteriales;Flammeovirgaceae;Persicobacter; 0 0 1 0

root;Bacteria;Bacteroidetes;Sphingobacteria;Unclassified; 0 1 0 0

root;Bacteria;Bacteroidetes;Bacteroidales; 1442 914 37 231

root;Bacteria;Bacteroidetes;Bacteroidales;Prevotellaceae; 869 397 18 78

root;Bacteria;Bacteroidetes;Bacteroidales;Prevotellaceae;Prevotella; 815 397 18 78

root;Bacteria;Bacteroidetes;Bacteroidales;Prevotellaceae;Prevotella;Unclassified; 815 397 18 78

root;Bacteria;Bacteroidetes;Bacteroidales;Prevotellaceae;Unclassified; 54 0 0 0

root;Bacteria;Bacteroidetes;Bacteroidales;Bacteroidaceae; 489 402 98 0

root;Bacteria;Bacteroidetes;Bacteroidales;Bacteroidaceae;Bacteroides; 489 402 98 0

root;Bacteria;Bacteroidetes;Bacteroidales;Bacteroidaceae;Bacteroides;Unclassified; 489 402 98 0

root;Bacteria;Bacteroidetes;Bacteroidales;Porphyromonadaceae; 55 24 1 42

root;Bacteria;Bacteroidetes;Bacteroidales;Porphyromonadaceae;Tannerella; 17 8 1 14

root;Bacteria;Bacteroidetes;Bacteroidales;Porphyromonadaceae;Tannerella;Unclassified; 17 8 1 14

root;Bacteria;Bacteroidetes;Bacteroidales;Porphyromonadaceae;Parabacteroides; 14 4 4 0

root;Bacteria;Bacteroidetes;Bacteroidales;Porphyromonadaceae;Parabacteroides;Unclassified; 14 4 4 0

root;Bacteria;Bacteroidetes;Bacteroidales;Porphyromonadaceae;Porphyromonas; 11 6 21 0

root;Bacteria;Bacteroidetes;Bacteroidales;Porphyromonadaceae;Porphyromonas;Unclassified; 11 6 21 0

root;Bacteria;Bacteroidetes;Bacteroidales;Porphyromonadaceae;Dysgonomonas; 9 6 0 0

root;Bacteria;Bacteroidetes;Bacteroidales;Porphyromonadaceae;Dysgonomonas;Unclassified; 9 6 0 0

root;Bacteria;Bacteroidetes;Bacteroidales;Porphyromonadaceae;Barnesiella; 2 0 0 0

root;Bacteria;Bacteroidetes;Bacteroidales;Porphyromonadaceae;Paludibacter; 2 3 0 0

root;Bacteria;Bacteroidetes;Bacteroidales;Rikenellaceae; 11 55 17 11

root;Bacteria;Bacteroidetes;Bacteroidales;Rikenellaceae;Alistipes; 8 48 17 8

root;Bacteria;Bacteroidetes;Bacteroidales;Rikenellaceae;Unclassified; 1 1 0 0

root;Bacteria;Bacteroidetes;Bacteroidales;Rikenellaceae;Ruminofilibacter; 2 7 2 0

root;Bacteria;Bacteroidetes;Bacteroidales;Unclassified; 18 36 1 2

root;Bacteria;Bacteroidetes;Unclassified; 23 41 10 0

root;Bacteria;Bacteroidetes;Niastella; 1 0 0 0

root;Bacteria;Fusobacteria; 752 139 10 64

root;Bacteria;Fusobacteria;Fusobacterales; 709 102 2 46

root;Bacteria;Fusobacteria;Fusobacterales;Fusobacteriaceae; 708 102 2 45

root;Bacteria;Fusobacteria;Fusobacterales;Fusobacteriaceae;Leptotrichia; 392 34 14 0

root;Bacteria;Fusobacteria;Fusobacterales;Fusobacteriaceae;Leptotrichia;Unclassified; 392 34 14 0

root;Bacteria;Fusobacteria;Fusobacterales;Fusobacteriaceae;Streptobacillus; 86 18 2 1

root;Bacteria;Fusobacteria;Fusobacterales;Fusobacteriaceae;Fusobacterium; 106 24 19 0

root;Bacteria;Fusobacteria;Fusobacterales;Fusobacteriaceae;Fusobacterium;Unclassified; 106 24 19 0

root;Bacteria;Fusobacteria;Fusobacterales;Fusobacteriaceae;Ilyobacter; 124 26 11 0

root;Bacteria;Fusobacteria;Fusobacterales;sedis; 1 1 0 0

root;Bacteria;Fusobacteria;Fusobacterales;sedis;Cetobacterium; 1 1 0 0

root;Bacteria;Fusobacteria;Fusobacteriales; 43 37 8 18

root;Bacteria;Fusobacteria;Fusobacteriales;Fusobacteriaceae; 43 37 8 18

root;Bacteria;Fusobacteria;Fusobacteriales;Fusobacteriaceae;Fusobacterium; 43 37 8 18

root;Bacteria;Fusobacteria;Fusobacteriales;Fusobacteriaceae;Fusobacterium;Unclassified; 43 37 8 18

root;Bacteria;Acidobacteria; 59 36 10 6

root;Bacteria;Acidobacteria;Acidobacteriales; 51 32 6 2

root;Bacteria;Acidobacteria;Acidobacteriales;Acidobacteriaceae; 51 32 6 2

root;Bacteria;Acidobacteria;Acidobacteriales;Acidobacteriaceae;Unclassified; 47 31 6 2

root;Bacteria;Acidobacteria;Acidobacteriales;Acidobacteriaceae;Holophaga; 4 1 0 0

root;Bacteria;Acidobacteria;Acidobacteriales;Acidobacteriaceae;Holophaga;Unclassified; 4 1 0 0

root;Bacteria;Acidobacteria;Unclassified; 6 4 4 4

root;Bacteria;Acidobacteria;Solibacteres; 2 0 0 0

root;Bacteria;Acidobacteria;Solibacteres;Solibacterales; 2 0 0 0

root;Bacteria;Acidobacteria;Solibacteres;Solibacterales;Solibacteraceae; 2 0 0 0

root;Bacteria;Acidobacteria;Solibacteres;Solibacterales;Solibacteraceae;Solibacter; 2 0 0 0

root;Bacteria;Cyanobacteria; 36 6 2 0

root;Bacteria;Cyanobacteria;Nostocales; 3 1 0 0

root;Bacteria;Cyanobacteria;Nostocales;Nostocaceae; 3 1 0 0

root;Bacteria;Cyanobacteria;Nostocales;Nostocaceae;Anabaena; 3 1 0 0

root;Bacteria;Cyanobacteria;Nostocales;Nostocaceae;Anabaena;Unclassified; 3 1 0 0

root;Bacteria;Cyanobacteria;Chroococcales; 18 0 0 0

root;Bacteria;Cyanobacteria;Chroococcales;Synechococcus; 8 0 0 0

root;Bacteria;Cyanobacteria;Chroococcales;Synechococcus;Unclassified; 8 0 0 0

root;Bacteria;Cyanobacteria;Chroococcales;Cyanobium; 10 0 0 0

root;Bacteria;Cyanobacteria;Chroococcales;Cyanobium;Unclassified; 10 0 0 0

root;Bacteria;Cyanobacteria;Prochlorales; 3 2 1 0

root;Bacteria;Cyanobacteria;Prochlorales;Prochlorococcaceae; 3 2 1 0

root;Bacteria;Cyanobacteria;Prochlorales;Prochlorococcaceae;Prochlorococcus; 3 2 1 0

root;Bacteria;Cyanobacteria;Prochlorales;Prochlorococcaceae;Prochlorococcus;Unclassified; 3 2 1 0

root;Bacteria;Cyanobacteria;Oscillatoriales; 11 2 0 0

root;Bacteria;Cyanobacteria;Oscillatoriales;Limnothrix; 9 0 0 0

root;Bacteria;Cyanobacteria;Oscillatoriales;Limnothrix;Unclassified; 9 0 0 0

root;Bacteria;Cyanobacteria;Oscillatoriales;Unclassified; 1 0 0 0

root;Bacteria;Cyanobacteria;Oscillatoriales;Leptolyngbya; 1 0 0 0

root;Bacteria;Cyanobacteria;Oscillatoriales;Leptolyngbya;Unclassified; 1 0 0 0

root;Bacteria;Cyanobacteria;Oscillatoriales;Microcoleus; 0 2 0 0

root;Bacteria;Cyanobacteria;Oscillatoriales;Microcoleus;Unclassified; 0 2 0 0

root;Bacteria;Cyanobacteria;Unclassified; 1 1 1 0

root;Bacteria;Spirochaetes; 106 11 4 8

root;Bacteria;Spirochaetes;Spirochaetales; 106 11 4 8

root;Bacteria;Spirochaetes;Spirochaetales;Spirochaetaceae; 99 10 4 7

root;Bacteria;Spirochaetes;Spirochaetales;Spirochaetaceae;Spirochaeta; 16 3 3 2

root;Bacteria;Spirochaetes;Spirochaetales;Spirochaetaceae;Spirochaeta;Unclassified; 16 3 3 2

root;Bacteria;Spirochaetes;Spirochaetales;Spirochaetaceae;Treponema; 80 7 1 4

root;Bacteria;Spirochaetes;Spirochaetales;Spirochaetaceae;Treponema;Unclassified; 80 7 1 4

root;Bacteria;Spirochaetes;Spirochaetales;Spirochaetaceae;Unclassified; 3 1 0 0

root;Bacteria;Spirochaetes;Spirochaetales;Sphaerochaeta; 4 0 0 0

root;Bacteria;Spirochaetes;Spirochaetales;Leptospiraceae; 3 1 1 0

root;Bacteria;Spirochaetes;Spirochaetales;Leptospiraceae;Leptospira; 3 1 1 0

root;Bacteria;Spirochaetes;Spirochaetales;Leptospiraceae;Leptospira;Unclassified; 3 1 1 0

root;Bacteria;Chlorobi; 3 2 0 0

root;Bacteria;Chlorobi;Chlorobia; 3 2 0 0

root;Bacteria;Chlorobi;Chlorobia;Chlorobiales; 3 2 0 0

root;Bacteria;Chlorobi;Chlorobia;Chlorobiales;Chlorobiaceae; 3 2 0 0

root;Bacteria;Chlorobi;Chlorobia;Chlorobiales;Chlorobiaceae;Chlorobaculum; 1 0 0 0

root;Bacteria;Chlorobi;Chlorobia;Chlorobiales;Chlorobiaceae;Chlorobaculum;Unclassified; 1 0 0 0

root;Bacteria;Chlorobi;Chlorobia;Chlorobiales;Chlorobiaceae;group; 2 2 0 0

root;Bacteria;Chlorobi;Chlorobia;Chlorobiales;Chlorobiaceae;group;Pelodictyon; 1 1 0 0

root;Bacteria;Chlorobi;Chlorobia;Chlorobiales;Chlorobiaceae;group;Chlorobium; 1 1 0 0

root;Bacteria;Chlorobi;Chlorobia;Chlorobiales;Chlorobiaceae;group;Chlorobium;Unclassified; 1 1 0 0

root;Bacteria;Chloroflexi; 40 23 9 3

root;Bacteria;Chloroflexi;Unclassified; 11 6 2 1

root;Bacteria;Chloroflexi;Chloroflexales; 24 11 7 1

root;Bacteria;Chloroflexi;Chloroflexales;Chloroflexaceae; 24 11 7 1

root;Bacteria;Chloroflexi;Chloroflexales;Chloroflexaceae;Roseiflexus; 7 2 0 0

root;Bacteria;Chloroflexi;Chloroflexales;Chloroflexaceae;Roseiflexus;Unclassified; 7 2 0 0

root;Bacteria;Chloroflexi;Chloroflexales;Chloroflexaceae;Chloroflexus; 17 9 7 1

root;Bacteria;Chloroflexi;Chloroflexales;Chloroflexaceae;Chloroflexus;Unclassified; 17 9 7 1

root;Bacteria;Chloroflexi;Dehalococcoidetes; 4 6 1 0

root;Bacteria;Chloroflexi;Dehalococcoidetes;Dehalococcoides; 4 6 1 0

root;Bacteria;Chloroflexi;Dehalococcoidetes;Dehalococcoides;Unclassified; 4 6 1 0

root;Bacteria;Chloroflexi;Herpetosiphonales; 1 0 0 0

root;Bacteria;Chloroflexi;Herpetosiphonales;Herpetosiphonaceae; 1 0 0 0

root;Bacteria;Chloroflexi;Herpetosiphonales;Herpetosiphonaceae;Herpetosiphon; 1 0 0 0

root;Bacteria;Ferribacter; 3 0 0 0

root;Bacteria;Gulbenkianus; 15 0 0 0

root;Bacteria;Thermus; 1 0 0 0

root;Bacteria;Thermus;Deinococci; 1 0 0 0

root;Bacteria;Thermus;Deinococci;Thermales; 1 0 0 0

root;Bacteria;Thermus;Deinococci;Thermales;Thermaceae; 1 0 0 0

root;Bacteria;Thermus;Deinococci;Thermales;Thermaceae;Meiothermus; 1 0 0 0

root;Bacteria;Thermus;Deinococci;Thermales;Thermaceae;Meiothermus;Meiothermus; 1 0 0 0

root;Bacteria;Aquificae; 1 0 0 0

root;Bacteria;Aquificae;Aquificales; 1 0 0 0

root;Bacteria;Aquificae;Aquificales;Desulfurobacterium; 1 0 0 0

root;Bacteria;Planctomycetes; 7 12 18 1

root;Bacteria;Planctomycetes;Planctomycetacia; 7 12 18 1

root;Bacteria;Planctomycetes;Planctomycetacia;Planctomycetales; 7 9 18 1

root;Bacteria;Planctomycetes;Planctomycetacia;Planctomycetales;Planctomycetaceae; 7 5 13 1

root;Bacteria;Planctomycetes;Planctomycetacia;Planctomycetales;Planctomycetaceae;Pirellula; 4 1 13 1

root;Bacteria;Planctomycetes;Planctomycetacia;Planctomycetales;Planctomycetaceae;Pirellula;Unclassified; 4 1 13 1

root;Bacteria;Planctomycetes;Planctomycetacia;Planctomycetales;Planctomycetaceae;Unclassified; 1 2 0 0

root;Bacteria;Planctomycetes;Planctomycetacia;Planctomycetales;Planctomycetaceae;Isosphaera; 1 0 0 0

root;Bacteria;Planctomycetes;Planctomycetacia;Planctomycetales;Planctomycetaceae;Isosphaera;Unclassified; 1 0 0 0

root;Bacteria;Planctomycetes;Planctomycetacia;Planctomycetales;Planctomycetaceae;Rhodopirellula; 1 2 0 0

root;Bacteria;Planctomycetes;Planctomycetacia;Planctomycetales;Brocadia; 0 3 0 0

root;Bacteria;Planctomycetes;Planctomycetacia;Planctomycetales;Unclassified; 0 1 5 0

root;Bacteria;Planctomycetes;Planctomycetacia;Planctomycetacia; 0 3 0 0

root;Bacteria;Gemmatimonadetes; 1 0 0 0

root;Bacteria;Gemmatimonadetes;Gemmatimonadales; 1 0 0 0

root;Bacteria;Gemmatimonadetes;Gemmatimonadales;Gemmatimonadaceae; 1 0 0 0

root;Bacteria;Gemmatimonadetes;Gemmatimonadales;Gemmatimonadaceae;Gemmatimonas; 1 0 0 0

root;Bacteria;Gemmatimonadetes;Gemmatimonadales;Gemmatimonadaceae;Gemmatimonas;Unclassified; 1 0 0 0

root;Bacteria;Deferribacteres; 1 2 0 0

root;Bacteria;Deferribacteres;Deferribacterales; 1 2 0 0

root;Bacteria;Deferribacteres;Deferribacterales;Deferribacteraceae; 1 2 0 0

root;Bacteria;Deferribacteres;Deferribacterales;Deferribacteraceae;Mucispirillum; 1 2 0 0

root;Bacteria;Verrucomicrobia; 1 1 1 0

root;Bacteria;Verrucomicrobia;Spartobacteria; 1 1 1 0

root;Bacteria;Verrucomicrobia;Spartobacteria;Unclassified; 1 1 1 0

root;Bacteria;Thermolithobacter; 1 0 0 0

root;Bacteria;Antarctic; 1 0 0 0

root;Bacteria;Nitrospirae; 0 3 0 0

root;Bacteria;Nitrospirae;Nitrospirales; 0 3 0 0

root;Bacteria;Nitrospirae;Nitrospirales;Nitrospiraceae; 0 3 0 0

root;Bacteria;Nitrospirae;Nitrospirales;Nitrospiraceae;Thermodesulfovibrio; 0 1 0 0

root;Bacteria;Nitrospirae;Nitrospirales;Nitrospiraceae;Thermodesulfovibrio;Unclassified; 0 1 0 0

root;Bacteria;Nitrospirae;Nitrospirales;Nitrospiraceae;Nitrospira; 0 2 0 0

root;Bacteria;Nitrospirae;Nitrospirales;Nitrospiraceae;Nitrospira;Unclassified; 0 2 0 0

root;Bacteria;Thermotogae; 0 1 0 0

root;Bacteria;Thermotogae;Thermotogales; 0 1 0 0

root;Bacteria;Thermotogae;Thermotogales;Thermotogaceae; 0 1 0 0

root;Bacteria;Thermotogae;Thermotogales;Thermotogaceae;Thermotoga; 0 1 0 0

root;Bacteria;Thermotogae;Thermotogales;Thermotogaceae;Thermotoga;Unclassified; 0 1 0 0

root;Bacteria;Tenericutes; 0 1 0 0

root;Bacteria;Tenericutes;Mollicutes; 0 1 0 0

root;Bacteria;Tenericutes;Mollicutes;Acholeplasmatales; 0 1 0 0

root;Bacteria;Tenericutes;Mollicutes;Acholeplasmatales;Acholeplasmataceae; 0 1 0 0

root;Bacteria;Tenericutes;Mollicutes;Acholeplasmatales;Acholeplasmataceae;Phytoplasma; 0 1 0 0

root;Bacteria;Ktedobacteria; 0 9 0 0

root;Bacteria;Ktedobacteria;Ktedobacterales; 0 7 0 0

root;Bacteria;Ktedobacteria;Ktedobacterales;Ktedobacteraceae; 0 7 0 0

root;Bacteria;Ktedobacteria;Ktedobacterales;Ktedobacteraceae;Ktedobacter; 0 7 0 0

root;Bacteria;Ktedobacteria;Ktedobacterales;Ktedobacteraceae;Ktedobacter;Ktedobacter; 0 7 0 0

root;Bacteria;Ktedobacteria;Ktedobacteria; 0 2 0 0

root;Bacteria;Tammella; 0 1 1 0

root;Bacteria;Fervidomicrobium; 0 0 1 0

root;Bacteria;Thermodesulfobacteria; 0 0 1 0

root;Bacteria;Thermodesulfobacteria;Thermodesulfobacteriales; 0 0 1 0

root;Bacteria;Thermodesulfobacteria;Thermodesulfobacteriales;Thermodesulfobacteriaceae; 0 0 1 0

root;Bacteria;Thermodesulfobacteria;Thermodesulfobacteriales;Thermodesulfobacteriaceae;Thermodesulfatator; 0 0 1 0

root;Eukaryota; 89 30 51 21

root;Eukaryota;Cryptophyta; 1 0 0 0

root;Eukaryota;Cryptophyta;Cryptomonadaceae; 1 0 0 0

root;Eukaryota;Cryptophyta;Cryptomonadaceae;Pyrenomonas; 1 0 0 0

root;Eukaryota;Metazoa; 22 11 47 18

root;Eukaryota;Metazoa;Mollusca; 22 11 47 18

root;Eukaryota;Metazoa;Mollusca;Bivalvia; 22 11 47 18

root;Eukaryota;Metazoa;Mollusca;Bivalvia;Heteroconchia; 22 11 47 18

root;Eukaryota;Metazoa;Mollusca;Bivalvia;Heteroconchia;Veneroida; 22 11 47 18

root;Eukaryota;Metazoa;Mollusca;Bivalvia;Heteroconchia;Veneroida;Glossoidea; 22 11 47 18

root;Eukaryota;Metazoa;Mollusca;Bivalvia;Heteroconchia;Veneroida;Glossoidea;Vesicomyidae; 22 11 47 18

root;Eukaryota;Metazoa;Mollusca;Bivalvia;Heteroconchia;Veneroida;Glossoidea;Vesicomyidae;Calyptogena; 22 11 47 18

root;Eukaryota;Viridiplantae; 56 1 0 0

root;Eukaryota;Viridiplantae;Streptophyta; 30 0 0 0

root;Eukaryota;Viridiplantae;Streptophyta;Embryophyta; 30 0 0 0

root;Eukaryota;Viridiplantae;Streptophyta;Embryophyta;Bryophyta; 5 0 0 0

root;Eukaryota;Viridiplantae;Streptophyta;Embryophyta;Bryophyta;Polytrichopsida; 4 0 0 0

root;Eukaryota;Viridiplantae;Streptophyta;Embryophyta;Bryophyta;Polytrichopsida;Polytrichales; 4 0 0 0

root;Eukaryota;Viridiplantae;Streptophyta;Embryophyta;Bryophyta;Polytrichopsida;Polytrichales;Polytrichaceae; 4 0 0 0

root;Eukaryota;Viridiplantae;Streptophyta;Embryophyta;Bryophyta;Polytrichopsida;Polytrichales;Polytrichaceae;Polytrichum; 4 0 0 0

root;Eukaryota;Viridiplantae;Streptophyta;Embryophyta;Bryophyta;Bryopsida; 1 0 0 0

root;Eukaryota;Viridiplantae;Streptophyta;Embryophyta;Bryophyta;Bryopsida;Funariidae; 1 0 0 0

root;Eukaryota;Viridiplantae;Streptophyta;Embryophyta;Bryophyta;Bryopsida;Funariidae;Funariales; 1 0 0 0

root;Eukaryota;Viridiplantae;Streptophyta;Embryophyta;Bryophyta;Bryopsida;Funariidae;Funariales;Funariaceae; 1 0 0 0

root;Eukaryota;Viridiplantae;Streptophyta;Embryophyta;Bryophyta;Bryopsida;Funariidae;Funariales;Funariaceae;Physcomitrella; 1 0 0 0

root;Eukaryota;Viridiplantae;Streptophyta;Embryophyta;Tracheophyta; 25 0 0 0

root;Eukaryota;Viridiplantae;Streptophyta;Embryophyta;Tracheophyta;Spermatophyta; 13 0 0 0

root;Eukaryota;Viridiplantae;Streptophyta;Embryophyta;Tracheophyta;Spermatophyta;Magnoliophyta; 13 0 0 0

root;Eukaryota;Viridiplantae;Streptophyta;Embryophyta;Tracheophyta;Spermatophyta;Magnoliophyta;eudicotyledons; 13 0 0 0

root;Eukaryota;Viridiplantae;Streptophyta;Embryophyta;Tracheophyta;Spermatophyta;Magnoliophyta;eudicotyledons;eudicotyledons; 10 0 0 0

root;Eukaryota;Viridiplantae;Streptophyta;Embryophyta;Tracheophyta;Spermatophyta;Magnoliophyta;eudicotyledons;eudicotyledons;asterids; 10 0 0 0

root;Eukaryota;Viridiplantae;Streptophyta;Embryophyta;Tracheophyta;Spermatophyta;Magnoliophyta;eudicotyledons;eudicotyledons;asterids;lamiids; 6 0 0 0

root;Eukaryota;Viridiplantae;Streptophyta;Embryophyta;Tracheophyta;Spermatophyta;Magnoliophyta;eudicotyledons;eudicotyledons;asterids;lamiids;Solanales; 6 0 0 0

root;Eukaryota;Viridiplantae;Streptophyta;Embryophyta;Tracheophyta;Spermatophyta;Magnoliophyta;eudicotyledons;eudicotyledons;asterids;lamiids;Solanales;Convolvulaceae; 6 0 0 0

root;Eukaryota;Viridiplantae;Streptophyta;Embryophyta;Tracheophyta;Spermatophyta;Magnoliophyta;eudicotyledons;eudicotyledons;asterids;lamiids;Solanales;Convolvulaceae;Cuscuteae; 6 0 0 0

root;Eukaryota;Viridiplantae;Streptophyta;Embryophyta;Tracheophyta;Spermatophyta;Magnoliophyta;eudicotyledons;eudicotyledons;asterids;lamiids;Solanales;Convolvulaceae;Cuscuteae;Cuscuta; 6 0 0 0

root;Eukaryota;Viridiplantae;Streptophyta;Embryophyta;Tracheophyta;Spermatophyta;Magnoliophyta;eudicotyledons;eudicotyledons;asterids;campanulids; 4 0 0 0

root;Eukaryota;Viridiplantae;Streptophyta;Embryophyta;Tracheophyta;Spermatophyta;Magnoliophyta;eudicotyledons;eudicotyledons;asterids;campanulids;Asterales; 4 0 0 0

root;Eukaryota;Viridiplantae;Streptophyta;Embryophyta;Tracheophyta;Spermatophyta;Magnoliophyta;eudicotyledons;eudicotyledons;asterids;campanulids;Asterales;Asteraceae; 4 0 0 0

root;Eukaryota;Viridiplantae;Streptophyta;Embryophyta;Tracheophyta;Spermatophyta;Magnoliophyta;eudicotyledons;eudicotyledons;asterids;campanulids;Asterales;Asteraceae;Cichorioideae; 4 0 0 0

root;Eukaryota;Viridiplantae;Streptophyta;Embryophyta;Tracheophyta;Spermatophyta;Magnoliophyta;eudicotyledons;eudicotyledons;asterids;campanulids;Asterales;Asteraceae;Cichorioideae;Cichorieae; 4 0 0 0

root;Eukaryota;Viridiplantae;Streptophyta;Embryophyta;Tracheophyta;Spermatophyta;Magnoliophyta;eudicotyledons;eudicotyledons;asterids;campanulids;Asterales;Asteraceae;Cichorioideae;Cichorieae;Lactuca; 4 0 0 0

root;Eukaryota;Viridiplantae;Streptophyta;Embryophyta;Tracheophyta;Spermatophyta;Magnoliophyta;eudicotyledons;Proteales; 3 0 0 0

root;Eukaryota;Viridiplantae;Streptophyta;Embryophyta;Tracheophyta;Spermatophyta;Magnoliophyta;eudicotyledons;Proteales;Platanaceae; 3 0 0 0

root;Eukaryota;Viridiplantae;Streptophyta;Embryophyta;Tracheophyta;Spermatophyta;Magnoliophyta;eudicotyledons;Proteales;Platanaceae;Platanus; 3 0 0 0

root;Eukaryota;Viridiplantae;Streptophyta;Embryophyta;Tracheophyta;Moniliformopses; 12 0 0 0

root;Eukaryota;Viridiplantae;Streptophyta;Embryophyta;Tracheophyta;Moniliformopses;Filicophyta; 7 0 0 0

root;Eukaryota;Viridiplantae;Streptophyta;Embryophyta;Tracheophyta;Moniliformopses;Filicophyta;Marattiopsida; 7 0 0 0

root;Eukaryota;Viridiplantae;Streptophyta;Embryophyta;Tracheophyta;Moniliformopses;Filicophyta;Marattiopsida;Marattiales; 7 0 0 0

root;Eukaryota;Viridiplantae;Streptophyta;Embryophyta;Tracheophyta;Moniliformopses;Filicophyta;Marattiopsida;Marattiales;Marattiaceae; 7 0 0 0

root;Eukaryota;Viridiplantae;Streptophyta;Embryophyta;Tracheophyta;Moniliformopses;Filicophyta;Marattiopsida;Marattiales;Marattiaceae;Angiopteris; 7 0 0 0

root;Eukaryota;Viridiplantae;Streptophyta;Embryophyta;Tracheophyta;Moniliformopses;Equisetophyta; 5 0 0 0

root;Eukaryota;Viridiplantae;Streptophyta;Embryophyta;Tracheophyta;Moniliformopses;Equisetophyta;Sphenopsida; 5 0 0 0

root;Eukaryota;Viridiplantae;Streptophyta;Embryophyta;Tracheophyta;Moniliformopses;Equisetophyta;Sphenopsida;Equisetales; 5 0 0 0

root;Eukaryota;Viridiplantae;Streptophyta;Embryophyta;Tracheophyta;Moniliformopses;Equisetophyta;Sphenopsida;Equisetales;Equisetaceae; 5 0 0 0

root;Eukaryota;Viridiplantae;Streptophyta;Embryophyta;Tracheophyta;Moniliformopses;Equisetophyta;Sphenopsida;Equisetales;Equisetaceae;Equisetum; 5 0 0 0

root;Eukaryota;Viridiplantae;Chlorophyta; 26 1 0 0

root;Eukaryota;Viridiplantae;Chlorophyta;Trebouxiophyceae; 26 1 0 0

root;Eukaryota;Viridiplantae;Chlorophyta;Trebouxiophyceae;Chlorellales; 26 1 0 0

root;Eukaryota;Viridiplantae;Chlorophyta;Trebouxiophyceae;Chlorellales;Chlorellaceae; 26 1 0 0

root;Eukaryota;Viridiplantae;Chlorophyta;Trebouxiophyceae;Chlorellales;Chlorellaceae;Auxenochlorella; 24 1 0 0

root;Eukaryota;Viridiplantae;Chlorophyta;Trebouxiophyceae;Chlorellales;Chlorellaceae;Chlorella; 2 0 0 0

root;Eukaryota;Rhodophyta; 7 17 3 0

root;Eukaryota;Rhodophyta;Florideophyceae; 7 17 3 0

root;Eukaryota;Rhodophyta;Florideophyceae;Palmariales; 3 17 3 0

root;Eukaryota;Rhodophyta;Florideophyceae;Palmariales;Palmariaceae; 3 17 3 0

root;Eukaryota;Rhodophyta;Florideophyceae;Palmariales;Palmariaceae;Palmaria; 3 17 3 0

root;Eukaryota;Rhodophyta;Florideophyceae;Ceramiales; 3 0 0 0

root;Eukaryota;Rhodophyta;Florideophyceae;Ceramiales;Ceramiaceae; 3 0 0 0

root;Eukaryota;Rhodophyta;Florideophyceae;Ceramiales;Ceramiaceae;Ceramium; 3 0 0 0

root;Eukaryota;Rhodophyta;Florideophyceae;Batrachospermales; 1 0 0 0

root;Eukaryota;Rhodophyta;Florideophyceae;Batrachospermales;Lemaneaceae; 1 0 0 0

root;Eukaryota;Rhodophyta;Florideophyceae;Batrachospermales;Lemaneaceae;Paralemanea; 1 0 0 0

root;Eukaryota;Euglenozoa; 1 1 0 0

root;Eukaryota;Euglenozoa;Euglenida; 1 1 0 0

root;Eukaryota;Euglenozoa;Euglenida;Euglenales; 1 1 0 0

root;Eukaryota;Euglenozoa;Euglenida;Euglenales;Lepocinclis; 1 0 0 0

root;Eukaryota;Euglenozoa;Euglenida;Euglenales;Monomorphina; 0 0 1 0

root;Eukaryota;stramenopiles; 2 1 3 0

root;Eukaryota;stramenopiles;Bacillariophyta; 2 1 3 0

root;Eukaryota;stramenopiles;Bacillariophyta;Coscinodiscophyceae; 2 1 3 0

root;Eukaryota;stramenopiles;Bacillariophyta;Coscinodiscophyceae;Thalassiosirophycidae; 1 1 3 0

root;Eukaryota;stramenopiles;Bacillariophyta;Coscinodiscophyceae;Thalassiosirophycidae;Thalassiosirales; 1 1 3 0

root;Eukaryota;stramenopiles;Bacillariophyta;Coscinodiscophyceae;Thalassiosirophycidae;Thalassiosirales;Thalassiosiraceae; 1 1 3 0

root;Eukaryota;stramenopiles;Bacillariophyta;Coscinodiscophyceae;Thalassiosirophycidae;Thalassiosirales;Thalassiosiraceae;Thalassiosira; 1 1 3 0

root;Eukaryota;stramenopiles;Bacillariophyta;Coscinodiscophyceae;Chaetocerotophycidae; 1 0 0 0

root;Eukaryota;stramenopiles;Bacillariophyta;Coscinodiscophyceae;Chaetocerotophycidae;Chaetocerotales; 1 0 0 0

root;Eukaryota;stramenopiles;Bacillariophyta;Coscinodiscophyceae;Chaetocerotophycidae;Chaetocerotales;Chaetocerotaceae; 1 0 0 0

root;Eukaryota;stramenopiles;Bacillariophyta;Coscinodiscophyceae;Chaetocerotophycidae;Chaetocerotales;Chaetocerotaceae;Chaetoceros; 1 0 0 0

root;sequences; 457 1 0 0

root;sequences;sequences; 457 0 0 0

root;sequences;sequences;vectors; 457 0 0 0

root;sequences;Unclassified; 0 1 0 0

root;Archaea; 16 1 0 0

root;Archaea;Euryarchaeota; 16 1 0 0

root;Archaea;Euryarchaeota;Thermoplasmata; 11 0 0 0

root;Archaea;Euryarchaeota;Thermoplasmata;Thermoplasmatales; 11 0 0 0

root;Archaea;Euryarchaeota;Thermoplasmata;Thermoplasmatales;Picrophilaceae; 11 0 0 0

root;Archaea;Euryarchaeota;Thermoplasmata;Thermoplasmatales;Picrophilaceae;Picrophilus; 11 0 0 0

root;Archaea;Euryarchaeota;Archaeoglobi; 5 0 0 0

root;Archaea;Euryarchaeota;Archaeoglobi;Archaeoglobales; 5 0 0 0

root;Archaea;Euryarchaeota;Archaeoglobi;Archaeoglobales;Archaeoglobaceae; 5 0 0 0

root;Archaea;Euryarchaeota;Archaeoglobi;Archaeoglobales;Archaeoglobaceae;Archaeoglobus; 5 0 0 0

root;Archaea;Euryarchaeota;Methanopyri; 0 0 0 1

root;Archaea;Euryarchaeota;Methanopyri;Methanopyrales; 0 0 0 1

root;Archaea;Euryarchaeota;Methanopyri;Methanopyrales;Methanopyraceae; 0 0 0 1

root;Archaea;Euryarchaeota;Methanopyri;Methanopyrales;Methanopyraceae;Methanopyrus; 0 0 0 1

root;Archaea;Euryarchaeota;Methanopyri;Methanopyrales;Methanopyraceae;Methanopyrus;Unclassified; 0 0 0 1

# Reference

1. Yang F, Zeng XW, Ning K, Liu KL, Lo CC, et al. (2012) Saliva microbiomes distinguish caries-active from healthy human populations. Isme Journal 6: 1-10.

2. Eppley JM, Tyson GW, Getz WM, Banfield JF (2007) Strainer: software for analysis of population variation in community genomic datasets. BMC Bioinformatics 8.

3. Hallin PF, Binnewies TT, Ussery DW (2008) The genome BLASTatlas - a GeneWiz extension for visualization of whole-genome homology. Molecular Biosystems 4: 363-371.

4. Markowitz VM, Chen IMA, Chu K, Szeto E, Palaniappan K, et al. (2012) IMG/M: the integrated metagenome data management and comparative analysis system. Nucleic Acids Research 40: D123-D129.

5. Su Xiaoquan XJ, Ning Kang (2011) Parallel-META: A High-Performance Computational Pipeline for Metagenomic Data Analysis. IEEE ISB: 173 - 178.

6. Ondov B, Bergman N, Phillippy A (2011) Interactive metagenomic visualization in a Web browser. BMC Bioinformatics 12: 385.

7. Letunic I, Bork P (2011) Interactive Tree Of Life v2: online annotation and display of phylogenetic trees made easy. Nucleic Acids Research 39: W475-W478.

8. Huson DH, Auch AF, Qi J, Schuster SC (2007) MEGAN analysis of metagenomic data. Genome Research 17: 377-386.

9. DeSantis TZ, Hugenholtz P, Larsen N, Rojas M, Brodie EL, et al. (2006) Greengenes, a chimera-checked 16S rRNA gene database and workbench compatible with ARB. Applied and Environmental Microbiology 72: 5069-5072.
